# Food Environment and Childhood Overweight/Obesity: A Systematic Review

**DOI:** 10.1016/j.advnut.2025.100564

**Published:** 2025-11-20

**Authors:** Yi Liu, Zhijun Yang, Taotao Deng, Chongjun Bi, Huabin Li, Pengfeng Qu, Yamin Chen, Dong Liang, Jiao Xu, Ning Li, Gangqiang Ding, Haijun Wang

**Affiliations:** 1 Department of Maternal and Child Health, School of Public Health, Peking University; National Health Commission Key Laboratory of Reproductive Health, Beijing, China; 2 China National Center for Food Safety Risk Assessment, Beijing, China; 3 Institute of Fruit Tree Research, Guangdong Academy of Agricultural Sciences; Key Laboratory of South Subtropical Fruit Biology and Genetic Resource Utilization, Ministry of Agriculture and Rural Affairs; Guangdong Provincial Key Laboratory of Science and Technology Research on Fruit Tree, Guangzhou, China; 4 UNICEF China Office, Beijing, China; 5 School of Public Health, Sun Yat-sen University, Guangzhou, China; 6 National Institute for Nutrition and Health, Chinese Center for Disease Control and Prevention, Beijing, China

**Keywords:** food environment, indicators, overweight, obesity, children

## Abstract

Childhood overweight/obesity has emerged as a pressing public health concern globally, and the impact of the food environment on children's diets and health outcomes has gained heightened attention. Comprehensive and child-specific monitoring systems are critical for guiding targeted interventions and policies. This review aimed to synthesize recent literature on food environment indicators associated with children with overweight/obesity using the 4A framework, including food availability, accessibility, affordability, and appeal. We conducted a systematic search of peer-reviewed literature (2020–2025). This systematic review is guided by Preferred Reporting Items for Systematic Reviews and Meta-Analyses and involved narrative synthesis and framework-based classification (CRD420251116187). A total of 75 observational and 6 intervention studies are included. Indicators related to availability (e.g., home food supply and fast food outlet density) and accessibility (e.g., proximity to healthy food stores) are most commonly studied, whereas affordability and appeal indicators (e.g., food pricing and marketing exposure) are less frequently addressed. Current evidence underscores deficiencies in the measurement and monitoring of the food environment for children, which is important to prevent and manage childhood overweight/obesity using integrated indicators at home, schools, communities, and society. Moreover, there is a necessity to develop a standardized, child-centered food environment monitoring system, facilitating prompt, equity-sensitive policy action to address children with overweight/obesity on a worldwide scale, which also supports global sustainable development. This systematic review paper will be useful for selecting indicators to construct a food environment monitoring system.

**Statements of Significance**

This systematic review addresses an important gap, as no previous review has comprehensively synthesized evidence on food environment indicators associated with childhood overweight/obesity. Using the 4A framework (food availability, food accessibility, food affordability, and food appeal) to organize existing evidence, this study provides a structured foundation for identifying key indicators that can inform the development of food environment monitoring systems and guide multilevel interventions across home, school, and community settings, thereby offering a practical reference for advancing food environment development initiatives worldwide.

## Introduction

Childhood overweight/obesity has been a global public health problem. By 2022, the overall number of overweight children aged <5 y reached 37 million, and >390 million children and adolescents aged 5–19 y were overweight, with 160 million with obesity [1,2]. In China, the prevalence of obesity among children and adolescents aged 7–18 y rose from 0.1% to 9.6% (75.6 times) between 1985 and 2019 [3]. Data from the China Chronic Disease and Nutrition Surveillance survey (2015–2019) indicated that the prevalence of overweight and obesity among children and adolescents aged 6–17 y and those <6 y was 19% and 10.4%, respectively [4].

Globalization, advances in science and technology, and agricultural systems developments in recent years have revolutionized dietary habits and food-related economic models, also the food environment worldwide. Food environment refers to the spaces where children and their families interact or engage with food. The interplay of physical, economic, political, and sociocultural contexts, along with opportunities and conditions, shapes food choices [5]. It covers the external environment that determines food availability, price, marketing, and advertising, as well as vendor and product characteristics [6]. It also includes the personal environment, encompassing purchasing power, access, convenience, affordability, and desirability. The role of the food environment on children's diet habits and connecting to noncommunicable diseases, such as obesity, is receiving increasing attention [7]. The conceptual dimensions of the food environment commonly include availability, accessibility, and affordability [8]. Appeal within the food environments that children are exposed to was also considered [9]. Appeal refers to the appeal of healthy compared with unhealthy food. Marketing practices across multiple channels and labeling influence consumers by conveying information about the healthiness of packaged products. Children are more susceptible to media and marketing influences than adults [10]. The increasing digitization of the food environment has further amplified the impact of digital marketing on children. Food marketing through various media channels (including television and the Internet) affects children's dietary attitudes, food preferences, and consumption behaviors, which subsequently would influence their eating behaviors and contribute to overweight and obesity [11]. To more systematically and comprehensively summarize food environment indicators relevant to childhood overweight/obesity, this review adopted the 4A framework—including food availability, food accessibility, food affordability, and food appeal [[12], [13], [14], [15], [16]]. This framework would help present existing evidence in a clear and structured manner, providing a comprehensive overview of how various dimensions of the food environment are associated with children's weight status.

Food environment monitoring is a strategy or system used to track the status of food environments within populations [17]. Implementing strong and consistent measures to effectively and continuously monitor changes in food environments, and timely collecting information on the food environments to which children are exposed, can promote the adoption of relevant policy actions by governments. This will improve the health of populations, particularly children, ensuring every child's right to nutrition. Traditional data sources in the food and nutrition field include national cohorts, community, or household surveys [18]. Emerging technologies, such as Geographic Information Systems, have expanded personalized data sources [19]. However, there is no food environment monitoring system especially tailored to children. Combing through relevant recent literature will help identify areas that require immediate measurement and monitoring, while concurrently supporting the construction of a food environment monitoring system for children, with the objective of mitigating the issue of childhood overweight/obesity.

Previous reviews have explored the relationships between food environment indicators and childhood overweight/obesity [20,21]. However, most have focused on isolated dimensions or specific indicators without applying a comprehensive or systematic framework [22,23]. Additionally, several reviews have centered on dietary behaviors rather than obesity outcomes, or targeted adult populations or the general population, rather than children specifically [[24], [25], [26]]. This paper aimed to systematically review the existing literature on food environment indicators using 4A framework, assisting home, schools, communities, and pertinent health management institutions in preventing and managing childhood overweight/obesity using integrated indicators, and will help some countries (such as China) to construct a food environment monitoring system for the prevention and management of children with overweight/obesity.

## Methods

### Search strategy for food environment indicators

To investigate how food environment indicators contribute to childhood overweight or obesity, a comprehensive systematic search was carried out. In this study, we define the food environment as follows: the spaces where children and their families interact or engage with food. Web of Science, PubMed, and Embase were the 3 databases we used to conduct the literature search. Within a 5-y timeframe, the following keywords were used in this search: ("food environment" OR "food availab∗" OR "food access∗" OR (("retail∗" OR "store∗" OR "shop∗") AND ("density" OR "distance")) OR "food afford∗" OR "food price" OR "food cost" OR "food subsidy" OR "food marketing" OR "food advertis∗") AND ("child∗" OR "adolescen∗") AND ("obes∗" OR "overweight" OR "BMI"). The predetermined search technique was carried out on 11 March, 2025; all the literature was managed in EndNote X9. This review has been registered with PROSPERO (CRD420251116187).

### Selection criteria and data extraction

**FIGURE 1:**
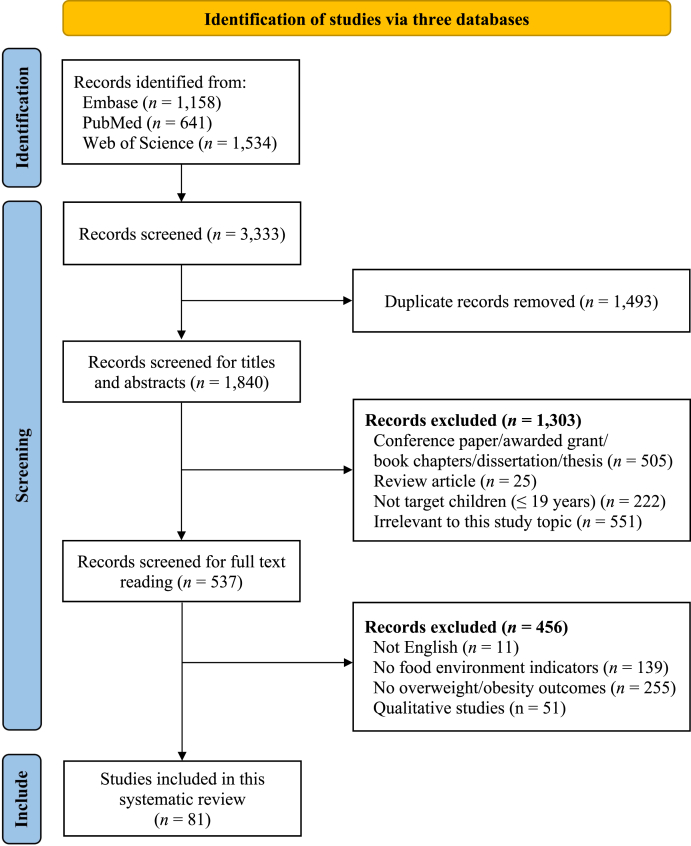
Flowchart for the literature search and screening procedure on food environment indicators and childhood overweight/obesity using PRISMA.

Titles and abstracts were initially screened by 1 reviewer (Y. L.), with the results independently verified by a second reviewer (Z. -J. Y.) to ensure accuracy and consistency. After removing duplicate records and irrelevant titles and abstracts, full-text articles were obtained and further assessed for final inclusion and data extraction. Data were extracted by 1 reviewer (Y. L.) and independently cross-checked by a second reviewer (Z. -J. Y.). Studies were eligible if: *1*) food environment indicators (including any 4A dimension: food availability, food accessibility, food affordability, and food appeal) were used in the research; *2*) the study population included children or adolescents (≤19 y); *3*) the study reported ≥1 anthropometric measurement, using BMI, BMI *z*-score, prevalence estimates of overweight/obesity, or other anthropometric metrics; *4*) the full-text articles were published in peer-reviewed journals in English. Research was not included if: *1*) it did not include any food environment indicator; *2*) it did not target children; *3*) it did not report outcomes related to overweight or obesity; *4*) it was not a journal article but rather a book chapter, dissertation, thesis, conference paper, or award grant; or *5*) it was a qualitative study without quantitative measurement of outcomes. The extracted information includes the following: *1*) study characteristics: title, first author's name, publication year, country, and sample size; *2*) food environment indicators: divided into 4A framework (food availability, accessibility, affordability, and appeal); and *3*) outcomes: overweight/obesity (Figure 1).

In this study, we define the 4A dimensions as follows: food availability addresses the "supply side" of food security, including the availability of foods at the household, community, and school levels, such as the quantity or presence of healthy and unhealthy foods in these settings [12,14]; accessibility refers to the physical aspects of obtaining, storing, and cooking food, typically measured by the distance to or density of nearby food retail stores [15]; affordability refers to the relative costs of healthy compared with unhealthy food, including food prices and subsidies [13]; appeal refers to the appeal of healthy compared with unhealthy food, influenced by marketing, advertising, and labeling practices [16]. This is affected by marketing practices through multiple channels and labeling, which provides consumers with information about the healthiness of packaged products.

## Results

### Literature search results and study characteristics

A total of 3333 articles were obtained from the Web of Science, PubMed, and Embase databases. The 81 original research articles were included after deduplication, screening of abstracts and titles, and full-text article reading. The reasons for the inclusion/exclusion of articles and screening flow are shown in Figure 1. The extracted data were evaluated using the narrative synthesis approach. The included studies were primarily carried out in the United States, United Kingdom, Brazil, and China, with study populations encompassing children across the full age spectrum from birth to 19 y (Table 1) [[27], [28], [29], [30], [31], [32], [33], [34], [35], [36], [37], [38], [39], [40], [41], [42], [43], [44], [45], [46], [47], [48], [49], [50], [51], [52], [53], [54], [55], [56], [57], [58], [59], [60], [61], [62], [63], [64], [65], [66], [67], [68], [69], [70], [71], [72], [73], [74], [75], [76], [77], [78], [79], [80], [81], [82], [83], [84], [85], [86], [87], [88], [89], [90], [91], [92], [93], [94], [95], [96], [97], [98], [99], [100], [101]]. The majority of studies utilized observational study designs, such as cross-sectional studies, prospective or retrospective cohort studies, and ecologic studies [62,71,75,93]. A smaller number of studies adopted cross-lagged panel analysis [34], case-control study [39], retrospective time-trend study [82], difference-in-differences design [83]. Modeling study [101] was also included, providing simulations of potential impacts under various scenarios.

**TABLE 1:** The associations between food environment indicators and childhood overweight/obesity from observational studies.

Study design	Country	Age	No. of participants	Food environment indicators	Overweight/obesity indicators	Covariates	Results	Ref.
1A: Food availability								
Availability of food at home (categorized as healthy and unhealthy, or use composite indicators)								
Healthy food at home								
Cross-sectional study	Six European countries (Belgium, Finland, Greece, Hungary, Bulgaria, Spain)	5.36–12 y old	12,041 children (5942 boys and 6099 girls)	Fruits availability at home	Overweight/obesity: using the International Obesity Task Force (IOTF) sex-age-specific cut-off points	Children's age, sex, regular exercise, maternal education, and country of residence	Overweight/obesity (rarely/never vs. always/often have fruits available at home): OR: 1.56; 95% CI: 1.07, 2.28; *P* = 0.020 ∗	[27]
Cross-sectional study	Six European countries (Belgium, Finland, Greece, Hungary, Bulgaria, Spain)	5.36–12 y old	12,041 children (5942 boys and 6099 girls)	Vegetables availability at home	Overweight/obesity: using the IOTF sex-age-specific cut-off points	Children's age, sex, regular exercise, maternal education, and country of residence	Overweight/obesity (rarely/never vs. always/often have vegetables available at home): OR: 1.21; 95% CI: 0.82, 1.79; *P* = 0.324	[27]
Cross-sectional study	Korea	Elementary school students: mean age of 10.4 y; Middle and high school students: mean age of 14.3 y	3148 students (1542 boys and 1606 girls)	Aspects of availability of fruit at home	Overweight: ≥85th, or ≥ BMI 25 kg/m^2^ or greater, based on gender and age	Fruit intake frequency	Elementary school: Boys (neutral or agree vs. disagree with the aspects of " fruits are available at home"): neutral OR: 0.87; 95% CI: 0.56, 1.34; agree OR: 0.79; 95% CI: 0.53, 1.18 Girls (neutral or agree vs. disagree with the aspects of " fruits are available at home"): neutral OR: 1.02; 95% CI: 0.64, 1.62; agree OR: 1.08; 95% CI: 0.72, 1.63 Middle and high school: Boys (neutral or agree vs. disagree with the aspects of " fruits are available at home"): neutral OR: 1.41; 95% CI: 0.69, 2.88; agree OR: 1.41; 95% CI: 0.72, 2.77 Girls (neutral or agree vs. disagree with the aspects of " fruits are available at home"): neutral OR: 0.61; 95% CI: 0.29, 1.27; agree OR: 0.70; 95% CI: 0.36, 1.37	[28]
Prospective observational study	Poland	5–11 y old	506 children	Parental perception of healthy food availability [including a lot of raw fruits and vegetables and limited amounts of products with added sugar or salt (e.g., salty or sweet snacks) and highly processed foods (e.g., sausage, cheese)] at home	Child excessive body mass status: refers to whether a child is classified as overweight or obese according to their BMI adjusted for age and gender	Parental education level, parental perceived economic status, and size of the place of residence	Between-groups differences for parent–child dyads with excessive body mass and with normal body mass: T1 (Cohen's *d*: 0.35; 95% CI: 0.32, 0.39; *P* < 0.001) ∗; T2 (Cohen's *d*: 0.42; 95% CI: 0.39, 0.45; *P* < 0.001) ∗	[29]
Prospective observational study	Poland	5–11 y old	506 children	Child perception of healthy food [including a lot of raw fruits and vegetables and limited amounts of products with added sugar or salt (e.g., salty or sweet snacks) and highly processed foods (e.g., sausage, cheese)] availability at home	Child excessive body mass status: refers to whether a child is classified as overweight or obese according to their BMI adjusted for age and gender	Parental education level, parental perceived economic status, and size of the place of residence	Between-groups differences for parent–child dyads with excessive body mass and with normal body mass: T1 (Cohen's *d*: –0.02; 95% CI: –0.06, 0.16; *P* > 0.05); T2 child excessive body mass status (Cohen's *d*: –0.20; 95% CI: –0.17, –0.23; *P* < 0.10)	[29]
Unhealthy food at home								
Cross-sectional study	Six European countries (Belgium, Finland, Greece, Hungary, Bulgaria, Spain)	5.36–12 y old	12,041 children (5942 boys and 6099 girls)	Sweets availability at home	Overweight/obesity: using the IOTF sex-age-specific cut-off points	Children's age, sex, regular exercise, maternal education, and country of residence	Overweight/obesity (rarely/never vs. always/often have sweets available at home): OR: 1.34; 95% CI: 1.14, 1.57; *P* < 0.001 ∗	[27]
Cross-sectional study	Six European countries (Belgium, Finland, Greece, Hungary, Bulgaria, Spain)	5.36–12 y old	12,041 children (5942 boys and 6099 girls)	Salty snacks availability at home	Overweight/obesity: using the IOTF sex-age-specific cut-off points	Children's age, sex, regular exercise, maternal education, and country of residence	Overweight/obesity (rarely/never vs. always/often have salty snacks available at home): OR: 1.21; 95% CI: 1.07, 1.38; *P* = 0.003 ∗	[27]
Prospective birth cohort study	United Kingdom	18–36 mo	1032 children at 18 mo and 986 children at 36 mo	Variety in home food availability of snack foods at 18 mo	BMI	Child's sex, child's age, mother's baseline age, mother's education, mother's baseline employment status, mother's ethnicity, and household size	BMI at 36 mo [high (6–7 items) or medium (4–5 items) vs. low (0–2 items) variety in home food availability of snack foods at 18 mo]: high (*β*: –0.28; 95% CI: –0.64, 0.07); medium (*β*: –0.28, 95% CI: –0.60, 0.03)	[30]
Prospective birth cohort study	United Kingdom	18–36 mo	1032 children at 18 mo and 986 children at 36 mo	Quantity for home food availability of snack foods at 18 mo	BMI	Child's sex, child's age, mother's baseline age, mother's education, mother's baseline employment status, mother's ethnicity, and household size	BMI at 36 mo (high (>2881 g) or medium (>1772 to ≤ 2881 g) vs. low (≤1772 g) quantity for home food availability of snack foods at 18 mo): high (*β*: –0.25; 95% CI: –0.53, 0.02); medium (*β*: –0.08; 95% CI: –0.34, 0.19)	[30]
Prospective birth cohort study	United Kingdom	18–36 mo	1032 children at 18 mo and 986 children at 36 mo	Variety in home food availability of sugar-sweetened beverages (SSBs) at 18 mo	BMI	Child's sex, child's age, mother's baseline age, mother's education, mother's baseline employment status, mother's ethnicity, and household size	BMI at 36 mo [high (3 items) or medium (2 items) vs. low (1 item) variety in home food availability of SSBs at 18 mo]: high (*β*: 0.01; 95% CI: –0.35, 0.38); medium (*β*: –0.20, 95% CI: –0.47, 0.08)	[30]
Prospective birth cohort study	United Kingdom	18–36 mo	1,032 children at 18 mo and 986 children at 36 mo	Quantity for home food availability of SSBs at 18 mo	BMI	Child's sex, child's age, mother's baseline age, mother's education, mother's baseline employment status, mother's ethnicity, and household size	BMI at 36 mo [high (>1747 mL) or medium (>654 to ≤1747 mL) vs. low (≤ 654 mL] quantity in home food availability of SSBs at 18 mo): high (*β*: –0.11, 95% CI: –0.37, 0.16); medium (*β*: –0.31, 95% CI: –0.58, –0.04) ∗	[30]
Retrospective longitudinal observational study	United States	13.12 ± 1.94 y old at T0 and 15.06 ± 1.98 y old at T1	82 adolescents at both T0 and T2, and 68 adolescents at T1	Availability of unhealthy food items at home: availability and frequency of unhealthy food choices (included chocolate candy; other candy; regular chips or crackers; baked chips/low-fat crackers/pretzels; raw vegetables; 100% fruit juice; juice drinks; regular sodas with sugar; diet or sugar-free sodas; sports drinks; fruit roll-ups or other dried fruit; regular or 2% milk; 1% or fat-free milk; sweetened breakfast cereal; unsweetened breakfast cereal)	OWOB: overweight (≥85th percentile but <the 95th percentile) and obesity (≥95th percentile)	—	T0: Normal weight (mean: 20.31, SE: 0.74), OWOB (mean: 20.15, SE: 0.82) T1: Normal weight (mean: 20.67, SE: 0.84), OWOB (mean: 20.42, SE: 0.87) T2: Normal weight (mean: 28.47, SE: 0.77), OWOB (mean: 27.89, SE: 0.86)	[31]
Composite indicators								
Cross-sectional study	Ecuador	Primary school students aged 5–12 y and secondary school students aged 13–17 y	19,249 school-age students, 12,632 primary school students and 6617 secondary school students	Food brought from home	Overweight: BMI-for-age >1 SD Obesity: BMI-for-age >2 SD	Child age, child sex, child ethnicity, child physical activity min/d, number of children in home, maternal education, household material resources, urbanity, and other school food sources	Primary school: Overweight (bring vs. not bring food from home): OR: 1.08, 95% CI: 0.90, 1.29 Obesity (bring vs. not bring food from home): OR: 0.96, 95% CI: 0.78, 1.18 Secondary school: Overweight (bring vs. not bring food from home): OR: 1.16, 95% CI: 0.87, 1.55 Obesity (bring vs. not bring food from home): OR: 0.77, 95% CI: 0.46, 1.28	[32]
Cross-sectional study	United States	6–17 y old	101 children (72 boys and 29 girls)	Unhealthy food index [how often healthy (e.g., raw fruits, vegetables, and low-fat crackers) and unhealthy foods (e.g., chocolate candy, cookies, and regular sodas) were available]	BMI percentile	Caregiver–child factors (caregiver age, caregiver BMI, caregiver country of origin, caregiver education, caregiver employment, caregiver marital status, caregiver insurance status, annual household income, home ownership, child intellectual and developmental disabilities primary diagnosis), parenting strategies, PA equipment index	BMI percentile: *β*: –0.37, *P* = 0.71	[33]
Cross-lagged panel analysis	United Kingdom	4–12 y old	298 children (145 boys and 153 girls)	Home food environment composite (21 variables)	BMI-SDS (SD Scores for child BMI)	Clustering within families and covariates, age of child at measurement, sex of child, and maternal BMI	Home food environment composite at age 4: BMI-SDS at age 4 (*β*: –0.16, 95% CI: –1.89, 0.26, *P* = 0.05); BMI-SDS at age 12 (*β*: 0.04, 95% CI: –0.11, 0.20, *P* = 0.59) Home food environment composite at age 12: BMI-SDS at age 12 (*β*: 0.04, 95% CI: –0.08, 0.19, *P* = 0.40)	[34]
Cross-sectional study	Indonesia	6–13 y old	1674 children (803 boys and 871 girls)	Obesogenic home food environment score	Overweight: BMI-for-age *z*-score above +1 SD Obese: BMI-for-age *z*-score above +2 SD BMI-for-age *z*-score	Child's sex, child's age, child's height, mother's years of education, child's dietary risk score, child's physical activity risk score	Overweight or obese: OR: 1.11, 95% CI: 1.00, 1.23, *P* < 0.05 ∗ BMI-for-age *z*-score at the 75th (*β*: 0.109, *P* < 0.05)∗; at the 90th (*β*: 0.110, *P* < 0.05)∗	[35]
Cross-sectional study	Korea	3–5 y old	364 children	Individual components of NQ-P score-Environment	Overweight or obesity (determined based on BMI percentiles from the 2017 Korean National Growth Chart)	Children's age, sex, and physical activity and mother's education, household income, and mother's BMI	Overweight or obese (highest quartile vs. lowest quartile of the Individual components of NQ-P score-Environment): OR: 0.36, 95% CI: 0.17, 0.71 ∗	[36]
Cross-sectional study	United States	12 or 13 y	815 children (427 boys and 388 girls)	Home food environment (measured using a subscale of the validated Comprehensive Feeding Practice Questionnaire)	Overweight or obese vs. normal weight: categorized based on the BMI-for-age and gender growth charts BMI *z*-score	Child's age in months, gender, race/ethnicity, highest education level among both parents, household income, marital status, and number of children in household, whether the family lives on-installation, parent's military rank, and months at current installation	Overweight or obese: OR: 0.96, 95% CI: 0.93, 1.00, *P* < 0.05 ∗ BMI z-score: *β*: –0.01, 95% CI: –0.03, –0.00, *P* < 0.05 ∗	[37]
Cross-sectional study	United Kingdom	12-y-old children (12.51 ± 0.22 y old)	298 children (145 boys and 153 girls)	Home food environment composite	BMI	Clustering within families (complex samples analyses), the child's age at time of home environment interview, child sex	BMI-SDS at age 12: *β*: 0.006, SE: 0.01, *P* = 0.674	[38]
Case-control study	Iran	14–18 y old	406 adolescents (210 boys and 196 girls)	Home food environment score (questions related to: TV or screen time; eating habits; the availability of snacks, junk foods, and soft drinks at home; how parents behave and guide the child for food selection and food eating)	Normal weight: defined as a BMI-for-age-and-sex percentile below the 85th Overweight: defined as a BMI-for-age-and-sex percentile between the 85th and 95th Obese: defined as a BMI-for-age-and-sex percentile above the 95th	—	Overweight/obese: mean difference: –1.8, *P* = 0.004 ∗	[39]
Household food security								
Cross-sectional study	Spain	2–14 y old	1938 children (976 boys and 962 girls)	Household food insecurity [HFI, measured by the Household Food Insecurity Access Scale (HFIAS)]	Overweight: defined as 1 SD < BMI *z*-score ≤ 2 SD Obesity: defined as BMI *z*-score > 2 SD	Age, country of origin of the mother, family purchasing power, employment status of the breadwinner, screen time, physical activity, and Healthy Eating Index	Overweight (household food insecurity vs. household food security): RRR: 2.41, 95% CI: 1.5, 4.0, *P* = 0.001 ∗ Obesity (household food insecurity vs. household food security): RRR: 1.99, 95% CI: 1.2, 3.4, *P* = 0.012 ∗	[40]
Cross-sectional study	Ethiopia	5–18 y old	632 children (306 boys and 326 girls)	Household food insecurity status: was measured using the Household Food Insecurity Access Scale of the Food and Nutrition Technical Assistance Project (HFIAS/FANTA)/United States Agency for International Development (USAID)	Overweight/obesity: >1 SD	Age group of the children and adolescents, maternal occupation, wealth index, age group of household head, maternal education	Overweight/obesity (household mildly and moderately food insecure vs. household food secure): OR: 1.36, 95% CI: 0.69, 2.71, *P* = 0.374 Overweight/obesity (household severely food insecure vs. household food secure): OR: 0.65, 95% CI: 0.30, 1.39, *P* = 0.269	[41]
Cross-sectional study	Ethiopia	5–18 y old	632 children (306 boys and 326 girls)	Child food insecurity status (measured by Children's Food Security Scale survey module)	Overweight/obesity: >1 SD	Age group of the children and adolescents, maternal occupation, wealth index, age group of household head, maternal education	Overweight/obesity (food insecure vs. food secure): OR: 0.78, 95% CI: 0.40, 1.52, *P* = 0.470	[41]
Cross-sectional study	United States	10–17 y old	68,942 children (35,506 boys and 33,436 girls)	Food insufficiency (if the family could not afford enough to eat sometimes or often)	Obesity or overweight: BMI was at or above the 85th percentile	Age, sex, family structure, highest level of education among reported adults, family poverty ratio, race/ethnicity, special healthcare needs, problems paying for health care, physically active days last week (60 + min), screentime	Obesity or overweight (food insufficiency vs. food sufficiency): OR: 1.25, 95% CI: 1.03, 1.52, *P* < 0.05 ∗	[42]
Cross-sectional study	United States	10–17 y old	68,942 children (35,506 boys and 33,436 girls)	Food insecurity (if the family always afford enough to eat but not always the kinds of food they should eat)	Obesity or overweight: BMI was at or above the 85th percentile	Age, sex, family structure, highest level of education among reported adults, family poverty ratio, race/ethnicity, special healthcare needs, problems paying for health care, physically active days last week (60 + min), screentime	Obesity or overweight (food insufficiency vs. food sufficiency): OR: 1.29, 95% CI: 1.16, 1.44 to *P* < 0.001 ∗	[42]
Schools meet health standards								
Cross-sectional study	United Kingdom	Aged 4 or 5 y, mean age 5.0 y (SD 0.4 y)	129,893 children (66,260 boys, 63,633 girls)	Percentage of healthy childcare setting	Obesity: ≥95th centile for age and sex	Low birth weight, breastfed at birth, fruit and vegetable consumption daily, and physically active daily for more than an hour	Obesity: OR: 1.000, 95% CI: 0.997, 1.004, *P* < 0.753	[43]
Cross-sectional study	Israel	First-grade students in Israel	Conventional: 205,500, Anthroposophical: 2247	School type: anthroposophical or conventional	Severe obesity: BMI >99th percentile Obesity: 97th < BMI ≤ 99th percentile Overweight: 97th ≤ BMI <85th percentile	—	Severe obesity: proportion (conventional: 4.9%, anthroposophical: 2.8%, *P* < 0.001) ∗ Obesity: proportion (conventional: 2.9%, anthroposophical: 2.0%, *P* = 0.007) ∗ Overweight: proportion (conventional: 11.2%, anthroposophical: 9.6%, *P* = 0.014) ∗	[44]
Cross-sectional study	Brazil	Second- and fourth-grade students, mean 8.0 y	1036 children (569 girls and 467 boys)	School Health Program (SHP) actions performed at school (healthy eating promotion activities)	Overweight	—	Overweight (SHP actions performed at school vs. SHP actions not be performed): OR: 1.72, 95% CI: 0.79, 3.75	[45]
Cross-sectional study	Brazil	Second- and fourth-grade students, mean 8.0 y	1036 children (569 girls and 467 boys)	SHP actions performed at school (nutritional state assessment in the school)	Overweight	—	Overweight (SHP actions performed at school vs. SHP actions not be performed): OR: 0.66, 95% CI: 0.45, 0.96 ∗	[45]
Cross-sectional study	Brazil	Second- and fourth-grade students, mean 8.0 y	1036 children (569 girls and 467 boys)	SHP actions performed at school (childhood obesity prevention activities)	Overweight	—	Overweight (SHP actions performed at school vs. SHP actions not be performed): OR: 0.42, 95% CI: 0.16, 1.01	[45]
Cross-sectional study	Brazil	12–17 y old	29,024 adolescents (14,599 males and 14,425 females)	Coverage by laws restricting food and beverage sales in school cafeteria	Obesity: BMI-for-age > Z+2	Individual sex, age, socioeconomic status (SES) and physical activity and breakfast habits	Presence of obesity in adolescents (all schools): OR: 0.89, 95% CI: 0.88, 0.91 ∗	[46]
Availability of food in school (categorized as healthy or unhealthy, and others)								
Healthy food in school								
Cross-sectional analysis of cohort baseline data	United States	Mean 16 y old, ranging from 14, 20 y old, with 98% between 15 and 17 y old	2263 children (1035 boys and 1228 girls)	School fruits and vegetables availability [fruits and vegetables availability score ranges from 0 (no fruits, vegetables or 100 % fruit juice offered any day) to 6 (fruits, vegetables and 100 % fruit juice offered every day)]	BMI	Race/ethnicity, parent education, family affluence, neighborhood land use mix, neighborhood population density and neighborhood poverty rate	1 km school food environment (SFE): *β*: 0.14, 95% CI: –0.12, 0.41 5 km SFE: *β*: 0.17, 95% CI: –0.10, 0.44	[47]
Cross-sectional analysis of cohort baseline data	United States	Mean 16 y old, ranging from 14, 20 y old, with 98% between 15 and 17 y old	2263 children (1035 boys and 1228 girls)	School fruits and vegetables availability [fruits and vegetables availability score ranges from 0 (no fruits, vegetables or 100 % fruit juice offered any day) to 6 (fruits, vegetables and 100 % fruit juice offered every day)] × all food outlets (fast food outlets, full-service restaurants, convenience stores, grocery stores/supermarkets)	BMI	Race/ethnicity, parent education, family affluence, neighborhood land use mix, neighborhood population density and neighborhood poverty rate	1 km SFE: *β*: –0.002, 95% CI: –0.01, 0.01 5 km SFE: *β*: –0.0003, 95% CI: –0.09, 0.03	[47]
Cross-sectional study	Korea	Elementary school students: mean age of 10.4 y. Middle and high school students: mean age of 14.3 y	3148 students (1542 boys and 1606 girls)	Aspects of regular provision of fruit in school lunches (whether the school provided fruit twice a week)	Overweight: ≥85th, or ≥ BMI 25 kg/m^2^ or greater, based on gender and age	Fruit intake frequency	Elementary school: Boys (neutral or agree vs. disagree with the aspects of " provision of fruit is regular in school lunches"): neutral OR: 0.87, 95% CI: 0.52, 1.47; agree OR: 0.70, 95% CI: 0.43, 1.15 Girls (neutral or agree vs. disagree with the aspects of " provision of fruit is regular in school lunches"): neutral OR: 0.72, 95% CI: 0.40, 1.31; agree OR: 0.54, 95% CI: 0.31, 0.95 ∗ Middle and high school: Boys (neutral or agree vs. disagree with the aspects of " provision of fruit is regular in school lunches"): neutral OR: 1.00, 95% CI: 0.50, 1.99; agree OR: 1.01, 95% CI: 0.52, 1.98 Girls (neutral or agree vs. disagree with the aspects of " provision of fruit is regular in school lunches"): neutral OR: 0.71, 95% CI: 0.32, 1.55; agree OR: 0.71, 95% CI: 0.34, 1.49	[28]
Cross-sectional study	Brazil	12–17 y old	2530 adolescents (1273 boys and 1257 girls)	Number of operational drinking fountains present in the school	Obesity: weight and height measurements above the z-score + 2	Sex, age, socioeconomic score, lives with the parents, how often the legal guardian has supper with the adolescent, breakfast-having frequency, vulnerability to health index, managerial dependence, ready-to-eat food shops within the 800 m buffer around the school	Obesity: OR: 0.91, 95% CI: 0.89, 0.93, *P* < 0.05 ∗	[48]
Cross-sectional study	United States	12 or 13 y	815 children (427 boys and 388 girls)	Number of healthy Competitive foods and beverages (CF&Bs) (included fruit, vegetables, water, milk) offered to students during the school day, outside of federally reimbursable and nutritionally regulated school meal programs	Overweight or obese vs. normal weight: categorized based on the BMI-for-age and gender growth charts BMI *z*-score	Child's age in months, gender, race/ethnicity, highest education level among both parents, household income, marital status, and number of children in household, whether the family lives on-installation, parent's military rank, and months at current installation	Overweight or obese: OR: 1.01, 95% CI: 0.82, 1.24 BMI *z*-score: *β*: 0.02, 95% CI: –0.06, 0.10	[37]
Unhealthy food in school								
Cross-sectional analysis of cohort baseline data	United States	Mean 16 y old, ranging from 14 to 20 y old, with 98% between 15 and 17 y old	2263 children (1035 boys and 1228 girls)	School snacks availability [snacks availability score ranges from 0 (no chocolate and candy, salty snacks or sweet snacks offered any day) to 6 (chocolate and candy, salty snacks and sweet snacks offered every day)]	BMI	Race/ethnicity, parent education, family affluence, neighborhood land use mix, neighborhood population density and neighborhood poverty rate	1 km SFE: *β*: –0.08, 95% CI: –0.34, 0.19 5 km SFE: *β*: –0.06, 95% CI: –0.32, 0.21	[47]
Cross-sectional analysis of cohort baseline data	United States	Mean 16 y old, ranging from 14 to 20 y old, with 98% between 15 and 17 y old	2263 children (1035 boys and 1228 girls)	School snacks availability [snacks availability score ranges from 0 (no chocolate and candy, salty snacks or sweet snacks offered any day) to 6 (chocolate and candy, salty snacks and sweet snacks offered every day)] × all food outlets (fast food outlets, full-service restaurants, convenience stores, grocery stores/supermarkets)	BMI	Race/ethnicity, parent education, family affluence, neighborhood land use mix, neighborhood population density, and neighborhood poverty rate	1 km SFE: *β*: –0.00001, 95% CI: –0.02, 0.02 5 km SFE: *β*: –0.0002, 95% CI: –0.001, 0.001	[47]
Cross-sectional analysis of cohort baseline data	United States	Mean 16 y old, ranging from 14 to 20 y old, with 98% between 15 and 17 y old	2263 children (1035 boys and 1228 girls)	School soda availability [soda availability score ranges from 0 (no soda offered any day) to 2 (soda offered every day)]	BMI	Race/ethnicity, parent education, family affluence, neighborhood land use mix, neighborhood population density, and neighborhood poverty rate	1 km SFE: *β*: 0.10, 95% CI: –0.43, 0.63 5 km SFE: *β*: 0.14, 95% CI: –0.39, 0.68	[47]
Cross-sectional analysis of cohort baseline data	United States	Mean 16 y old, ranging from 14 to 20 y old, with 98% between 15 and 17 y old	2263 children (1035 boys, and 1228 girls)	School Soda availability [soda availability score ranges from 0 (no soda offered any day) to 2 (soda offered every day)] × all food outlets (fast food outlets, full-service restaurants, convenience stores, grocery stores/supermarkets)	BMI	Race/ethnicity, parent education, family affluence, neighborhood land use mix, neighborhood population density, and neighborhood poverty rate	1 km SFE: *β*: –0.01, 95% CI: –0.03, 0.02 5 km SFE: *β*: –0.001, 95% CI: –0.003, 0.001	[47]
Cross-sectional study	United States	12 or 13 y	815 children (427 boys and 388 girls)	Number of unhealthy competitive foods and beverages (CF&Bs) (included French fries, SSBs, salty snacks, sweets, juice) offered to students during the school day, outside of federally reimbursable and nutritionally regulated school meal programs	Overweight or obese vs. normal weight: categorized based on the BMI-for-age and gender growth charts BMI *z*-score	Child's age in months, gender, race/ethnicity, highest education level among both parents, household income, marital status, and number of children in household, whether the family lives on-installation, parent's military rank, and months at current installation	Overweight or obese: OR: 1.01, 95% CI: 0.89, 1.14 BMI *z*-score: *β*: –0.01, 95% CI: –0.06, 0.05	[37]
Other indicators in school								
Cross-sectional study	United States	12 or 13 y	815 children (427 boys and 388 girls)	Number of CF&Bs offered to students during the school day, outside of federally reimbursable and nutritionally regulated school meal programs	Overweight or obese vs. normal weight: categorized based on the BMI-for-age and gender growth charts BMI *z*-score	Child's age in months, gender, race/ethnicity, highest education level among both parents, household income, marital status, and number of children in household, whether the family lives on-installation, parent's military rank, and months at current installation	Overweight or obese: OR: 0.98, 95% CI: 0.93, 1.03 BMI *z*-score: *β*: 0.00, 95% CI: –0.02, 0.03	[37]
Cross-sectional study	United States	12 or 13 y	815 children (427 boys and 388 girls)	Number of neutral CF&Bs (included sandwiches/burgers/pizza and bread) offered to students during the school day, outside of federally reimbursable and nutritionally regulated school meal programs	Overweight or obese vs. normal weight: categorized based on the BMI-for-age and gender growth charts BMI z-score	Child's age in months, gender, race/ethnicity, highest education level among both parents, household income, marital status, and number of children in household, whether the family lives on-installation, parent's military rank, and months at current installation	Overweight or obese: OR: 0.90, 95% CI: 0.71, 1.14 BMI *z*-score: *β*: 0.01, 95% CI: –0.09, 0.10	[37]
Cross-sectional study	Ecuador	Primary school students aged 5–12 y and secondary school students aged 13–17 y	19,249 school-age students, 12,632 primary school students, and 6617 secondary school students	Food purchased from school bar	Overweight: BMI-for-age >1 SD Obesity: BMI-for-age >2 SD	Child age, child sex, child ethnicity, child physical activity min/day, number of children in home, maternal education, household material resources, urbanity, and other school food sources	Primary school: Overweight (purchase vs. not purchase food from school bar): OR: 1.01, 95% CI: 0.83, 1.22 Obesity (purchase vs. not purchase food from school bar): OR: 1.05, 95% CI: 0.84, 1.31 Secondary school: Overweight (purchase vs. not purchase food from school bar): OR: 1.05, 95% CI: 0.78, 1.43 Obesity (purchase vs. not purchase food from school bar): OR: 1.17, 95% CI: 0.71, 1.92	[32]
Cross-sectional study	Ecuador	Primary school students aged 5–12 y and secondary school students aged 13–17 y	19,249 school-age students, 12,632 primary school students and 6617 secondary school students	Obtain food from School Breakfast Program (SBP)	Overweight: BMI-for-age >1 SD Obesity: BMI-for-age >2 SD	Child age, child sex, child ethnicity, child physical activity min/day, number of children in home, maternal education, household material resources, urbanity, and other school food sources	Primary school: Overweight (obtain vs. not obtain food from SBP): OR: 1.04, 95% CI: 0.88, 1.24 Obesity (obtain vs. not obtain food from SBP): OR: 0.76, 95% CI: 0.63, 0.91, *P* < 0.0001 ∗ Secondary school: Overweight (obtain vs. not obtain food from SBP): OR: 0.82, 95% CI: 0.65, 1.03 Obesity (obtain vs. not obtain food from SBP): OR: 0.83, 95% CI: 0.57, 1.21	[32]
Cross-sectional study	Brazil	Second-and fourth-grade students, mean 8.0 y	1036 children (569 girls and 467 boys)	Number of offered meals	Overweight	—	Overweight (3 meals offered vs. 1 meal offered): OR: 0.62, 95% CI: 0.47, 0.81 ∗	[45]
Longitudinal study	United States	From kindergarten (K) to 12th grade. The age range of the children in this study is ∼5–18 y old	106 schools	Environment type Healthier SFE: is characterized by a higher proportion of healthy options within the National School Lunch Program (NSLP) and in competitive foods (a la carte items and vending machines). This is measured by: NSLP Healthy Scale and Competitive Food Healthy Scale (90th percentile) Unhealthier SFE: is characterized by a lower proportion of healthy options in the NSLP and competitive foods (10th percentile) Low-density community food environment (CFE): refers to an area around the school with a lower number or presence of certain types of food outlets within a 400-m roadway network. On the basis of the composite healthier profile, a low-density CFE is suggested by: number of limited-service restaurants: 0 (10th percentile). Number of convenience stores: 0 (10th percentile). Presence of small grocery stores: 1 (yes). Presence of upgraded stores (healthy corner stores): 1 (yes) High-density CFE: refers to an area around the school with a higher number or presence of certain types of food outlets within a 400-m roadway network. On the basis of the composite unhealthier profile, a high-density CFE is suggested by: number of limited-service restaurants: 8 (90th percentile). Number of convenience stores: 3 (90th percentile). Presence of small grocery stores: 0 (No). Presence of upgraded stores: 0 (No)	Obesity rate	Time, school level, % of students eligible for free and reduced-price meals (FRPM), % of Hispanic students, % of non-Hispanic Black students	Unhealthy SFE; low-density CFE: obesity rate at baseline (*β*: –0.014, 95% CI: –0.067, 0.039, *P* = 0.611) difference in obesity rate trend (*β*: 0.001, 95% CI: –0.005, 0.007, *P* = 0.674) Healthy SFE; high-density CFE: obesity rate at baseline (*β*: 0.026, 95% CI: –0.006, 0.059, *P* = 0.115) difference in obesity rate trend (*β*: –0.010, 95% CI: –0.022, 0.001, *P* = 0.080) Unhealthy SFE; high-density CFE: obesity rate at baseline (*β*: –0.050, 95% CI: –0.096, –0.004, *P* = 0.033) ∗ difference in obesity rate trend (*β*: 0.014, 95% CI: –0.022, 0.001, *P* = 0.080)	[49]
Availability of food in community (categorized as healthy and unhealthy)								
Healthy food in community								
Cross-sectional (individual data) and partially ecologic (environmental component)	Brazil	Between 6 and 59 mo (<5 y old)	665 children (360 boys and 305 girls)	Perception of the availability of healthy food (fruits, vegetables, and low-fat foods) in the neighborhood	Overweight: WHZ > +2	Individual variables (woman: age and education; child: age and sex) and socioeconomic (per capita household income)	Presence of overweight (low perception vs. high perception of the availability of healthy food): OR: 0.77, 95% CI: 0.49, 1.22, *P* = 0.28	[50]
Retrospective cohort study	United States	42 d of birth through age 4 (inclusive)	148,634 children, boys: 43,546 (old package) + 32,195 (new package): 75,741 boys, girls: 41,686 (old package) + 31,207 (new package): 72,893 girls	New Special Supplemental Nutrition Program for Women, Infants and Children (WIC) food package	Obesity: BMI-for-age ≥ 95th percentile	Healthy food outlet density, unhealthy food outlet density, interactions between healthy and unhealthy food outlet densities, child race, initial WHZ (first length and weight measurement for each participant), age at last measurement, household income, maternal education and language preference, and neighborhood percent poverty, percent high school graduates, percent non-White and population density	WIC food package change (new WIC food package vs. old WIC food package) at healthiest food environment (highest density of healthy food outlets, lowest density of unhealthy food outlet density): obesity at age 4 in boys (RR: 0.82, 95% CI: 0.76, 0.90) ∗ obesity at age 4 in girls (RR: 0.85, 95% CI: 0.77, 0.93) ∗ WIC food package change (new WIC food package vs. old WIC food package) at median healthy and unhealthy food environment densities: obesity at age 4 in boys (RR: 0.91, 95% CI: 0.88, 0.95) ∗ obesity at age 4 in girls (RR: 0.95, 95% CI: 0.91, 0.98) ∗	[51]
Cross-sectional study	United States	5–9 y old	1296 families with children	Low perceived access to fruits/vegetables scale	Child pBMI	Area deprivation index, online shopping experience, shopping by car, number of adults, number of children, child's gender, primary caregiver's education, public assistance receipt, household income, race/ethnicity, eating American foods at home and time in the United States	Food-insecure households: child pBMI (*β*: –0.497, 95% CI: –1.503, 0.509) Food-secure households: child pBMI (*β*: 0.350, 95% CI: –0.440, 1.139)	[52]
Unhealthy food in community								
Longitudinal study	United States	From kindergarten (K) to 12th grade. The age range of the children in this study is ∼5–18 y old	106 schools	Environment type Healthier SFE: is characterized by a higher proportion of healthy options within the NSLP and in competitive foods (a la carte items and vending machines). This is measured by: NSLP Healthy Scale and Competitive Food Healthy Scale (90th percentile) Unhealthier SFE: is characterized by a lower proportion of healthy options in the NSLP and competitive foods (10th percentile) Low-density CFE: refers to an area around the school with a lower number or presence of certain types of food outlets within a 400-m roadway network. On the basis of the composite healthier profile, a low-density CFE is suggested by: number of limited-service restaurants: 0 (10th percentile). Number of convenience stores: 0 (10th percentile). Presence of small grocery stores: 1 (Yes). Presence of upgraded stores (healthy corner stores): 1 (Yes) High-density CFE: refers to an area around the school with a higher number or presence of certain types of food outlets within a 400-m roadway network. On the basis of the composite unhealthier profile, a high-density CFE is suggested by: number of limited-service restaurants: 8 (90th percentile). Number of convenience stores: 3 (90th percentile). Presence of small grocery stores: 0 (No). Presence of upgraded stores: 0 (No).	Obesity rate	Time, school level, % of students eligible for FRPM, % of Hispanic students, % of non-Hispanic Black students	Unhealthy SFE; low-density CFE: Obesity rate at baseline (*β*: –0.014, 95% CI: –0.067, 0.039, *P* = 0.611) Difference in obesity rate trend (*β*: 0.001, 95% CI: –0.005, 0.007, *P* = 0.674) Healthy SFE; high-density CFE: Obesity rate at baseline (*β*: 0.026, 95% CI: –0.006, 0.059, *P* = 0.115) Difference in obesity rate trend (*β*: –0.010, 95% CI: –0.022, 0.001, *P* = 0.080) Unhealthy SFE; high-density CFE: Obesity rate at baseline (*β*: –0.050, 95% CI: –0.096, –0.004, *P* = 0.033) ∗ Difference in obesity rate trend (*β*: 0.014, 95% CI: –0.022, 0.001, *P* = 0.080)	[49]
Case-control study	Iran	14–18 y old	406 adolescents (210 boys and 196 girls)	Out of home food environment (included questions about: the availability of unhealthy foods at school and out of home; distance to the nearest supermarket, fruit shop, and fast food restaurant; the percentage of the child's pocket money spent on foods out of home)	Normal weight: defined as a BMI-for-age-and-sex percentile below the 85th Overweight: defined as a BMI-for-age-and-sex percentile between the 85th and 95th Obese: defined as a BMI-for-age-and-sex percentile above the 95th	—	Overweight/obese: mean difference: 0.1, *P* = 0.7	[39]
Availability of food in retail food vendors								
Cross-sectional (individual data) and partially ecologic (environmental component)	Brazil	Between 6 and 59 mo (<5 y old)	665 children (360 boys and 305 girls)	Healthiness of the consumer's food environment (stratified from AUDITNOVA instrument scores)	Overweight: WHZ > +2	Individual variables (woman: age and education; child: age and sex) and socioeconomic (per capita household income)	Presence of overweight (low healthiness vs. high healthiness of the consumer's food environment): OR: 1.69, 95% CI: 1.05, 2.73, *P* = 0.03 ∗	[50]
Cross-sectional (individual data) and partially ecologic (environmental component)	Brazil	Between 6 and 59 mo (<5 y old)	665 children (360 boys and 305 girls)	Availability of ultraprocessed food in consumer's food environment	Overweight: WHZ > +2	Individual variables (woman: age and education; child: age and sex) and socioeconomic (per capita household income)	Presence of overweight (high availability vs. low availability of ultrapossessed food in consumer's food environment): OR: 2.64, 95% CI: 1.61, 4.33, *P* < 0.001 ∗	[50]
Cross-sectional study	Mexico	8–10 y old	218 children, 120 girls and 98 boys	Food access in convenience stores (250 m buffer)-shelf-space processed foods (m)	% BF Abdominal fat % BMI *z*-score	Sex, age, caretaker's educational level, physical activity, and proximity to school	% BF: *β*: 0.071, 95% CI: –0.249, 0.392, *P* = 0.662 Abdominal fat %: *β*: 0.047, 95% CI: –0.409, 0.502, *P* = 0.845 BMI *z*-score: *β*: 0.002, 95% CI: 0.000, 0.003, *P* = 0.008 ∗	[53]
Cross-sectional study	Mexico	8–10 y old	218 children, 120 girls and 98 boys	Food access in convenience stores (250 m buffer)-shelf-space non or minimally processed foods (m)	% BF Abdominal fat % BMI *z*-score	Sex, age, caretaker's educational level, physical activity, and proximity to school	% BF: *β*: 0.055, 95% CI: –0.105, 0.216, *P* = 0.497 Abdominal fat %: *β*: 0.049, 95% CI: –0.178, 0.277, *P* = 0.661 BMI *z*-score: *β*: 0.072, 95% CI: 0.009, 0.135, *P* = 0.025 ∗	[53]
Cross-sectional study	Mexico	8–10 y old	218 children, 120 girls and 98 boys	Food access in convenience stores (250 m buffer)-ratio processed foods/non or minimally processed foods (m)	% BF Abdominal fat % BMI *z*-score	Sex, age, caretaker's educational level, physical activity, and proximity to school	% BF: *β*: 0.210, 95% CI: –0.592, 0.479, *P* = 0.125 Abdominal fat %: *β*: 0.266, 95% CI: –0.121, 0.654, *P* = 0.174 BMI *z*-score: *β*: –0.012, 95% CI: –0.061, 0.037, *P* = 0.662	[53]
Longitudinal cohort study	United States	2–5 y old	3724 children (1882 boys and 1842 girls)	Healthy Food Availability Index (HFAI) score	BMI *z*-score	Age, sex, race/ethnicity, insurance, neighborhood SES	Mean BMI *z*-score (low HFAI score vs. no stores): *β*: 0.101, SE: 0.138, *P* = 0.462 Mean BMI *z*-score (moderate HFAI score vs. no stores): *β*: 0.115, SE: 0.137, *P* = 0.403 Mean BMI *z*-score (high HFAI score vs. no stores): *β*: 0.045, SE: 0.140, *P* = 0.751 Change in mean BMI *z*-score per month [low HFAI score vs. no stores × age (months)]: *β*: –0.001, SE: 0.003, *P* = 0.827 Change in mean BMI *z*-score per month [moderate HFAI score vs. no stores × age (months)]: *β*: –0.001, SE: 0.003, *P* = 0.785 Change in mean BMI *z*-score per month [high HFAI score vs. no stores × age (months)]: *β*: 0.000, SE: 0.003, *P* = 0.868	[54]
2A: Food accessibility								
Density of food outlets (categorized as healthy and unhealthy, or mixed)								
Density of healthy food outlets								
Cross-sectional study	Brazil	Aged 8 and 9 y old	366 children (177 boys and 189 girls)	Presence of healthy food stores (stores where the acquisition of fresh or minimally processed foods represents >50% of the total acquisition) (200 m buffer around schools)	Obesity: BMI cut-off points by age	Sex, per capita income, mean number of meals per day, and taking snacks to school	Obesity (have vs. not have healthy food stores within 200 m buffer around schools): OR: 0.37, 95% CI: 0.17, 0.82, *P* < 0.05 ∗	[55]
Cross-sectional study	Brazil	Aged 8 and 9 y old	366 children (177 boys and 189 girls)	Presence of healthy food stores (400 m buffer around schools)	Obesity: BMI cut-off points by age	Sex, per capita income, mean number of meals per day, and taking snacks to school	Obesity (have vs. not have healthy food stores within 400 m buffer around schools): OR: 0.63, 95% CI: 0.20, 1.99	[55]
Cross-sectional study	Brazil	Aged 8 and 9 y old	366 children (177 boys and 189 girls)	Presence of healthy food stores (200 m buffer around households)	Obesity: BMI cut-off points by age	Sex, per capita income, mean number of meals per day, and taking snacks to school	Obesity (have vs. not have healthy food stores within 200 m buffer around households): OR: 0.30, 95% CI: 0.11, 0.79, *P* < 0.05 ∗	[55]
Cross-sectional study	Brazil	Aged 8 and 9 y old	366 children (177 boys and 189 girls)	Presence of healthy food stores (400 m buffer around households)	Obesity: BMI cut-off points by age	Sex, per capita income, mean number of meals per day, and taking snacks to school	Obesity (have vs. not have healthy food stores within 400 m buffer around households): OR: 0.19, 95% CI: 0.06, 0.59, *P* < 0.05 ∗	[55]
Longitudinal study	United States	From kindergarten (K) to 12th grade. The age range of the children in this study is ∼5–18 y old	106 schools	Number of an upgraded store (participated in local healthy corner store initiatives) within a 400-m roadway network of each school	Obesity rate	Time, school level, % of students eligible for FRPM, % of Hispanic students, % of non-Hispanic Black students	Obesity rate at baseline: *β*: –0.035, 95% CI: –0.053, –0.017, *P* < 0.001 Change in obesity rate over time: *β*: 0.004, 95% CI: –0.002, 0.009, *P* = 0.220	[49]
Cross-sectional study	Norway	Seventh-graders in primary schools in Oslo (around 12–13 y old)	802 adolescents (369 boys and 433 girls)	Number of "healthy" food outlets within an 800 m radius buffer around the residential neighborhood address of the participant	Overweight: was defined by applying the age-and sex-specific IOTF BMI (kg/m^2^) cut-offs	Ethnicity of the participants, parental education, and neighborhood deprivation level	Overweight: OR: 1.00, 95% CI: 0.97, 1.02	[56]
Density of unhealthy food outlets								
Longitudinal cohort study	United Kingdom	Children aged 4–5 y (reception year); Children aged 10–11 y (year 6)	14,084 children at ages 4–5 and 5637 children at ages 10–11	Relative density of unhealthy food outlets (include bakeries, confectioners, convenience stores, and takeaways)	Overweight or obesity: BMI ≥85th centile for sex and age	Maternal BMI and smoking in early pregnancy, educational attainment, ethnicity and parity, clustering of observations within areas, supermarket density	Lower super output area (LSOA) (mean populations of 1500 and an area of 4 km^2^): overweight or obesity at ages 4–5 (RR: 1.000, 95% CI: 0.993, 1.007); at ages 10–11 (RR: 1.009, 95% CI: 0.998, 1.020) Middle-level super output areas (MSOA) (mean populations of 7000 and an area of 21 km^2^): overweight or obesity at ages 4–5 (RR: 0.997, 95% CI: 0.987, 1.008); at ages 10–11 (RR: 1.021, 95% CI: 1.005, 1.037) ∗	[57]
Cross-sectional study	Brazil	Aged 8 and 9 y old	366 children (177 boys and 189 girls)	Presence of unhealthy food stores (200 m buffer around households)	Obesity: BMI cut-off points by age	Sex, per capita income, mean number of meals per day, and taking snacks to school	Obesity (have vs. not have unhealthy food stores within 200 m buffer around households): OR: 1.32, 95% CI: 0.23, 7.76	[55]
Cross-sectional study	Brazil	Aged 8 and 9 y old	366 children (177 boys and 189 girls)	Presence of unhealthy food stores (stores where the acquisition of ultraprocessed foods represents >50% of the total purchase) (200 m buffer around schools)	Obesity: BMI cut-off points by age	Sex, per capita income, mean number of meals per day, and taking snacks to school	Obesity (have vs. not have unhealthy food stores within 200 m buffer around schools): OR: 3.33, 95% CI: 1.01, 11.02	[55]
Cross-sectional Study	United States	5–9 y old	1296 families with children	Living in a food desert	Child pBMI	Area deprivation index, online shopping experience, shopping by car, number of adults, number of children, child's gender, primary caregiver's education, public assistance receipt, household income, race/ethnicity, eating American foods at home and time in the United States	Food-insecure households: child pBMI (live vs. not live in a food desert): *β*: 5.723, 95% CI: 0.560, 10.887, *P* < 0.05 ∗ Food-secure households: child pBMI (live vs. not live in a food desert): *β*: –3.898, 95% CI: –8.803, 1.008	[52]
Cross-sectional study	Norway	Seventh-graders in primary schools in Oslo (around 12–13 y old)	802 adolescents, 369 boys and 433 girls	Number of "unhealthy" food outlets within an 800 m radius buffer around the residential neighborhood address of the participant	Overweight: was defined by applying the age-and sex-specific IOTF BMI (kg/m^2^) cut-offs	Ethnicity of the participants, parental education, and neighborhood deprivation level	Overweight: OR: 1.03, 95% CI: 0.96, 1.09	[56]
Cross-sectional study	United States	3–15 y old	627 children	Number of unhealthy outlets within 0.25 mile	BMI *z*-score	Active commuting, race/ethnicity, income, >200% federal poverty level, presence of small grocery store w/in 0.25 miles, presence of supermarket w/in 0.25 miles	BMI z-score: *β*: 0.05, *P* = 0.04 ∗ Among active commuters: BMI *z*-score (*β*: 0.10, *P* < 0.001) ∗ Among nonactive commuters: BMI *z*-score (*β*: –0.01, *P* = 0.83)	[58]
Cross-sectional study	Brazil	9 and 10 y old	717 students, 368 girls and 349 boys	Index of establishments that predominantly sell ultraprocessed food (%): (establishments that predominantly sell ultraprocessed food/total number of food shops) × 100	Obesity: BMI-per-age higher than *z* score + 2	Gender, time staying in school, monthly family income per capita, and mean contextual income	Obesity (quartile 4 vs. quartile 1 of the index of establishments that predominantly sell ultraprocessed food): OR: 1.33, 95% CI: 0.63, 2.82	[59]
Cross-sectional study	Canada	Grade 5 students, mostly 10–11 y old	812 children (432 girls and 380 boys)	Proportion of choose least often food outlets relative to all	BMI *z*-score	Gender, total energy intake (or physical activity for models examining BMI or weight status), parental education, household income, and area-level material deprivation	BMI *z*-score: *β*: 0.15, 95% CI: –0.0064, 0.30, *P* = 0.060	[60]
Cross-sectional study	Canada	Grade 5 students, mostly 10–11 y old	812 children (432 girls and 380 boys)	Absolute number of choose least often food outlets	BMI *z*-score	Gender, total energy intake (or physical activity for models examining BMI or weight status), parental education, household income and area-level material deprivation	BMI *z*-score: *β*: 0.058, 95% CI: 0.0015, 0.12, *P* = 0.044 ∗	[60]
Cross-sectional study	Spain	9–12 y old	2213 children, 1060 boys and 1153 girls	Unhealthy food environment, unhealthy food outlets (17.7 facilities/km^2^) (included petrol/gasoline station, bar or pub, coffee shop, restaurant, convenience store, shopping malls)	BMI *z*-score Overweight/obesity status: BMI *z*-score higher than +1 Waist circumference *z*-score % BF *z*-score	Study design, maternal and paternal education, paternal occupation, occupation, and parental country of birth, maternal household economy, maternal smoking status, maternal BMI, the number of siblings, and area-level SES	BMI *z*-score: *β*: 0.04, 95% CI: –0.04, 0.11 Overweight/obesity status: OR: 1.11, 95% CI: 0.97, 1.28 Waist circumference *z*-score: *β*: 0.04, 95% CI: –0.02, 0.10 % BF *z*-score: *β*: 0.04, 95% CI: –0.02, 0.10	[61]
Ecologic study	United Kingdom	10–11 y old	6791 MSOAs covering England	Obesogenic Environment Score	Overweight Obesity	Income deprivation, White ethnicity, rurality index, crime deprivation	Obesogenicity first measurement model: Obesity (*β*: 0.069, 95% CI: 0.062, 0.076) ∗ All overweight (*β*: 0.056, 95% CI: 0.050, 0.064) ∗ Obesogenicity second measurement model: Obesity (*β*: 0.071, 95% CI: 0.064, 0.079) ∗ All overweight (*β*: 0.065, 95% CI: 0.054, 0.075) ∗	[62]
Cross-sectional study	Brazil	8 and 9 y old	367 children (190 girls and 177 boys)	Obesogenic environment (includes predominantly ultraprocessed food stores, traffic accidents, crime, and walkability)	% BF	Child's sex and SES	% BF: *β*: 0.19, SE: 0.08, *P* = 0.02 ∗	[63]
Density of mixed food outlets								
Cross-sectional study	Brazil	Aged 8 and 9 y	366 children, 177 boys and 189 girls	Presence of mixed food stores (stores where there is a predominance of acquisition of culinary preparations or processed foods, or where there is no predominance of acquisition of fresh/minimally processed foods or ultraprocessed foods) (200 m buffer around schools)	Obesity: BMI cut-off points by age	Sex, per capita income, mean number of meals per day, and taking snacks to school	Obesity (have vs. not have mixed food stores within 200 m buffer around schools): OR: 0.84, 95% CI: 0.38, 1.88	[55]
Cross-sectional study	Brazil	Aged 8 and 9 y	366 children, 177 boys and 189 girls	Presence of mixed food stores (400 m buffer around schools)	Obesity: BMI cut-off points by age	Sex, per capita income, mean number of meals per day, and taking snacks to school	Obesity (have vs. not have mixed food stores within 400 m buffer around schools): OR: 1.19, 95% CI: 0.42, 3.41	[55]
Cross-sectional study	Brazil	Aged 8 and 9 y	366 children, 177 boys and 189 girls	Presence of mixed food stores (200 m buffer around households)	Obesity: BMI cut-off points by age	Sex, per capita income, mean number of meals per day, and taking snacks to school	Obesity (have vs. not have mixed food stores within 200 m buffer around households): OR: 0.42, 95% CI: 0.17, 1.08	[55]
Cross-sectional study	Brazil	Aged 8 and 9 y	366 children, 177 boys and 189 girls	Presence of mixed food stores (400 m buffer around households)	Obesity: BMI cut-off points by age	Sex, per capita income, mean number of meals per day, and taking snacks to school	Obesity (have vs. not have mixed food stores within 400 m buffer around households): OR: 0.67, 95% CI: 0.17, 1.08	[55]
Cross-sectional study	United States	10–14 y old	335 children, 145 boys and 190 girls	Store count within 0.5 mile of pharmacy, discount stores, and variety stores	BMI *z*-score	Children's gender, caregivers' educational attainment, household income to federal poverty threshold, and household Supplemental Nutrition Assistance Program (SNAP) and Special Supplemental Nutrition Program for WIC participation	Using Extensively Revised Combined database (*β*: 0.032, 95% CI: –0.046, 0.113, *P* = 0.430) RefUSA – 2 NAICS (*β*: 0.071, 95% CI: –0.010, 0.155, *P* = 0.092) SNAP (*β*: 0.022, 95% CI: –0.129, 0.141, *P* = 0.753)	[64]
Cross-sectional study	United States	10–14 y old	335 children, 145 boys and 190 girls	Store count within 1 mile of pharmacy, discount stores, and variety stores	BMI *z*-score	Children's gender, caregivers' educational attainment, household income to federal poverty threshold, and household SNAP and Special Supplemental Nutrition Program for WIC participation	Using Extensively Revised Combined database (*β*: 0.053, 95% CI: 0.001, 0.108, *P* = 0.053) RefUSA – 2 NAICS (*β*: 0.069, 95% CI: 0.007, 0.121, *P* = 0.021) ∗ SNAP (*β*: 0.080, 95% CI: –0.004, 0.149, *P* = 0.175) CLF (*β*: 0.101, 95% CI: –0.008, 0.179, *P* = 0.021) ∗	[64]
Cross-sectional study	United States	10–14 y old	335 children, 145 boys and 190 girls	Store count within 1 mile of supermarket, wholesale, club stores, and large grocery stores	BMI *z*-score	Children's gender, caregivers' educational attainment, household income to federal poverty threshold, and household SNAP and Special Supplemental Nutrition Program for WIC participation	Using Extensively Revised Combined database (*β*: 0.004, 95% CI: –0.099, 0.137, *P* = 0.968) RefUSA – 2 NAICS (*β*: 0.075, 95% CI: 0.002, 0.145, *P* = 0.044) ∗ SNAP (*β*: 0.089, 95% CI: 0.009, 0.176, *P* = 0.031) ∗	[64]
Cross-sectional study	United States	10–14 y old	335 children, 145 boys and 190 girls	Store count within 0.25 mile of corner, convenience, small and medium grocery stores, and gas station stores	BMI *z*-score	Children's gender, caregivers' educational attainment, household income to federal poverty threshold, and household SNAP and Special Supplemental Nutrition Program for WIC participation	Using Extensively Revised Combined database (*β*: 0.025, 95% CI: –0.014, 0.055, *P* = 0.162) RefUSA – 2 NAICS (*β*: 0.018, 95% CI: –0.040, 0.067, *P* = 0.508) SNAP (*β*: 0.022, 95% CI: –0.015, 0.060, *P* = 0.257) CLF (*β*: 0.031, 95% CI: –0.023, 0.083, *P* = 0.246)	[64]
Cross-sectional study	United States	10–14 y old	335 children, 145 boys and 190 girls	Store count within 0.5 mile of corner, convenience, small and medium grocery stores, and gas station stores	BMI *z*-score	Children's gender, caregivers' educational attainment, household income to federal poverty threshold, and household SNAP and Special Supplemental Nutrition Program for WIC participation	Using Extensively Revised Combined database (*β*: 0.015, 95% CI: 0.004, 0.026, *P* = 0.006) ∗ RefUSA – 2 NAICS (*β*: 0.015, 95% CI: –0.004, 0.031, *P* = 0.081) SNAP (*β*: 0.014, 95% CI: –0.005, 0.027, *P* = 0.087) CLF (*β*: 0.015, 95% CI: –0.012, 0.036, *P* = 0.224)	[64]
Cross-sectional study	United States	10–14 y old	335 children, 145 boys and 190 girls	Store count within 1 mile of corner, convenience, small and medium grocery stores, and gas station stores	BMI *z*-score	Children's gender, caregivers' educational attainment, household income to federal poverty threshold, and household SNAP and Special Supplemental Nutrition Program for WIC participation	Using Extensively Revised Combined database (*β*: 0.005, 95% CI: 0.000, 0.009, *P* = 0.027) ∗ RefUSA – 2 NAICS (*β*: 0.005, 95% CI: –0.001, 0.010, *P* = 0.092) SNAP (*β*: 0.004, 95% CI: 0.000, 0.008, *P* = 0.039) CLF (*β*: 0.007, 95% CI: –0.005, 0.015, *P* = 0.143)	[64]
Cross-sectional study	United States	10–14 y old	335 children, 145 boys and 190 girls	Store count within 0.25 miles of full and limited-service restaurants and carryouts	BMI *z*-score	Children's gender, caregivers' educational attainment, household income to federal poverty threshold, and household SNAP and Special Supplemental Nutrition Program for WIC participation	Using Extensively Revised Combined database (*β*: 0.041, 95% CI: –0.013, 0.097, *P* = 0.145) RefUSA – 2 NAICS (*β*: 0.046, 95% CI: –0.003, 0.103, *P* = 0.093) SNAP (*β*: 0.017, 95% CI: –0.103, 0.141, *P* = 0.786)	[64]
Cross-sectional study	United States	10–14 y old	335 children, 145 boys and 190 girls	Store count within 0.5 miles of full and limited-service restaurants and carryouts	BMI *z*-score	Children's gender, caregivers' educational attainment, household income to federal poverty threshold, and household SNAP and Special Supplemental Nutrition Program for WIC participation	Using Extensively Revised Combined database (*β*: 0.010, 95% CI: –0.002, 0.021, *P* = 0.108) RefUSA – 2 NAICS (*β*: 0.020, 95% CI: 0.003, 0.033, *P* = 0.009) SNAP (*β*: 0.058, 95% CI: –0.019, 0.141, *P* = 0.225)	[64]
Cross-sectional study	United States	10–14 y old	335 children, 145 boys and 190 girls	Store count within 1 mile of full and limited-service restaurants and carryouts	BMI *z*-score	Children's gender, caregivers' educational attainment, household income to federal poverty threshold, and household SNAP and Special Supplemental Nutrition Program for WIC participation	Using Extensively Revised Combined database (*β*: 0.000, 95% CI: –0.002, 0.003, *P* = 0.764) RefUSA – 2 NAICS (*β*: 0.002, 95% CI: –0.001, 0.007, *P* = 0.206) SNAP (*β*: 0.013, 95% CI: –0.008, 0.028, *P* = 0.158)	[64]
Cross-sectional analysis of cohort baseline data	United States	Mean 16 y old, ranging from 14, 20 y old, with 98% between 15 and 17 y old	2263 children, 1035 boys, and 1228 girls	All food outlets (fast food outlets, full-service restaurants, convenience stores, grocery stores/supermarkets)	BMI	Race/ethnicity, parent education, family affluence, neighborhood land use mix, neighborhood population density, and neighborhood poverty rate	1 km SFE: BMI: Combined with fruit and vegetable (*β*: 0.01, 95% CI: –0.07, 0.10) Combined with snacks (*β*: –0.001, 95% CI: –0.03, 0.03) Combined with soda (*β*: –0.002, 95% CI: –0.03, 0.02) 5 km SFE: BMI: Combined with fruit and vegetable (*β*: 0.002, 95% CI: –0.23, 0.53) Combined with snacks (*β*: 0.0002, 95% CI: –0.002, 0.002) Combined with soda (*β*: –0.0001, 95% CI: –0.002, 0.002)	[47]
Cross-sectional study	Mexico	12–19 y old	2239 adolescents (1063 males and 1176 females)	Density of all stores [quotient of the number of stores over the number of inhabitants within the municipality (per 10,000 inhabitants)]	Overweight: *z*-scores ≥1 SD and ≤ 2 SD Obesity: *z*-scores >2 SD	Sex and SES	Overweight or obesity: *β*: 0.007, 95% CI: 0.002, 0.012, *P* = 0.006 ∗	[65]
Cross-sectional study	Brazil	12–19 y old	504 adolescents (259 males and 245 females)	Presence of markets, supermarkets, and grocery stores within a 500 m buffer zone of the adolescents' homes	Overweight/obese: ≥ percentile 85th	Sex, census tract, race, neighborhood violence, y of residence, physical activity and the intra-municipal Human Development Index (HDI)	Overweight/obese (have vs. not have markets, supermarkets and grocery stores within a 500 m buffer zone): OR: 1.45, 95% CI: 0.74, 2.82, *P* = 0.28	[66]
Cross-sectional study	Brazil	Adolescents in the age group of 12–17 y	2530 adolescents (1273 boys and 1257 girls)	Number of ready-to-eat food shops (dinners, snack shops, bars, restaurants, supermarkets, hypermarkets, and grocery stores) within the 800 m buffer around the school	Obesity: weight and height measurements above the score-z + 2	Sex, age, socioeconomic score, lives with the parents, how often the legal guardian has supper with the adolescent, breakfast-having frequency, vulnerability to health index, managerial dependence, number of operational drinking fountains	Obesity (2nd tercile vs. 1st tercile of the number of ready-to-eat food shops within the 800 m buffer around the school): OR: 1.24, 95% CI: 1.06, 1.44, *P* < 0.05 ∗ Obesity (3rd tercile vs. 1st tercile of the number of ready-to-eat food shops within the 800 m buffer around the school): OR: 1.44, 95% CI: 1.17, 1.77, *P* < 0.05 ∗	[48]
Cross-sectional study	United States	Elementary school students in grades K-6	10,327 children	Modified Retail Food Environment Index [mRFEI (%)]	Overweight or obese	Age, sex, percent free and reduced meal students	Overweight or obese: OR: 1.00, 95% CI: 1.00, 1.01	[67]
Cross-sectional study	United Kingdom	4–11 y old	728 children (353 boys and 375 girls)	Neighborhood food environment: using Ordnance Survey Point of Interest data, which specifies the physical location of different types of food outlets	BMI-SDS	Child age, child sex, and child ethnicity, and for clustering at the school level	Child BMI-SDS: *β*= –0.09, 95% CI: –0.34, 0.17, *P* = 0.443	[68]
Cross-sectional study	China	8–16.5 y old	4970 children (2619 boys and 2351 girls)	Neighborhood food environment (Assessed based on the number of fast food restaurants, Chinese-style restaurants, fruit and vegetable stores, supermarkets/convenience stores, and milk tea shops/bakeries/dessert shops. The neighborhood was defined as being within the scope of 500 m from the resident's home)	BMI	—	Children's BMI: path coefficients >0, *P* < 0.05 ∗	[69]
Density of fast food outlets								
Cross-sectional study	United Kingdom	Aged 4 or 5 y, mean age 5.0 y (SD 0.4 y)	129,893 children (66,260 boys and 63,633 girls)	Fast food density per 100,000 population	Obesity: ≥95th centile for age and sex	Low birth weight, breast-feeding at birth, fruit and vegetable consumption and physical activity	Obesity: OR: 1.002, 95% CI: 1.001, 1.004, *P* < 0.001 ∗	[43]
Longitudinal study	United States	Around 5–6 y old (kindergarten) to 17–18 y old (twelfth grade)	Fourth to sixth grade: 33,308 children (16,871 boys and 16,437 girls) Fourth to eighth grade: 10,597 children (5457 boys and 5140 girls) Sixth to eighth grade: 33,130 children (17,083 boys and 16,047 girls) Eighth to tenth grade: 34,758 children (18,005 boys and 16,753 girls)	Change in fast food exposure (number of fast food restaurants located along the shortest-distance route between home and school)	BMI *z*-score	Median household income, per cent population living in poverty, median gross rent, and educational attainment for Census block group in which student resides, school district fixed effects also included	Fourth to sixth grade: *β*: –0.001, SE: 0.001 Fourth to eighth grade: *β*: –0.004, SE: 0.010 Sixth to eighth grade: *β*: 0.001, SE: 0.004 Eighth to tenth grade: *β*: 0.001, SE: 0.001	[70]
Ecologic Study	United States	—	3140 children	Number of fast-food restaurants (per 1000 county residents)	Overweight/obesity rates	—	Overweight/obesity rate: *β*: –0.0003, *P* < 0.001 at the national level ∗	[71]
Cross-sectional study	Japan	Fifth- to ninth-grade children (around 10–15 y old)	7277 children (3787 boys and 3490 girls)	Density of fast food restaurants in each school district	Obesity: based on the percent Overweight, ≥20% was considered obese, whereas <20% was considered non-obese	Age, gender, school district area, population density, and physical activity environment	Obesity: OR: 0.640, 95% CI: 0.274, 1.494	[72]
Cross-sectional study	Belgium	Children and adolescents aged 2.5–18 y	3404 primary schools and 1195 secondary schools	Density of fast food outlets within 500 m	Overweight: according to IOTF	Sex, level of urbanicity of municipality and percentage of pupils with a low educated mother	School year: 2014–2015 % overweight: Younger than 6 y (*β*: 0.100, SE: 0.033, *P* = 0.003) ∗ 6–12 y old (*β*: 0.080, SE: 0.026, *P* = 0.002) ∗ 13–14 y (*β*: 0.050, SE: 0.048, *P* = 0.305) ∗ 15–18 y (*β*: –0.111, SE: 0.075, *P* = 0.138) ∗	[73]
Cross-sectional study	Belgium	Children and adolescents aged 2.5–18 y	3404 primary schools and 1195 secondary schools	Density of fast food outlets within 1000 m	Overweight: according to IOTF	Sex, level of urbanicity of municipality and percentage of pupils with a low educated mother	School year: 2014–2015 % overweight: Younger than 6 y (*β*: 0.053, SE: 0.010, *P* < 0.001) ∗ 6–12 y old (*β*: 0.046, SE: 0.008, *P* < 0.001) ∗ 13–14 y (*β*: 0.030, SE: 0.016, *P* = 0.054) 15–18 y (*β*: –0.006, SE: 0.024, *P* = 0.806)	[73]
Longitudinal cohort study	United Kingdom	Main analysis based at age 16, robustness analyses at age 10	3585 children (1864 girls and 1721 boys)	Closest fast food within 1 mile	BMI	Gender, highest parental social class, land of residence, residing in London, residing in an urban area, ethnicity and birth weight, mother's BMI in 1980, adolescent's smoking status and household ownership of microwave	BMI: *β*: –0.1739, SE: 0.158, *P* = 0.392	[74]
Longitudinal cohort study	United Kingdom	Main analysis based at age 16, robustness analyses at age 10	3585 children (1864 girls and 1721 boys)	Closest fast food within 1–2 miles	BMI	Gender, highest parental social class, land of residence, residing in London, residing in an urban area, ethnicity and birth weight, mother's BMI in 1980, adolescent's smoking status and household ownership of microwave	BMI: *β*: –0.1103, SE: 0.170, *P* = 0.518	[74]
Longitudinal cohort study	United Kingdom	Main analysis based at age 16, robustness analyses at age 10	3585 children (1864 girls and 1721 boys)	Closest fast food within 2–3 miles	BMI	Gender, highest parental social class, land of residence, residing in London, residing in an urban area, ethnicity and birth weight, mother's BMI in 1980, adolescent's smoking status, and household ownership of microwave	BMI: *β*: 0.1160, SE: 0.191, *P* = 0.543	[74]
Longitudinal cohort study	United Kingdom	Main analysis based at age 16, robustness analyses at age 10	3585 children (1864 girls and 1721 boys)	Closest fast food within 3–5 miles	BMI	Gender, highest parental social class, land of residence, residing in London, residing in an urban area, ethnicity and birth weight, mother's BMI in 1980, adolescent's smoking status and household ownership of microwave	BMI: *β*: –0.2408, SE: 0.153, *P* = 0.120	[74]
Longitudinal cohort study	United Kingdom	Main analysis based at age 16, robustness analyses at age 10	3585 children (1864 girls and 1721 boys)	Exposure intensity (sum of durations per outlet/distance) of fast food	BMI	Gender, highest parental social class, land of residence, residing in London, residing in an urban area, ethnicity and birth weight, mother's BMI in 1980, adolescent's smoking status and household ownership of microwave	BMI: *β*: 0.002, SE: 0.006, *P* = 0.710	[74]
Ecologic study	United States	Aged 5, 19 y old, grades K-12	2064 census tracts	Percentage of fast food restaurants	Hotspots of childhood obesity	Percentage children, percentage female, percentage non-Hispanic Black, percentage Hispanic, median household income, percentage convenience stores	Hotspots of childhood obesity: *β*: 3.05, 95% CI: 0.14, 5.97, *P* = 0.012 ∗	[75]
Longitudinal study	United States	From kindergarten (K) to 12th grade. The age range of the children in this study is ∼5–18 y old	106 schools	Number of limited-service restaurants (i.e., fast food restaurants) within a 400-m roadway network of each school	Obesity rate	Time, school level, percentage of students eligible for FRPM, percentage of Hispanic students, percentage of non-Hispanic Black students	Obesity rate at baseline: *β*: 0.002, 95% CI: 0.001, 0.004, *P* = 0.001 ∗ Change in obesity rate over time: *β*: 0.000, 95% CI: –0.001, 0.000, *P* = 0.461	[49]
Cross-sectional analysis of cohort data	Portugal	7-y-old	5203 children (2686 boys and 2517 girls)	Number of fast food restaurants located within a 400-m walkable distance from the child's residence	Obesity: BMI *z*-score >2 SD	Maternal education, socioeconomic deprivation (score), dwelling density (dwellings/ha), proportion of mixed-use buildings (%), street connectivity (intersections/ha), pedestrian access to destinations (number within 400 m), pedestrian access to sport facilities (number within 400 m), pedestrian access to urban green spaces (number within 400 m), pedestrian access to of fast food restaurants (number within 400 m), normalized difference vegetation index	Obesity: OR: 1.37, 95% CI: 1.06, 1.77 ∗	[76]
Cross-sectional study	Brazil	12–19 y old	504 adolescents (259 males and 245 females)	Presence of fast food restaurants within a 500 m buffer zone of the adolescents' homes	Overweight/obese: ≥ percentile 85th	Sex, census tract, race, neighborhood violence, years of residence, physical activity and the intra-municipal HDI	Overweight/obese (have vs. not have fast food restaurants within a 500 m buffer zone): OR: 2.53, 95% CI: 1.02, 6.27, *P* = 0.04 ∗	[66]
Longitudinal cohort study	United States	2–5 y old	3724 children (1882 boys and 1842 girls)	Density of fast food outlets in a child's neighborhood	BMI *z*-score	Age, sex, race/ethnicity, insurance, neighborhood SES	Mean BMI *z*-score (high density of fast food outlets in a child's neighborhood vs. no stores): *β*: –0.122, SE: 0.118, *P* = 0.301 Mean BMI *z*-score (medium density of fast food outlets in a child's neighborhood vs. no stores): *β*: – 0.081, SE: 0.117, *P* = 0. 486 Mean BMI *z*-score (low density of fast food outlets in a child's neighborhood vs. no stores): *β*: –0.197, SE: 0.121, *P* = 0.104 Change in mean BMI *z*-score per month [high density of fast food outlets in a child's neighborhood vs. no stores × age (months)]: *β*: 0.004, SE: 0.002, *P* = 0.129 Change in mean BMI *z*-score per month [medium density of fast food outlets in a child's neighborhood vs. no stores × age (months)]: *β*: 0.003, SE: 0.003, *P* = 0.215 Change in mean BMI *z*-score per month [low density of fast food outlets in a child's neighborhood vs. no stores × age (months)]: *β*: 0.006, SE: 0.003, *P* = 0.024 ∗	[54]
Cross-sectional study	Norway	Seventh-graders in primary schools in Oslo (around 12–13 y old)	802 adolescents (369 boys and 433 girls)	Number of fast food restaurants within an 800 m radius buffer around the residential neighborhood address of the participant	Overweight: was defined by applying the age-and sex-specific IOTF BMI (kg/m^2^) cut-offs	Ethnicity of the participants, parental education, and neighborhood deprivation level	Overweight: OR: 0.96, 95% CI: 0.84, 1.09	[56]
Longitudinal study	United States	High school students in grades 9–12	361,942 students	Fast food restaurant (<0.25 miles)	Adolescent obesity	Race/ethnicity, gender, eligibility for free or reduced-price lunch, native born, special education, speaking English at home, age, grade, the building type of their home, and the number of total food outlet within 10 blocks	Adolescent obesity: *β*: –0.013, SE: 0.001, predicted likelihood: 0.152, 95% CI: 0.150, 0.153 ∗	[77]
Longitudinal study	United States	High school students in grades 9–12	361,942 students	Fast food restaurant (0.25–0.50 miles)	Adolescent obesity	Race/ethnicity, gender, eligibility for free or reduced-price lunch, native born, special education, speaking English at home, age, grade, the building type of their home, and the number of total food outlet within 10 blocks	Adolescent obesity: *β*: –0.025, SE: 0.002, predicted likelihood: 0.153, 95% CI: 0.146, 0.160 ∗	[77]
Longitudinal cohort study	Netherlands	Ages of 4 and 14 y old. The researchers included objectively determined measures at the age of 4, 6, 10 and 14 y	4235 children (2057 boys and 2178 girls)	Change in relative fast food exposure	BMI FMI FFMI	The time between measurements	+10%-point: BMI: Lower maternal education level (*β*: 0.01, 95% CI: –0.01, 0.04) Higher maternal education level (*β*: 0.01, 95% CI: –0.01, 0.02) FMI: Lower maternal education level (*β*: 0.04, 95% CI: 0.00, 0.08, *P* < 0.05) ∗ Higher maternal education level (*β*: 0.01, 95% CI: –0.01, 0.04) FFMI: Lower maternal education level (*β*: 0.00, 95% CI: –0.02, 0.03) Higher maternal education level (*β*: 0.01, 95% CI: –0.01, 0.03) +1 outlet: BMI: Lower maternal education level (*β*: 0.01, 95% CI: –0.01, 0.02) Higher maternal education level (*β*: 0.01, 95% CI: –0.01, 0.02) FMI: Lower maternal education level (*β*: 0.02, 95% CI: –0.00, 0.04) Higher maternal education level (*β*: 0.01, 95% CI: –0.01, 0.04) FFMI: Lower maternal education level (*β*: 0.01, 95% CI: –0.01, 0.02) Higher maternal education level (*β*: –0.00, 95% CI: –0.02, 0.01)	[78]
Longitudinal cohort study	United Kingdom	Aged 11/12 (wave 5 in 2011/2012) and 14/15 (wave 6 in 2014/2015)	9736 children (4808 boys and 4928 girls)	Count of fast food outlets in Local Authority District	Overweight: defined using age and sex-specific IOTF cut points	—	Overweight at age 11/12: OR: 1.0006, 95% CI: 1.0002, 1.0009 ∗ Overweight at age 14/15: OR: 1.0005, 95% CI: 1.0002, 1.0008 ∗	[79]
Longitudinal cohort study	United Kingdom	Aged 11/12 (wave 5 in 2011/2012) and 14/15 (wave 6 in 2014/2015)	9736 children (4808 boys and 4928 girls)	Change in fast food outlets	Overweight: defined using age and sex-specific IOTF cut points	Neighborhood deprivation	LSOA between waves: overweight at age 14/15 (OR: 0.967, 95% CI: 0.929, 1.007, *P* = 0.105) ∗ Local authority district (LAD) between waves: overweight at age 14/15 (OR: 1.0001, 95% CI: 0.999, 1.002, *P* = 0.903)	[79]
Longitudinal study	China	Aged 2–18 y old	3244 children	Fast food restaurants presence (main results were within 3 km, other distance also done)	Overweight/obese: IOTF BMI cut-offs	Equivalent household income, number of children in the household, whether the child has entered puberty, between 2 and 5 y of age, whether ≥1 grandparent is living with the child, the number of households, population density, number of schools (primary and secondary), and price of pork and vegetables	Within 2 km: overweight/obese (have vs. not have fast food restaurants within 2 km): *β*: 0.056, SE: 0.032, *P* < 0.1 Within 2.5 km: overweight/obese (have vs. not have fast food restaurants within 2.5 km): *β*: 0.051, SE: 0.028, *P* < 0.1 Within 3 km: overweight/obese (have vs. not have fast food restaurants within 3 km): *β*: 0.063, SE: 0.027, *P* < 0.05 ∗ Within 4 km: overweight/obese (have vs. not have fast food restaurants within 4 km): *β*: 0.063, SE: 0.023, *P* < 0.01 ∗ Within 5 km: overweight/obese (have vs. not have fast food restaurants within 5 km): *β*: 0.025, SE: 0.034 ∗ BMI *z*-score (have vs. not have fast food restaurants among full range): *β*: 0.156, SE: 0.079, *P* < 0.1 BMI *z*-score if overweight (have vs. not have fast food restaurants among full range): *β*: 0.052, SE: 0.027, *P* < 0.1 For children with income ≥ median: overweight/obese (have vs. not have fast food restaurants among full range): *β*: 0.055, SE: 0.033, *P* < 0.1 For children with income < median: overweight/obese (have vs. not have fast food restaurants among full range): *β*: –0.060, SE: 0.052 For children aged <11: overweight/obese (have vs. not have fast food restaurants among full range): *β*: 0.087, SE: 0.046, *P* < 0.1 For children aged ≥11: overweight/obese (have vs. not have fast food restaurants among full range): *β*: 0.006, SE: 0.025 For boys: overweight/obese (have vs. not have fast food restaurants among full range): *β*: 0.017, SE: 0.030 For girls: overweight/obese (have vs. not have fast food restaurants among full range): *β*: 0.078, SE: 0.028, *P* < 0.01 ∗	[80]
Cross-sectional study	Netherlands	Primary-school-aged children	135 primary schools	Number of fast food outlets within 400 m (Euclidean distance) from a school as a proportion of the total amount of food retailers in this area	Overweight prevalence in the subdistrict	Neighborhood disadvantage level around schools (based on the schools' disadvantage scores)	All fast food outlets (*β*: 1.16, 95% CI: 0.46, 1.86) ∗ Fast food restaurants (*β*: 1.54, 95% CI: 0.76, 2.33) ∗ Grillrooms & kebab shops (*β*: 2.15, 95% CI: 1.15, 3.15) ∗ Takeaway restaurants (*β*: 3.69, 95% CI: 0.67, 6.71) ∗	[81]
Cross-sectional study	Netherlands	Primary-school-aged children	135 primary schools	Number of fast food outlets within 1000 m (Euclidean distance) from a school as a proportion of the total amount of food retailers in this area	Overweight prevalence in the subdistrict	Neighborhood disadvantage level around schools (based on the schools' disadvantage scores)	All fast food outlets (*β*: 0.46, 95% CI: 0.10, 0.82) ∗ Fast food restaurants (*β*: 0.45, 95% CI: 0.11, 0.78) ∗ Grillrooms and kebab shops (*β*: 2.01, 95% CI: 1.13, 2.89) ∗ Takeaway restaurants (*β*: –0.18, 95% CI: –0.36, 0.005)	[81]
Retrospective time-trend study	China	6–15 y old	7350 children	Density of Chinese fast food	Overweight: defined as sex-age-specific BMI >85th percentile of the WS/T586–2018 Obesity: defined as sex-age-specific BMI >95th percentile of the WS/T586–2018	Age, gender, neighborhood SES, urbanicity, a random effect for the 3 waves	Overweight/obesity Overall (OR: 0.68, 95% CI: 0.59, 0.78, *P* < 0.001) ∗ Wave 1 (OR: 0.57, 95% CI: 0.47, 0.70, *P* < 0.001) ∗ Wave 2 (OR: 0.55, 95% CI: 0.40, 0.76, *P* < 0.001) ∗ Wave 3 (OR: 0.89, 95% CI: 0.58, 1.36, *P* = 0.586) Primary school overall (OR: 0.68, 95% CI: 0.58, 0.80, *P* < 0.001) ∗ Middle school overall (OR: 0.67, 95% CI: 0.51, 0.88, *P* = 0.005) ∗ Obesity Overall (OR: 0.67, 95% CI: 0.55, 0.83, *P* < 0.001) ∗ Wave 1 (OR: 0.47, 95% CI: 0.33, 0.66, *P* < 0.001) ∗ Wave 2 (OR: 0.54, 95% CI: 0.34, 0.84, *P* = 0.006) ∗ Wave 3 (OR: 1.11, 95% CI: 0.62, 1.98, *P* = 0.734) Primary school overall (OR: 0.69, 95% CI: 0.56, 0.85, *P* < 0.001) ∗ Middle school overall (OR: 0.37, 95% CI: 0.14, 1.00, *P* = 0.050)	[82]
Retrospective time-trend study	China	6–15 y old	7350 children	Density of Western fast food	Overweight: defined as sex-age-specific BMI >85th percentile of the WS/T586–2018 Obesity: defined as sex-age-specific BMI >95th percentile of the WS/T586–2018	Age, gender, neighborhood SES, urbanicity, a random effect for the 3 waves	Overweight/obesity Overall (OR: 1.34, 95% CI: 1.12, 1.60, *P* < 0.001) ∗ Wave 1 (OR: 1.01, 95% CI: 0.76, 1.34, *P* = 0.949) Wave 2 (OR: 1.25, 95% CI: 0.93, 1.67, *P* = 0.141) Wave 3 (OR: 2.70, 95% CI: 1.58, 4.62, *P* < 0.001) ∗ Primary school overall (OR: 1.34, 95% CI: 1.10, 1.64, *P*: 0.004) ∗ Middle school overall (OR: 1.37, 95% CI: 0.96, 1.97, *P*: 0.086) Obesity Overall (OR: 1.68, 95% CI: 1.29, 2.14, *P* < 0.001) ∗ Wave 1 (OR: 1.42, 95% CI: 0.87, 2.30, *P*: 0.161) Wave 2 (OR: 1.45, 95% CI: 0.97, 2.18, *P*: 0.073) Wave 3 (OR: 2.47, 95% CI: 1.21, 5.05, *P*: 0.013) ∗ Primary school overall (OR: 1.60, 95% CI: 1.24, 2.08, *P* < 0.001) ∗ Middle school overall (OR: 3.33, 95% CI: 1.04, 10.61, *P*: 0.042) ∗	[82]
Density of supermarkets								
Longitudinal cohort study	United Kingdom	Children aged 4–5 y old (reception year); children aged 10–11 y old (year 6)	14,084 children at ages 4–5 and 5637 children at ages 10–11	Density of supermarkets	Overweight or obesity: BMI ≥85th centile for sex and age	Maternal BMI and smoking in early pregnancy, educational attainment, ethnicity and parity, clustering of observations within areas, relative density of unhealthy food outlets	LSOA (mean populations of 1500 and an area of 4 km^2^): overweight or obesity at ages 4–5 (RR: 1.015, 95% CI: 0.976, 1.056); at ages 10–11 (RR: 0.959, 95% CI: 0.914, 1.005) MSOA (populations of 7000 and an area of 21 km^2^): overweight or obesity at ages 4–5 (RR: 1.018, 95% CI: 0.972, 1.066); at ages 10–11 (RR: 0.972, 95% CI: 0.916, 1.032)	[57]
Ecologic Study	United States	—	3140 children	Number of grocery stores/supermarkets (per 1000 county residents)	Overweight/obesity rates	—	Overweight/obesity rate: *β*: 0.0001, not significant at the national level	[71]
Cross-sectional study	Japan	Fifth-to ninth-grade children (around 10–15 y old)	7277 children (3787 boys and 3490 girls)	Density of supermarkets and department stores in each school district	Obesity: based on the percent Overweight, ≥20% was considered obese, whereas <20% was considered non-obese	Age, gender, school district area, population density, and physical activity environment	Obesity: OR: 1.006, 95% CI: 0.799, 1.267	[72]
Cross-sectional study	Mexico	12–19 y old	2239 adolescents (1063 males and 1176 females)	Supermarkets store density (quotient of the number of stores over the number of inhabitants within the municipality (per 10,000 inhabitants)	Overweight: *z*-scores ≥1 SD and ≤ 2 SD Obesity: *z*-scores >2 SD	Sex and SES	Overweight or obesity: *β*: 0.426, 95% CI: 0.159, 0.692, *P*: 0.002 ∗	[65]
Longitudinal study	United States	High school students in grades 9–12	361,942 students	Supermarket (<0.25 miles)	Adolescent obesity	Race/ethnicity, gender, eligibility for free or reduced-price lunch, native born, special education, speaking English at home, age, grade, the building type of their home, and the number of total food outlet within 10 blocks	Adolescent obesity: *β*: –0.012, SE: 0.003 ∗, predicted likelihood: 0.155, 95% CI: 0.148, 0.163)	[77]
Cross-sectional study	United States	3–15 y old	627 children	Presence of supermarket within 0.25 mile	BMI *z*-score	Active commuting, race/ethnicity, income, >200% federal poverty level, presence of small grocery store within 0.25 miles, number of unhealthy outlets within 0.25 mile	BMI *z*-score (have vs. not have supermarkets within 0.25 mile): *β*: –0.54, *P*: 0.054 Among active commuters: BMI *z*-score (have vs. not have supermarkets within 0.25 mile): *β*: –0.29, *P*: 0.45) Among nonactive commuters: BMI *z*-score (have vs. not have supermarkets within 0.25 mile): *β*: –0.52, *P*: 0.17)	[58]
Retrospective time-trend study	China	6–15 y old	7350 children	Density of supermarket	Overweight: defined as sex-age-specific BMI >85th percentile of the WS/T586–2018 Obesity: defined as sex-age-specific BMI >95th percentile of the WS/T586–2018	Age, gender, neighborhood SES, urbanicity, a random effect for the 3 waves	Overweight/obesity Overall (OR: 1.05, 95% CI: 0.94, 1.18, *P*: 0.385) Wave 1 (OR: 1.51, 95% CI: 1.28, 1.78, *P* < 0.001) ∗ Wave 2 (OR: 0.65, 95% CI: 0.53, 0.80, *P* < 0.001) ∗ Wave 3 (OR: 0.83, 95% CI: 0.59, 1.17, *P*: 0.287) Primary school overall (OR: 1.11, 95% CI: 0.98, 1.27, *P*: 0.105) Middle school overall (OR: 1.00, 95% CI: 0.80, 1.26, *P*: 0.977) Obesity Overall (OR: 1.05, 95% CI: 0.89, 1.25, *P*: 0.542) Wave 1 (OR: 1.29, 95% CI: 1.00, 1.68, *P*: 0.052) Wave 2 (OR: 0.85, 95% CI: 0.63, 1.14, *P*: 0.272) Wave 3 (OR: 0.83, 95% CI: 0.50, 1.37, *P*: 0.467) Primary school overall (OR: 1.07, 95% CI: 0.90, 1.27, *P*: 0.452) Middle school overall (OR: 0.86, 95% CI: 0.36, 2.04, *P*: 0.727)	[82]
Difference-in-differences design	United States	Kindergarten through 12th grade (K-12) enrolled in NYC public schools	54,728 residentially stable public-school students	Exposure to a Food Retail Expansion to Support Health (FRESH) supermarket within 0.50 miles	BMI *z*-score Obesity (likelihood)	Individual-level characteristics (sex, age, race and ethnicity, grade in school, eligibility for free or reduced-priced lunch, students with a disability), census tract-level characteristics [population count, household median income, population race and ethnicity, population race and ethnicity, age ≥18 y, sex, college or more education, living below federal poverty level (FPL)]	BMI z-score all: DiD: –0.04, 95% CI: –0.06, –0.02, *P* < 0.001 ∗ Obesity (likelihood) all: DiD: –0.01, 95% CI: –0.02, –0.002, *P* < 0.05 ∗	[83]
Difference-in-differences design	United States	Kindergarten through 12th grade (K-12) enrolled in NYC public schools	54,728 residentially stable public-school students	Exposure to a new FRESH supermarket within 0.50 miles	BMI *z*-score Obesity (likelihood)	Individual-level characteristics (sex, age, race and ethnicity, grade in school, eligibility for free or reduced-priced lunch, students with a disability), census tract-level characteristics (population count, household median income, population race and ethnicity, population race and ethnicity, age ≥18 y, sex, college or more education, living below FPL)	BMI *z*-score all: DiD: –0.07, 95% CI: –0.11, –0.03, *P* < 0.001 ∗ Obesity (likelihood) all: DiD: –0.02, 95% CI: –0.03, –0.003, *P* < 0.05 ∗	[83]
Difference-in-differences design	United States	Kindergarten through 12th grade (K-12) enrolled in NYC public schools	54,728 residentially stable public-school students	Exposure to a renovated FRESH supermarket within 0.50 miles	BMI *z*-score Obesity (likelihood)	Individual-level characteristics (sex, age, race and ethnicity, grade in school, eligibility for free or reduced-priced lunch, students with a disability), census tract-level characteristics (population count, household median income, population race and ethnicity, population race and ethnicity, age ≥18 y, sex, college or more education, living below FPL)	BMI *z*-score all: DiD: –0.03, 95% CI: –0.06, –0.01, *P* < 0.05 ∗ Obesity (likelihood) all: DiD: –0.01, 95% CI: –0.02, 0.001	[83]
Longitudinal study	Mexico	5–19 y old	7507 children (3663 boys and 3844 girls)	Density of supermarkets (for each municipality)	BMI	Age, age^2^, age^3^, parental education level, population density, and socioeconomic deprivation	BMI (quartile 2 vs. quartile 1 of the density of supermarkets): *β*: –0.068 kg/m^2^, 95% CI: –0.380, 0.243, *P* = 0.666 BMI (quartile 3 vs. quartile 1 of the density of supermarkets): *β*: 0.310 kg/m^2^, 95% CI: 0.145, 0.476, *P* < 0.001 ∗ BMI (quartile 4 vs. quartile 1 of the density of supermarkets): *β*: 0.164 kg/m^2^, 95% CI: –0.074, 0.403, *P* = 0.177	[84]
Cohort study	United States	0–15 y old	28,359 children (14,657 boys and 13,702 girls)	Residence in low-income, low-food access (defined as low-income neighborhoods where the nearest supermarket is >0.5 miles for urban areas or >10 miles for rural areas) neighborhoods in pregnancy	BMI *z*-score Obesity risk: age- and sex-specific BMI ≥95th percentile Severe obesity risk: age- and sex-specific BMI ≥120% of the 95th percentile	Mother's age, educational level, and insurance during pregnancy, number of individuals in household, prenatal cigarette smoking, prenatal secondhand smoke exposure, parity, and child's birth year	BMI *z*-score at age 5 y: *β*: 0.07, 95% CI: 0.03, 0.11 ∗ BMI *z*-score at age 10 y: *β*: 0.11, 95% CI: 0.06, 0.17 ∗ BMI *z*-score at age 15 y: *β*: 0.16; 95% CI: 0.07, 0.24 ∗ Obesity risk at age 5 y: RR: 1.37; 95% CI: 1.21, 1.55 ∗ Obesity risk at age 10 y: RR: 1.71; 95% CI: 1.37, 2.12 ∗ Obesity risk at age 15 y: RR: 2.08; 95% CI: 1.53, 2.83 ∗ Severe obesity risk at age 5 y: RR: 1.21; 95% CI: 0.95, 1.53 ∗ Severe obesity risk at age 10 y (RR: 1.54; 95% CI: 1.20, 1.99) ∗ Severe obesity risk at age 15 y (RR: 1.92; 95% CI: 1.32, 2.80) ∗	[85]
Density of convenience stores								
Cross-sectional study	China	Mean age of the students was 10.2 y (SD: 0.33)	2201 children (1107 boys and 1094 girls)	Ten additional convenience stores near schools	Obesity ≥95th centile for age and sex	Age, gender, districts, father's obesity, mother's obesity, family type, father's education, and mother's education, diet score, meeting the recommendation of MVPA, and meeting the recommendation of screen time	Obesity: OR: 1.13, 95% CI: 1.03, 1.24, *P* = 0.011 ∗	[86]
Cross-sectional study	China	Mean age of the students was 10.2 y (SD: 0.33)	2201 children (1107 boys and 1094 girls)	More convenience stores near schools (≥24) vs. less convenience stores near schools (<24)	Obesity ≥95th centile for age and sex	Age, gender, districts, father's obesity, mother's obesity, family type, father's education, and mother's education, diet score, meeting the recommendation of MVPA, and meeting the recommendation of screen time	Obesity: OR: 1.49, 95% CI: 1.09, 2.03, *P* = 0.013 ∗	[86]
Ecologic Study	United States	—	3140 children	Number of convenience stores (per 1000 county residents)	Overweight/obesity rates	—	Overweight/obesity rate: *β*: 0.0007, *P* < 0.001 at the national level ∗	[71]
Cross-sectional study	Japan	Fifth-to ninth-grade children (around 10–15 y old)	7277 children (3787 boys and 3490 girls)	Density of convenience stores in each school district	Obesity: based on the percent Overweight, ≥20% was considered obese, whereas <20% was considered non-obese	Age, gender, school district area, population density, and physical activity environment	Obesity: OR: 1.250, 95% CI: 1.024, 1.525, *P* < 0.05 ∗	[72]
Ecologic study	United States	5–19 y old, grades K-12	2064 census tracts	Percentage of convenience stores	Hotspots of childhood obesity	% children, % female, % non-Hispanic Black, % Hispanic, median household income, % fast food restaurants	Hotspots of childhood obesity: *β*: 10.80, 95% CI: 7.62, 13.98, *P* < 0.001 ∗	[75]
Longitudinal cohort study	United States	3–15 y old	449 children (239 boys and 210 girls)	Change in convenience store counts	BMI *z*-score change category: a 3-level ordered categorical variable was created based on the change in BMI *z*-score: negative change (BMI *z*-score decreased >0.50), no change (BMI *z*-score did not change >0.50 in absolute value), and positive change (BMI *z*-score increased >0.50)	Child age, sex, and race, whether the child was classified as obese at T1, number of months between T1 and T2, as well as for food environment (counts of different outlet types) at T1, and difference between T2-T1 for the following variables: household income level as ratio of federal poverty level, total population and median income at the census block group level	BMI *z*-score change category: OR: 1.117, *P* = 0.007 ∗	[87]
Longitudinal study	United States	From kindergarten (K) to 12th grade. The age range of the children in this study is ∼5–18 y old	106 schools	Number of convenience stores within a 400-m roadway network of each school	Obesity rate	Time, school level, % of students eligible for FRPM, % of Hispanic students, % of non-Hispanic Black students	Obesity rate at baseline: *β*: 0.005, 95% CI: –0.011, 0.002, *P* = 0.144 Change in obesity rate over time: *β*: 0.001, 95% CI: –0.001, 0.003, *P* = 0.171	[49]
Cross-sectional study	Mexico	12–19 y old	2239 adolescents (1063 males and 1176 females)	Convenience store density (quotient of the number of stores over the number of inhabitants within the municipality (per 10,000 inhabitants)	Overweight: *z*-scores ≥1 SD and ≤ 2 SD Obesity: *z*-scores >2 SD	Sex and SES	Overweight or obesity: *β*: 0.014, 95% CI: –0.019, 0.048, *P* = 0.395	[65]
Cross-sectional study	Norway	Seventh-graders in primary schools in Oslo (around 12–13 y old)	802 adolescents (369 boys and 433 girls)	Number of convenience stores within an 800 m radius buffer around the residential neighborhood address of the participant	Overweight: was defined by applying the age-and sex-specific IOTF BMI (kg/m^2^) cut-offs	Ethnicity of the participants, parental education, and neighborhood deprivation level	Overweight: OR: 1.04, 95% CI: 0.97, 1.12	[56]
Cross-sectional study	Mexico	8–10 y old	218 children (120 girls and 98 boys)	Convenience stores density (n/250 m buffer)	% BF Abdominal fat % BMI *z*-score	Sex, age, caretaker's educational level, physical activity, and proximity to school	% BF: *β*: 0.145, 95% CI: 0.048, 0.241, *P* = 0.004 ∗ Abdominal fat %: *β*: 0.206, 95% CI: 0.069, 0.343, *P* = 0.003 ∗ BMI z-score: *β*: 0.028, 95% CI: 0.005, 0.062, *P* = 0.005 ∗	[53]
Cross-sectional study	Malaysia	5–17 y old	880 children (405 boys and 475 girls)	Presence of convenience stores (within 1000 m)	Overweight: based on the IOTF 2012 BMI criteria	Age, sex, place of residence, household income, state of residence	Adjusted for sociodemographic factors and parks: overweight (have vs. not have convenience stores within 1000 m): PR: 1.60, 95% CI: 1.09, 2.34, *P* = 0.016 ∗ Adjusted for sociodemographic factors and sports facilities: overweight (have vs. not have convenience stores within 1000 m): PR: 1.68, 95% CI: 1.14, 2.47, *P* = 0.009 ∗	[88]
Retrospective time-trend study	China	6–15 y old	7350 children	Density of convenient store	Overweight: defined as sex-age-specific BMI >85th percentile of the WS/T586–2018 Obesity: defined as sex-age-specific BMI >95th percentile of the WS/T586–2018	Age, gender, neighborhood SES, urbanicity, a random effect for the 3 waves	Overweight/obesity Overall (OR: 1.06, 95% CI: 0.94, 1.19, *P* = 0.370) Wave 1 (OR: 0.89, 95% CI: 0.76, 1.05, *P* = 0.181) Wave 2 (OR: 1.20, 95% CI: 0.96, 1.49, *P* = 0.111) Wave 3 (OR: 1.03, 95% CI: 0.66, 1.62, *P* = 0.890) Primary school overall (OR: 1.09, 95% CI: 0.95, 1.25, *P* = 0.234) Middle school overall (OR: 1.11, 95% CI: 0.88, 1.40, *P* = 0.358) Obesity Overall (OR: 1.11, 95% CI: 0.92, 1.32, *P* = 0.260) Wave 1 (OR: 0.91, 95% CI: 0.70, 1.18, *P* = 0.479) Wave 2 (OR: 1.19, 95% CI: 0.88, 1.60, *P* = 0.265) Wave 3 (OR: 0.83, 95% CI: 0.49, 1.43, *P* = 0.510) Primary school overall (OR: 1.10, 95% CI: 0.92, 1.33, *P* = 0.303) Middle school overall (OR: 1.16, 95% CI: 0.59, 2.51, *P* = 0.749)	[82]
Longitudinal study	Mexico	5–19 y old	7507 children (3663 boys and 3844 girls)	Density of convenience stores (for each municipality)	BMI	Age, age^2^, age^3^, parental education level, population density, and socioeconomic deprivation	BMI (quartile 2 vs. quartile 1 of the density of convenience stores): *β*: –0.035 kg/m^2^, 95% CI: –0.178, 0.247, *P* = 0.750 BMI (quartile 3 vs. quartile 1 of the density of convenience stores): *β*: 0.059 kg/m^2^, 95% CI: –0.118, 0.236, *P* = 0.515 BMI (quartile 4 vs. quartile 1 of the density of convenience stores): *β*: 0.077 kg/m^2^, 95% CI: –0.168, 0.322, *P* = 0.536	[84]
Density of farmer's markets/ fruit and vegetable stores								
Ecologic Study	United States	—	3140 children	Number of farmer's markets (per 1000 county residents)	Overweight/obesity rates	—	Overweight/obesity rate: *β*: 0.0002, *P* < 0.01 at the national level ∗	[71]
Cross-sectional study	Brazil	12–19 y old	504 adolescents (259 males and 245 females)	Presence of street markets and whole food markets within a 500 m buffer zone of the adolescents' homes	Overweight/obese: ≥ percentile 85th	Sex, census tract, race, neighborhood violence, years of residence, physical activity and the intra-municipal HDI	Overweight/obese (have vs. not have street markets and whole food markets within a 500 m buffer zone): OR: 1.18, 95% CI: 0.74, 1.87, *P* = 0.49	[66]
Retrospective time-trend study	China	6–15 y old	7350 children	Density of fruit/vegetable market	Overweight: defined as sex-age-specific BMI >85th percentile of the WS/T586–2018 Obesity: defined as sex-age-specific BMI >95th percentile of the WS/T586–2018	Age, gender, neighborhood SES, urbanicity, a random effect for the 3 waves	Overweight/obesity Overall (OR: 0.84, 95% CI: 0.77, 0.92, *P* < 0.001) ∗ Wave 1 (OR: 0.76, 95% CI: 0.68, 0.84, *P* < 0.001) ∗ Wave 2 (OR: 0.80, 95% CI: 0.65, 0.99, *P* = 0.041) ∗ Wave 3 (OR: 1.14, 95% CI: 0.82, 1.58, *P* = 0.428) Primary school overall (OR: 0.81, 95% CI: 0.73, 0.89, *P* < 0.001) ∗ Middle school overall (OR: 0.89, 95% CI: 0.75, 1.06, *P* = 0.205) Obesity Overall (OR: 0.93, 95% CI: 0.82, 1.06, *P* = 0.279) Wave 1 (OR: 0.89, 95% CI: 0.74, 1.07, *P* = 0.199) Wave 2 (OR: 0.80, 95% CI: 0.59, 1.08, *P* = 0.140) Wave 3 (OR: 1.10, 95% CI: 0.71, 1.71, *P* = 0.664) Primary school overall (OR: 0.93, 95% CI: 0.82, 1.06, *P* = 0.320) Middle school overall (OR: 0.72, 95% CI: 0.38, 1.37, *P* = 0.319)	[82]
Longitudinal study	Mexico	5–19 y old	7507 children (3663 boys and 3844 girls)	Density of fruit and vegetable stores (for each municipality)	BMI	Age, age^2^, age^3^, parental education level, population density, and socioeconomic deprivation	BMI (quartile 2 vs. quartile 1 of the density of fruit and vegetable stores): *β*: –0.455 kg/m^2^, 95% CI: –0.704, –0.207, *P* < 0.001 ∗ BMI (quartile 3 vs. quartile 1 of the density of fruit and vegetable stores): *β*: –0.733 kg/m^2^, 95% CI: –1.072, –0.394, *P* < 0.001 ∗ BMI (quartile 4 vs. quartile 1 of the density of fruit and vegetable stores): *β*: –0.838 kg/m^2^, 95% CI: –1.291, –0.385, *P* < 0.001 ∗	[84]
Density of restaurants								
Ecologic Study	United States	—	3140 children	Number of full-service restaurants (per 1000 county residents)	Overweight/obesity rates	—	Overweight/obesity rate: *β*: –0.0009, *P* < 0.001 at the national level ∗	[71]
Cross-sectional study	Japan	Fifth-to ninth-grade children (around 10–15 y old)	7277 children (3787 boys and 3490 girls)	Density of casual restaurants in each school district	Obesity: based on the percent Overweight, ≥20% was considered obese, whereas <20% was considered non-obese	Age, gender, school district area, population density, and physical activity environment	Obesity: OR: 0.560, 95% CI: 0.295, 1.063	[72]
Cross-sectional study	Brazil	12–19 y old	504 adolescents (259 males and 245 females)	Presence of restaurants within a 500 m buffer zone of the adolescents' homes	Overweight/obese: ≥ percentile 85th	Sex, census tract, race, neighborhood violence, years of residence, physical activity and the intra-municipal HDI	Overweight/obese (have vs. not have restaurants within a 500 m buffer zone): OR: 1.19, 95% CI: 0.71, 2.01, *P* = 0.51	[66]
Cross-sectional study	Brazil	School children aged 7–14 y old	2206 children (1040 boys and 1166 girls)	Availability (≥1) and use of restaurants (categorized into 2 categories, "did not use": never and rarely, and "used": weekly, twice a month, and monthly)	Overweight (including obesity) BMI/age and sex ≥ +1 z-scores	Schoolchildren age, schoolchildren sex, maternal education level, area-level population density, and area-level median income	400 m buffer: Overweight (restaurants were available and not used vs. restaurants were not available and not used): OR: 1.40, 95% CI: 1.07, 1.84 ∗ Overweight (restaurants were available and used vs. restaurants were not available and not used): OR: 1.48, 95% CI: 1.14, 1.93 ∗ 800 m buffer: Overweight (restaurants were available and not used vs. restaurants were not available and not used): OR: 1.04, 95% CI: 0.69, 1.58 Overweight (restaurants were available and used vs. restaurants were not available and not used): OR: 1.06, 95% CI: 0.71, 1.60	[89]
Cross-sectional study	Norway	Seventh-graders in primary schools in Oslo (around 12–13 y old)	802 adolescents (369 boys and 433 girls)	Number of restaurants within an 800 m radius buffer around the residential neighborhood address of the participant	Overweight: was defined by applying the age-and sex-specific IOTF BMI (kg/m^2^) cut-offs	Ethnicity of the participants, parental education, and neighborhood deprivation level	Overweight: OR: 1.01, 95% CI: 0.97, 1.05	[56]
Longitudinal study	United States	High school students in grades 9–12	361,942 students	Wait-service restaurant (<0.25 miles)	Adolescent obesity	Race/ethnicity, gender, eligibility for free or reduced-price lunch, native born, special education, speaking English at home, age, grade, the building type of their home, and the number of total food outlet within 10 blocks	Adolescent obesity: *β*: –0.009,SE: 0.001 ∗, predicted likelihood: 0.156, 95% CI: 0.153, 0.159	[77]
Longitudinal study	United States	High school students in grades 9–12	361,942 students	Wait-service restaurant (0.25–0.50 miles)	Adolescent obesity	Race/ethnicity, gender, eligibility for free or reduced-price lunch, native born, special education, speaking English at home, age, grade, the building type of their home, and the number of total food outlet within 10 blocks	Adolescent obesity: *β*: –0.009, SE: 0.003, predicted likelihood: 0.162, 95% CI: 0.160, 0.164	[77]
Density of grocery stores								
Longitudinal cohort study	United States	3–15 y old	449 children (239 boys and 210 girls)	Change in small grocery store counts	BMI *z*-score change category: a 3-level ordered categorical variable was created based on the change in BMI *z*-score: negative change (BMI *z*-score decreased >0.50), no change (BMI *z*-score did not change >0.50 in absolute value), and positive change (BMI *z*-score increased >0.50)	Child age, sex, and race, whether the child was classified as obese at T1, number of months between T1 and T2, as well as for food environment (counts of different outlet types) at T1, and difference between T2-T1 for the following variables: household income level as ratio of federal poverty level, total population and median income at the census block group level	BMI *z*-score change category: OR: 0.627, *P* < 0.05 ∗	[87]
Longitudinal study	United States	From kindergarten (K) to 12th grade. The age range of the children in this study is ∼5–18 y old	106 schools	Presence of small grocery store within a 400-m roadway network of each school	Obesity rate	Time, school level, % of students eligible for FRPM, % of Hispanic students, % of non-Hispanic Black students	Obesity rate at baseline (have vs. not have small grocery stores within a 400-meter roadway network of each school): *β*: –0.010, 95% CI: –0.026, 0.007, *P* = 0.252 Change in obesity rate over time (have vs. not have small grocery stores within a 400-m roadway network of each school): *β*: 0.000, 95% CI: –0.005, 0.005, *P* = 0.928	[49]
Cross-sectional study	Mexico	12–19 y old	2239 adolescents (1063 males and 1176 females)	Grocery store density [quotient of the number of stores over the number of inhabitants within the municipality (per 10,000 inhabitants)]	Overweight: *z*-scores ≥1 SD and ≤ 2 SD Obesity: *z*-scores >2 SD	Sex and SES	Overweight or obesity: *β*: 0.007, 95% CI: 0.002, 0.012, *P* = 0.009 ∗	[65]
Cross-sectional study	Brazil	School children aged 7–14 y old	2206 children (1040 boys and 1166 girls)	Availability (≥1) and use of grocery stores (categorized into 2 categories, "did not use": never and rarely, and "used": weekly, twice a month, and monthly)	Overweight (including obesity) BMI/age and sex ≥ + 1 *z*-scores	Schoolchildren age, schoolchildren sex, maternal education level, area-level population density, and area-level median income	400 m buffer: Overweight (grocery stores were available and used vs. grocery stores were not available and/or not used): OR: 1.09, 95% CI: 0.84, 1.41 Overweight (grocery stores were available and used vs. grocery stores were not available and/or not used): OR: 0.76, 95% CI: 0.52, 1.13	[89]
Cross-sectional study	Norway	Seventh-graders in primary schools in Oslo (around 12–13 y old)	802 adolescents (369 boys and 433 girls)	Number of grocery stores within an 800 m radius buffer around the residential neighborhood address of the participant	Overweight: was defined by applying the age-and sex-specific IOTF BMI (kg/m^2^) cut-offs	Ethnicity of the participants, parental education, and neighborhood deprivation level	Overweight: OR: 1.05, 95% CI: 0.95, 1.15	[56]
Cross-sectional study	United States	Children between 3 and 15 y old	627 children	Presence of small grocery store within 0.25 mile	BMI *z*-score	Active commuting, race/ethnicity, income, >200% federal poverty level, number of unhealthy outlets within 0.25 mile, presence of supermarket w/in 0.25 miles	BMI *z*-score (have vs. not have small grocery stores within 0.25 mile): *β*: 0.10, *P* = 0.64 Among active commuters: BMI *z*-score (have vs. not have small grocery stores within 0.25 mile): *β*: 0.60, *P* = 0.04 ∗ Among nonactive commuters: BMI *z*-score (have vs. not have small grocery stores within 0.25 mile): *β*: –0.41, *P* = 0.11	[58]
Density of nonalcoholic beverage store								
Cross-sectional study	Mexico	12–19 y old	2239 adolescents (1063 males and 1176 females)	Nonalcoholic beverage store density [quotient of the number of stores over the number of inhabitants within the municipality (per 10,000 inhabitants)]	Overweight: *z*-scores ≥1 SD and ≤ 2 SD Obesity: *z*-scores >2 SD	Sex and SES	Overweight or obesity: *β*: 0.036, 95% CI: –0.056, 0.128, *P* = 0.442	[65]
Density of bakeries and cafeterias								
Cross-sectional study	Brazil	12–19 y old	504 adolescents (259 males and 245 females)	Presence of bakeries and cafeterias within a 500 m buffer zone of the adolescents' homes	Overweight/obese: ≥ percentile 85th	Sex, census tract, race, neighborhood violence, years of residence, physical activity and the intra-municipal HDI	Overweight/obese (have vs. not have bakeries and cafeterias within a 500 m buffer zone): OR: 0.99, 95% CI: 0.44, 2.22, *P* = 0.98	[66]
Density of pizzerias								
Cross-sectional study	Brazil	12–19 y old	504 adolescents (259 males and 245 females)	Presence of pizzerias within a 500 m buffer zone of the adolescents' homes	Overweight/obese: ≥ percentile 85th	Sex, census tract, race, neighborhood violence, years of residence, physical activity and the intra-municipal HDI	Overweight/obese (have vs. not have pizzerias within a 500 m buffer zone): OR: 1.19, 95% CI: 0.67, 2.14, *P* = 0.55	[66]
Density of snack outlets								
Cross-sectional study	Brazil	7–14 y old	2206 children (1040 boys and 1166 girls)	Availability (≥1) and use of snack outlets (categorized into 2 categories, "did not use": never and rarely, and "used": weekly, twice a month, and monthly	Overweight (including obesity) BMI/age and sex ≥ + 1 *z*-scores	Schoolchildren age, schoolchildren sex, maternal education level, area-level population density, and area-level median income	400 m buffer: Overweight (snack outlets were available and used vs. snack outlets were not available and/or not used): OR: 0.95, 95% CI: 0.73, 1.23 800 m buffer: Overweight (snack outlets were available and used vs. snack outlets were not available and/or not used): OR: 1.19, 95% CI: 0.85, 1.67	[89]
Density of small food retail stores								
Longitudinal study	Mexico	5–19 y old	7507 children (3663 boys and 3844 girls)	Density of small food retail stores (for each municipality)	BMI	Age, age^2^, age^3^, parental education level, population density, and socioeconomic deprivation	BMI (quartile 2 vs. quartile 1 of the density of small food retail stores): *β*: –0.110 kg/m^2^, 95% CI: –0.398, 0.178, *P* = 0.455 BMI (quartile 3 vs. quartile 1 of the density of small food retail stores): *β*: –0.068 kg/m^2^, 95% CI: –0.446, 0.310, *P* = 0.723 BMI (quartile 4 vs. quartile 1 of the density of small food retail stores): *β*: 0.090 kg/m^2^, 95% CI: –0.449, 0.629, *P* = 0.743	[84]
Density of specialty food stores								
Longitudinal study	Mexico	5–19 y old	7507 children (3663 boys and 3844 girls)	Density of specialty food stores (for each municipality)	BMI	Age, age^2^, age^3^, parental education level, population density, and socioeconomic deprivation	BMI (quartile 2 vs. quartile 1 of the density of specialty food stores): *β*: –0.126 kg/m^2^, 95% CI: –0.400, 0.149, *P* = 0.369 BMI (quartile 3 vs. quartile 1 of the density of specialty food stores): *β*: –0.201 kg/m^2^, 95% CI: –0.601, 0.198, *P* = 0.323 BMI (quartile 4 vs. quartile 1 of the density of specialty food stores): *β*: –0.089 kg/m^2^, 95% CI: –0.697, 0.518, *P* = 0.774	[84]
Density of nutrition assistance program locations								
Longitudinal cohort study	United States	2–5 y old	3724 children (882 boys and 1842 girls)	Density of stores certified to accept benefits from the Special Supplemental Nutrition Program for WIC	BMI *z*-score	Age, sex, race/ethnicity, insurance, neighborhood (SES)	Mean BMI *z*-score (low density of stores certified to accept benefits from the WIC vs. no stores): *β*: –0.038, SE: 0.118, *P* = 0.749 Mean BMI *z*-score (moderate density of stores certified to accept benefits from the WIC vs. no stores): *β*: –0.005, SE: 0.138, *P* = 0.969 Mean BMI *z*-score (high density of stores certified to accept benefits from the WIC vs. no stores): *β*: 0.272, SE: 0.118, *P* = 0.021 ∗ Change in mean BMI *z*-score per month [low density of stores certified to accept benefits from the WIC vs. no stores × age (months)]: *β*: 0.003, SE: 0.003, *P* = 0.224 Change in mean BMI *z*-score per month [moderate density of stores certified to accept benefits from the WIC vs. no stores × age (months)]: *β*: 0.000, SE: 0.003, *P* = 0.926 Change in mean BMI *z*-score per month [high density of stores certified to accept benefits from the WIC vs. no stores × age (months)]: *β*: –0.004, SE: 0.003, *P* = 0.128	[54]
Difference-in-differences design	United States	Kindergarten through 12th grade (K-12) enrolled in NYC public schools	54,728 residentially stable public-school students	Exposure to a FRESH supermarket within 0.50 miles	BMI *z*-score Obesity (likelihood)	Individual-level characteristics (sex, age, race and ethnicity, grade in school, eligibility for free or reduced-priced lunch, students with a disability), census tract-level characteristics (population count, household median income, population race and ethnicity, population race and ethnicity, age ≥18 y, sex, college or more education, living below FPL)	BMI *z*-score all: DiD: –0.04, 95% CI: –0.06, –0.02, *P* < 0.001 ∗ Obesity (likelihood) all: DiD: –0.01, 95% CI: –0.02, –0.002, *P* < 0.05 ∗	[83]
Difference-in-differences design	United States	Kindergarten through 12th grade (K-12) enrolled in NYC public schools	54,728 residentially stable public-school students	Exposure to a new FRESH supermarket within 0.50 miles	BMI *z*-score Obesity (likelihood)	Individual-level characteristics (sex, age, race and ethnicity, grade in school, eligibility for free or reduced-priced lunch, students with a disability), census tract-level characteristics (population count, household median income, population race and ethnicity, population race and ethnicity, age ≥18 y, sex, college or more education, living below FPL)	BMI *z*-score all: DiD: –0.07, 95% CI: –0.11, –0.03, *P* < 0.001 ∗ Obesity (likelihood) all: DiD: –0.02, 95% CI: –0.03, –0.003, *P* < 0.05 ∗	[83]
Difference-in-differences design	United States	Kindergarten through 12th grade (K-12) enrolled in NYC public schools	54,728 residentially stable public-school students	Exposure to a renovated FRESH supermarket within 0.50 miles	BMI *z*-score Obesity (likelihood)	Individual-level characteristics (sex, age, race and ethnicity, grade in school, eligibility for free or reduced-priced lunch, students with a disability), census tract-level characteristics (population count, household median income, population race and ethnicity, population race and ethnicity, age ≥18 y, sex, college or more education, living below FPL)	BMI z-score all: DiD: –0.03, 95% CI: –0.06, –0.01, *P* < 0.05 ∗ Obesity (likelihood) all: DiD: –0.01, 95% CI: –0.02, 0.001	[83]
Distance of food outlets (categorized as healthy and unhealthy, or mixed outlets)								
Distance of healthy food outlets								
Cross-sectional study	Norway	Seventh-graders in primary schools in Oslo (around 12–13 y old)	802 adolescents (369 boys and 433 girls)	Shortest distance in meters from the residential neighborhood address of the participant to the nearest "healthy" food outlets (within an 800 m radius buffer)	Overweight: was defined by applying the age-and sex-specific IOTF BMI (kg/m^2^) cut-offs	Ethnicity of the participants, parental education, and neighborhood deprivation level	Overweight: OR: 0.99, 95% CI: 0.95, 1.02	[56]
Prospective observational study	United States	6–15 y old	4493 children	Healthy food access: represented a tract where ≥ 500 residents, or 33% of the tract population, lived > 1 mile (urban areas) or > 10 miles (rural areas) from the nearest supermarket or large grocery store	BMI *z*-score Overweight: classified as a BMI ≥85th and <95th percentile Obese as a BMI ≥95th percentile	Children's race/ethnicity, age, and insurance type, neighborhood median household income, and neighborhood race/ethnicity	Nonmovers: BMI *z*-score at time 2 (ample healthy food access vs. low healthy food access): *β*: –0.04, 95% CI: –0.08, 0.004, *P* = 0.078 Nonmovers: overweight or obese vs. healthy weight at time 2 (ample healthy food access vs. low healthy food access): OR: 0.88, 95% CI: 0.71, 1.08, *P* = 0.215 Movers: BMI *z*-score at time 2 (ample healthy food access vs. low healthy food access): *β*: –0.08, 95% CI: –0.17, 0.01, *P* = 0.079 Movers: overweight or obese vs. healthy weight at time 2 (ample healthy food access vs. low healthy food access): OR: 0.85, 95% CI: 0.58, 1.23, *P* = 0.377	[90]
Distance of unhealthy food outlets								
Cross-sectional study	United States	5–9 y old	1296 families with children	Proximity to unhealthy food retail options score	Child pBMI	Area deprivation index, online shopping experience, shopping by car, number of adults, number of children, child's gender, primary caregiver's education, public assistance receipt, household income, race/ethnicity, eating American foods at home and time in the United States	Food-insecure households: child pBMI (*β*: 0.482, 95% CI: –0.912, 1.876) Food-secure households: child pBMI (*β*: –0.096, 95% CI: –1.113, 0.921)	[52]
Cross-sectional study	Norway	Seventh-graders in primary schools in Oslo (around 12–13 y old)	802 adolescents (369 boys and 433 girls)	Shortest distance in meters from the residential neighborhood address of the participant to the nearest "unhealthy" food outlets (within an 800 m radius buffer)	Overweight: was defined by applying the age-and sex-specific IOTF BMI (kg/m^2^) cut-offs	Ethnicity of the participants, parental education, and neighborhood deprivation level	Overweight: OR: 1.00, 95% CI: 0.96, 1.03	[56]
Cross-sectional study	United States	Mean age was 11.3 y (± 3.7)	288 children (161 boys and 127 girls)	Limited food access: having at least a third of the population, or ≥200 people, outside the service area of a grocery store (>1 mile for urban block groups and 10 miles for rural block groups)	BMI	Age, sex, race, ethnicity, and insurance type	[Subset A, *n*: 158) BMI (raw): *β*: 3.27, 95% CI: 0.31, 6.22, *P* < 0.05] ∗ (Subset A, *n*: 158) BMI (percentage of 95th percentile): (*β*: 12.4, 95% CI: 0.24, 24.56, *P* < 0.05) ∗	[91]
Longitudinal study	United States	6–12 y old	1518 children (670 boys and 848 girls)	Low-food access: for urban areas: living >1 mile from a supermarket or grocery store; for rural areas: living >10 miles from a supermarket or grocery store	Increasing BMI slope trajectory	Baseline BMI, age, sex, Area Deprivation Index, park proximity >90%, normalized difference vegetation index	Increasing BMI slope trajectory: OR: 1.0, 95% CI: 0.78, 1.26	[92]
Distance of mixed food outlets								
Cross-sectional study	United States	10–14 y old	335 children (145 boys and 190 girls)	Distance to the nearest pharmacy, discount store, and variety store in miles	BMI *z*-score	Children's gender, caregivers' educational attainment, household income to federal poverty threshold, and household SNAP and Special Supplemental Nutrition Program for WIC participation	Using Extensively Revised Combined database (*β*: –0.069, 95% CI: –0.766, 0.656, *P* = 0.845) RefUSA – 2 NAICS (*β*: –0.451, 95% CI: –0.966, 0.074, *P* = 0.085) SNAP (*β*: –0.081, 95% CI: –0.716, 0.545, *P* = 0.799) CLF (*β*: –0.122, 95% CI: –0.727, 0.486, *P* = 0.688)	[64]
Cross-sectional study	United States	10–14 y old	335 children (145 boys and 190 girls)	Distance to the nearest supermarket, wholesale, club store, and large grocery store in miles	BMI *z*-score	Children's gender, caregivers' educational attainment, household income to federal poverty threshold, and household SNAP and Special Supplemental Nutrition Program for WIC participation	Using extensively revised Combined database (*β*: –0.095, 95% CI: –0.509, 0.341, *P* = 0.657) RefUSA – 2 NAICS (*β*: –0.322, 95% CI: –0.838, 0.062, *P* = 0.157) SNAP (*β*: –0.356, 95% CI: –0.802, 0.113, *P* = 0.121) CLF (*β*: –0.017, 95% CI: –0.423, 0.595, *P* = 0.948)	[64]
Cross-sectional study	United States	10–14 y old	335 children (145 boys and 190 girls)	Distance to the nearest corner, convenience, small and medium grocery store, and gas station store in miles	BMI *z*-score	Children's gender, caregivers' educational attainment, household income to federal poverty threshold, and household SNAP and Special Supplemental Nutrition Program for WIC participation	Using extensively revised combined database (*β*: –1.272, 95% CI: –2.435, –0.155, *P* = 0.030) ∗ SNAP (*β*: –0.829, 95% CI: –1.823, 0.234, *P* = 0.118) CLF (*β*: –0.268, 95% CI: –1.341, 0.745, *P* = 0.604)	[64]
Cross-sectional study	United States	10–14 y old	335 children (145 boys and 190 girls)	Distance to the nearest full and limited-service restaurants and carryout in miles	BMI *z*-score	Children's gender, caregivers' educational attainment, household income to federal poverty threshold, and household SNAP and Special Supplemental Nutrition Program for WIC participation	Using Extensively Revised Combined database (*β*: –1.396, 95% CI: –2.391, –0.339, *P* = 0.008) ∗ RefUSA – 2 NAICS (*β*: –1.138, 95% CI: –2.065, –0.241, *P* = 0.015) ∗ SNAP (*β*: –0.521, 95% CI: –1.045, 0.050, *P* = 0.058)	[64]
Distance to fast food outlets								
Longitudinal study	United States	Around 5–6 y old (kindergarten) to 17–18 y old (twelfth grade)	Fourth to sixth grade: 33,308 children (16,871 boys and 16,437 girls) Fourth to eighth grade: 10,597 children (5457 boys and 5140 girls) Sixth to eighth grade: 33,130 children (17,083 boys and 16,047 girls) Eighth to tenth grade: 34,758 children (18,005 boys and 16,753 girls)	Change in the distance of shortest-distance route between home and school	BMI *z*-score	Median household income, per cent population living in poverty, median gross rent, and educational attainment for Census block group in which student resides, school district fixed effects also included	Fourth to sixth grade (*β*: –0.002, SE: 0.002) Fourth to eighth grade (*β*: 0.003, SE: 0.003) Sixth to eighth grade (*β*: –0.001, SE: 0.002) Eighth to tenth grade (*β*: 0.000, SE: 0.002)	[70]
Ecologic Study	Rhode Island	2–17 y old	808 children	Mean distance to fast food	Overweight/obesity rates	Town level risk index	Town level overweight/obesity rates: *β*: –1.89, 95% CI: –4.12, 0.34, *P* = 0.0949	[93]
Longitudinal study	United States	Students in grades K-12	1,188,658 students	Nearest fast food outlet within 0.025–0.05 miles from home	Overweight: ≥85th percentile of the CDC growth charts Obesity: ≥95th percentile of the CDC growth charts BMI *z*-score	Year fixed effect, census tract fixed effect, student characteristics (race and ethnicity, gender, poverty status, recent immigrant, special education, limited English proficiency, grade and age) and housing characteristics (housing type and a public housing indicator)	Obesity (nearest fast food outlet within 0.025–0.05 miles buffer from home vs. nearest fast food outlet within 0–0.025 miles buffer from home): percent change: –4.4%, *P* < 0.01 ∗ Obesity/overweight (nearest fast food outlet within 0.025–0.05 miles buffer from home vs. nearest fast food outlet within 0–0.025 miles buffer from home): percent change: –2.9%, *P* < 0.01 ∗ BMI *z*-score (nearest fast food outlet within 0.025–0.05 miles buffer from home vs. nearest fast food outlet within 0–0.025 miles buffer from home): percent change: –5.4%, *P* < 0.01 ∗	[94]
Longitudinal study	United States	Students in grades K-12	1,188,658 students	Nearest fast food outlet within 0.05–0.1 miles from home	Overweight: ≥85th percentile of the CDC growth charts Obesity: ≥95th percentile of the CDC growth charts BMI *z*-score	Year fixed effect, census tract fixed effect, student characteristics (race and ethnicity, gender, poverty status, recent immigrant, special education, limited English proficiency, grade and age) and housing characteristics (housing type and a public housing indicator)	Obesity (nearest fast food outlet within 0.05–0.1 miles buffer from home vs. nearest fast food outlet within 0–0.025 miles buffer from home): percent change: –4.0%, *P* < 0.01 ∗ Obesity/overweight (nearest fast food outlet within 0.05–0.1 miles buffer from home vs. nearest fast food outlet within 0–0.025 miles buffer from home): percent change: –2.6%, *P* < 0.01 ∗ BMI *z*-score (nearest fast food outlet within 0.05–0.1 miles buffer from home vs. nearest fast food outlet within 0–0.025 miles buffer from home): percent change: –4.8%, *P* < 0.01 ∗	[94]
Longitudinal cohort study	United Kingdom	Main analysis based at age 16, robustness analyses at age 10	3585 children (1864 girls and 1721 boys)	Distance to the nearest fast food outlet	BMI	Gender, highest parental social class, land of residence, residing in London, residing in an urban area, ethnicity and birth weight, mother's BMI in 1980, adolescent's smoking status and household ownership of microwave	BMI: *β*: –0.0004, SE: 0.009, *P*: 0.967	[74]
Longitudinal cohort study	United Kingdom	Main analysis based at age 16, robustness analyses at age 10	3585 children (1864 girls and 1721 boys)	Distance squared to the nearest fast food outlet	BMI	Gender, highest parental social class, land of residence, residing in London, residing in an urban area, ethnicity and birth weight, mother's BMI in 1980, adolescent's smoking status and household ownership of microwave	BMI: *β*: –0.0004, SE: 0.000, *P* = 0.031 ∗	[74]
Longitudinal cohort study	United Kingdom	Main analysis based at age 16, robustness analyses at age 10	3585 children (1864 girls and 1721 boys)	Interaction term of fast food: duration × inverse distance	BMI	Gender, highest parental social class, land of residence, residing in London, residing in an urban area, ethnicity and birth weight, mother's BMI in 1980, adolescent's smoking status and household ownership of microwave	BMI: *β*: 0.011, SE: 0.010, *P* = 0.261	[74]
Longitudinal study	United States	K-12 NYC public-school students	143,859 K-12 NYC public-school students	Distance to nearest fast food from residual	Overweight: ≥85th percentile of the CDC growth charts Obesity: ≥95th percentile of the CDC growth charts	Gender, race/ethnicity, grade, primary language spoken at home, special education, limited English proficiency status, distance to other food outlets, year, and within-development variation	Obese: *β*: –0.062, SE: 0.019, *P* < 0.01 ∗ Overweight: *β*: –0.111, SE: 0.022, *P* < 0.01 ∗	[95]
Longitudinal study	United States	K-12 NYC public-school students	143,859 K-12 NYC public-school students	Distance to fast food for students near school	Overweight: ≥85th percentile of the CDC growth charts Obesity: ≥95th percentile of the CDC growth charts	Gender, race/ethnicity, grade, primary language spoken at home, special education, limited English proficiency status, distance to other food outlets, year, and within-development variation	Grade K-2 only: Obese (*β*: 0.019, SE: 0.039) Overweight (*β*: –0.074, SE: 0.046) Grades 3–5 only: Obese (*β*: –0.158, SE: 0.043, *P* < 0.001) Overweight (*β*: –0.191, SE: 0.047, *P* < 0.001) Grades 6–8 only: Obese (*β*: –0.168, SE: 0.043, *P* < 0.001) Overweight (*β*: –0.208, SE: 0.048, *P* < 0.001) Grades 9–12: Obese (*β*: –0.029, SE: 0.047) Overweight (*β*: –0.129, SE: 0.057, *P* < 0.05)	[95]
Cross-sectional study	Norway	Seventh-graders in primary schools in Oslo (around 12–13 y old)	802 adolescents (369 boys and 433 girls)	Shortest distance in meters from the residential neighborhood address of the participant to the nearest fast food restaurants (within an 800 m radius buffer)	Overweight: was defined by applying the age-and sex-specific IOTF BMI (kg/m^2^) cut-offs	Ethnicity of the participants, parental education, and neighborhood deprivation level	Overweight: OR: 1.00, 95% CI: 0.93, 1.08	[56]
Longitudinal study	China	2–18 y old	3244 children	Distance to fast food (main results were within 3 km, other distance also done)	Overweight/obese: IOTF BMI cut-offs	Equivalent household income, number of children in the household, whether the child has entered puberty, between 2 and 5 y of age, whether ≥1 grandparent is living with the child, the number of households, population density, number of schools (primary and secondary), and price of pork and vegetables	Within 2 km: overweight/obese (have vs. not have a fast food restaurant within 2 km): *β*: –0.008, SE: 0.044 Within 2.5 km: overweight/obese (have vs. not have a fast food restaurant within 2.5 km): *β*: 0.011, SE: 0.024 Within 3 km: overweight/obese (have vs. not have a fast food restaurant within 3 km): *β*: –0.013, SE: 0.020 Within 4 km: overweight/obese (have vs. not have a fast food restaurant within 4 km): *β*: –0.014, SE: 0.012 ∗ Within 5 km: overweight/obese (have vs. not have a fast food restaurant within 5 km): *β*: 0.028, SE: 0.031	[80]
Cross-sectional study	Netherlands	Primary-school-aged children	135 primary schools	Shortest Euclidean distance from the school to the nearest fast food outlet	Overweight prevalence in the subdistrict	Neighborhood disadvantage level around schools (based on the schools' disadvantage scores)	All fast food outlets (*β*: 11.54, 95% CI: –0.17, 23.25) Fast food restaurants (*β*: 13.91, 95% CI: –0.04, 27.86) Grillrooms and kebab shops (*β*: –1.60, 95% CI: –2.56, –0.63) ∗ Takeaway restaurants (*β*: –0.26, 95% CI: –1.34, 0.82)	[81]
Distance to supermarkets								
Ecologic Study	Rhode Island	2–17 y old	808 children	Mean distance to supermarket	Overweight/obesity rates	Town level risk index	Town level overweight/obesity rates: *β*: –0.48, 95% CI: –1.34, 0.38, *P* = 0.2625	[93]
Cross-sectional study	United Kingdom	10–11 y old	983 observations for each transport profile	Road distance to the third nearest supermarket (km)	Proportion of overweight children	Total annual household income, number of persons per hectare, proportion of households from the non-White group, the base 10-logarithm transformation, proportion of households with 1 or more cars, proportion of households with no dependent children, proportion of households in part-time employment	Proportion of overweight children: *β*: 0.88, SE: 0.19, *P* < 0.001 ∗	[96]
Cross-sectional study	United Kingdom	10–11 y old	983 observations for each transport profile	Euclidean distance to the third nearest supermarket (km)	Proportion of overweight children	Total annual household income, number of persons per hectare, proportion of households from the non-White group, the base 10-logarithm transformation, proportion of households with 1 or more cars, proportion of households with no dependent children, proportion of households in part-time employment	Proportion of overweight children: *β*: 1.64, SE: 0.42, *P* < 0.001 ∗	[96]
Cross-sectional study	United Kingdom	10–11 y old	983 observations for each transport profile	Walking distance to the third nearest supermarket	Proportion of overweight children	Total annual household income, number of persons per hectare, proportion of households from the non-White group, the base 10-logarithm transformation, proportion of households with 1 or more cars, proportion of households with no dependent children, proportion of households in part-time employment	Proportion of overweight children: *β*: 2.33, SE: 0.49, *P* < 0.001 ∗	[96]
Cross-sectional study	United Kingdom	10–11 y old	983 observations for each transport profile	Cycling distance to the third nearest supermarket	Proportion of overweight children	Total annual household income, number of persons per hectare, proportion of households from the non-White group, the base 10-logarithm transformation, proportion of households with 1 or more cars, proportion of households with no dependent children, proportion of households in part-time employment	Proportion of overweight children: *β*: 0.75, SE: 0.21, *P* < 0.001 ∗	[96]
Cross-sectional study	United Kingdom	10–11 y old	983 observations for each transport profile	Active distance to the third nearest supermarket	Proportion of overweight children	Total annual household income, number of persons per hectare, proportion of households from the non-White group, the base 10-logarithm transformation, proportion of households with 1 or more cars, proportion of households with no dependent children, proportion of households in part-time employment	Proportion of overweight children: *β*: 1.55, SE: 0.34, *P* < 0.001 ∗	[96]
Cross-sectional study	United Kingdom	10–11 y old	983 observations for each transport profile	Driving distance to the third nearest supermarket	Proportion of overweight children	Total annual household income, number of persons per hectare, proportion of households from the non-White group, the base 10-logarithm transformation, proportion of households with 1 or more cars, proportion of households with no dependent children, proportion of households in part-time employment	Proportion of overweight children: *β*: 0.88, SE: 0.19, *P* < 0.001 ∗	[96]
Cross-sectional study	United Kingdom	4–5 y old, from all state-maintained schools in England taking part in the National Child Measurement Program	6772 children	Road distance from postcode centroid to the nearest supermarket, log (distance)	Obesity (%)	Number of persons per hectare, interaction between distance and density, total annual household income, proportion of households from the ethnic minority groups, proportion of households with no qualification, proportion of households with adults not in employment	For England: Log (distance): obesity (%) (*β*: 0.448, SE: 0.079, *P* < 0.01) ∗ Distance × density (interaction between distance and density): obesity (%) (*β*: 0.001, SE: 0.002) ∗ For urban areas: Log (distance): obesity (%) (*β*: 0.323, SE: 0.115, *P* < 0.05) ∗ Distance × density (interaction between distance and density): obesity (%) (*β*: 0.001, SE: 0.003, *P* < 0.05) ∗	[97]
Distance to convenience store								
Ecologic Study	Rhode Island	2–17 y old	808 children	Mean distance to convenience store	Overweight/obesity rates	Town level risk index	Town level overweight/obesity rates: *β*: –3.67, 95% CI: –6.63, –0.71, *P* = 0.0165 ∗	[93]
Cross-sectional study	Norway	Seventh-graders in primary schools in Oslo (around 12–13 y old)	802 adolescents (369 boys and 433 girls)	Shortest distance in meters from the residential neighborhood address of the participant to the nearest convenience stores (within an 800 m radius buffer)	Overweight: was defined by applying the age-and sex-specific IOTF BMI (kg/m^2^) cut-offs	Ethnicity of the participants, parental education, and neighborhood deprivation level	Overweight: OR: 0.99, 95% CI: 0.94, 1.04	[56]
Cross-sectional study	Mexico	8–10 y old	218 children (120 girls and 98 boys)	Convenience stores proximity (m)	% BF Abdominal fat % BMI *z*-score	Sex, age, caretaker's educational level, physical activity, and proximity to school	% BF: *β*: –0.009, 95% CI: –0.017, –0.001, *P* = 0.025 ∗ Abdominal fat %: *β*: –0.012, 95% CI: –0.023, –0.001, *P* = 0.033 ∗ BMI *z*-score: *β*: –0.002, 95% CI: –0.004, –0.001, *P* = 0.003 ∗	[53]
Distance to grocery stores								
Ecologic study	Rhode Island	2–17 y old	808 children	Mean distance to grocery store	Overweight/obesity rates	Town level risk index	Town level overweight/obesity rates (*β*: –0.65, 95% CI: –1.34, 0.04, *P* = 0.0639)	[93]
Cross-sectional study	Norway	Seventh-graders in primary schools in Oslo (around 12–13 y old)	802 adolescents (369 boys and 433 girls)	Shortest distance in meters from the residential neighborhood address of the participant to the nearest grocery stores (within an 800 m radius buffer)	Overweight: was defined by applying the age-and sex-specific IOTF BMI (kg/m^2^) cut-offs	Ethnicity of the participants, parental education, and neighborhood deprivation level	Overweight: OR: 1.01, 95% CI: 0.91, 1.11	[56]
Distance to corner store								
Longitudinal study	United States	Students in grades K-12	1,188,658 students	Nearest corner store 0.05–0.1 within miles from home	Overweight: ≥85th percentile of the CDC growth charts Obesity: ≥95th percentile of the CDC growth charts BMI *z*-score	Year fixed effect, census tract fixed effect, student characteristics (race and ethnicity, gender, poverty status, recent immigrant, special education, limited English proficiency, grade and age) and housing characteristics (housing type and a public housing indicator)	Obesity (nearest corner store within 0.05–0.1 miles buffer from home vs. nearest corner store within 0–0.025 miles buffer from home): percent change: –2.1%, *P* < 0.05 ∗ Obesity/overweight (nearest corner store within 0.05–0.1 miles buffer from home vs. nearest corner store within 0–0.025 miles buffer from home): percent change: –1.1%, *P* < 0.10 BMI *z*-score (nearest corner store within 0.05–0.1 miles buffer from home vs. nearest corner store within 0–0.025 miles buffer from home): percent change: –2.4%, *P* < 0.01 ∗	[94]
Distance to specialty store								
Ecologic study	Rhode Island	2–17 y old	808 children	Mean distance to specialty store	Overweight/obesity rates	Town level risk index	Town level overweight/obesity rates: *β*: –0.66, 95% CI: –1.48, 0.16, *P* = 0.1119	[93]
Distance to restaurants								
Ecologic study	Rhode Island	2–17 y old	808 children	Mean distance to sit-down restaurants	Overweight/obesity rates	Town level risk index	Town level overweight/obesity rates: *β*: –3.85, 95% CI: –7.17, –0.53, *P* = 0.0185 ∗	[93]
Cross-sectional study	Norway	Seventh-graders in primary schools in Oslo (around 12–13 y old)	802 adolescents, 369 boys and 433 girls	Shortest distance in meters from the residential neighborhood address of the participant to the nearest restaurants (within an 800 m radius buffer)	Overweight: was defined by applying the age-and sex-specific IOTF BMI (kg/m^2^) cut-offs	Ethnicity of the participants, parental education, and neighborhood deprivation level	Overweight: OR: 0.97, 95% CI: 0.93, 1.02	[56]
Distance to snack and beverage stores								
Ecologic study	Rhode Island	2–17 y old	808 children	Mean distance to snack and beverage stores	Overweight/obesity rates	Town level risk index	Town level overweight/obesity rates: *β*: –2.53, 95% CI: –4.56, –0.49, *P* = 0.0086 ∗	[93]
Cross-sectional study	China	6–18 y old	41,220 children and adolescents	Distance to the nearest pastry/cold beverage shop (reaching ∼1200 m/1500 m/1600 m)	Risk of obesity among primary school students	Gender, age, gross domestic product (GDP) in Chinese Yuan, time spent on the daily commute to school, and household income	Risk of obesity among middle school students was at lowest when the distance reached 1500 m	[98]
Distance to farmers markets								
Ecologic study	Rhode Island	2–17 y old	808 children	Mean distance to others (include food markets)	Overweight/obesity rates	Town level risk index	Town level overweight/obesity rates: *β*: –2.52, 95% CI: –6.50, 1.47, *P* = 0.2086	[93]
Ecologic study	Rhode Island	2–17 y old	808 children	Mean distance to farmers markets	Overweight/obesity rates	Town level risk index	Town level overweight/obesity rates: *β*: –0.56, 95% CI: –1.49, 0.37, *P* = 0.2295	[93]
Distance to food assistance provider								
Ecologic study	Rhode Island	2–17 y old	808 children	Mean distance to food assistance provider	Overweight/obesity rates	Town level risk index	Town level overweight/obesity rates: *β*: –1.15, 95% CI: –2.34, 0.04, *P* = 0.0576	[93]
Distance to nutrition assistance program locations								
Ecologic study	Rhode Island	2–17 y old	808 children	Mean distance to Special Supplemental Nutrition Program for Women, Infants, and Children (WIC) locations	Overweight/obesity rates	Town level risk index	Town level overweight/obesity rates: *β*: –0.16, 95% CI: –0.68, 0.36, *P* = 0.5432	[93]
Ecologic study	Rhode Island	2–17 y old	808 children	Mean distance to SNAP locations	Overweight/obesity rates	Town level risk index	Town level overweight/obesity rates: *β*: –0.42, 95% CI: –0.74, –0.10, *P* = 0.0111 ∗	[93]
3A: Food affordability								
Healthy food subsidies/price reduction								
Longitudinal study	United States	From kindergarten (K) to 12th grade. The age range of the children in this study is ∼5–18 y old	106 schools	Percentage of students eligible for FRPM	Obesity rate	Time, school level, % of students eligible for FRPM, % of Hispanic students, % of non-Hispanic Black students	Obesity rate over time: *β*: 0.095, 95% CI: 0.021, 0.169, *P* = 0.011 ∗	[49]
Unhealthy food subsidies/price reduction								
Cross-sectional study	Brazil	Second- and fourth-grade students, mean 8.0 y	1036 children (569 girls and 467 boys)	Food sale in school surroundings	Overweight	—	Overweight (have food sale vs. not have food sale in school surroundings): OR: 1.45, 95% CI: 0.97, 2.17	[45]
Cross-sectional study	Brazil	Second- and fourth-grade students, mean 8.0 y	1036 children (569 girls and 467 boys)	Fried snacks sale in school surroundings	Overweight	—	Overweight (have fried snacks sale vs. not have fried snacks sale in school surroundings): OR: 1.38, 95% CI: 1.05, 1.81 ∗	[45]
Cross-sectional study	Brazil	Second- and fourth-grade students, mean 8.0 y	1036 children (569 girls and 467 boys)	Candy sale in school surroundings	Overweight	—	Overweight (have candy sale vs. not have candy sale in school surroundings): OR: 1.58, 95% CI: 1.11, 2.27 ∗	[45]
Cross-sectional study	Brazil	Second- and fourth-grade students, mean 8.0 y	1036 children (569 girls and 467 boys)	Sweetened beverage sale in school surroundings	Overweight	—	Overweight (have sweetened beverage sale vs. not have sweetened beverage sale in school surroundings): OR: 0.77, 95% CI: 0.59, 1.01	[45]
Access to subsidized food outlets								
Difference-in-differences design	United States	Kindergarten through 12th grade (K-12) enrolled in NYC public schools	54,728 residentially stable public-school students	Exposure to a FRESH supermarket within 0.50 miles	BMI *z*-score Obesity (likelihood)	Individual-level characteristics (sex, age, race and ethnicity, grade in school, eligibility for free or reduced-priced lunch, students with a disability), census tract-level characteristics (population count, household median income, population race and ethnicity, population race and ethnicity, age ≥18 y, sex, college or more education, living below FPL)	BMI *z*-score all: DiD: –0.04, 95% CI: –0.06, –0.02, *P* < 0.001 ∗ Obesity (likelihood) all: DiD: –0.01, 95% CI: –0.02, –0.002, *P* < 0.05 ∗	[83]
Difference-in-differences design	United States	Kindergarten through 12th grade (K-12) enrolled in NYC public schools	54,728 residentially stable public-school students	Exposure to a new FRESH supermarket within 0.50 miles	BMI *z*-score Obesity (likelihood)	Individual-level characteristics (sex, age, race and ethnicity, grade in school, eligibility for free or reduced-priced lunch, students with a disability), census tract-level characteristics (population count, household median income, population race and ethnicity, population race and ethnicity, age ≥18 y, sex, college or more education, living below FPL)	BMI *z*-score all: DiD: –0.07, 95% CI: –0.11, –0.03, *P* < 0.001 ∗ Obesity (likelihood) all: DiD: –0.02, 95% CI: –0.03, –0.003, *P* < 0.05 ∗	[83]
Difference-in-differences design	United States	Kindergarten through 12th grade (K-12) enrolled in NYC public schools	54,728 residentially stable public-school students	Exposure to a renovated FRESH supermarket within 0.50 miles	BMI *z*-score Obesity (likelihood)	Individual-level characteristics (sex, age, race and ethnicity, grade in school, eligibility for free or reduced-priced lunch, students with a disability), census tract-level characteristics (population count, household median income, population race and ethnicity, population race and ethnicity, age ≥18 y, sex, college or more education, living below FPL)	BMI z-score All: DiD: –0.03, 95% CI: –0.06, –0.01, *P* < 0.05 ∗ Obesity (likelihood) All: DiD: –0.01, 95% CI: –0.02, 0.001	[83]
4A: Food appeal								
Food advertisement exposure								
Longitudinal cohort study	United States	13–16 y old (mean age 14.1 ± 1.0 y for the 121 participants with 2-y follow-up data)	At baseline: *n*: 168 (86 girls and 82 boys) At the 2-y follow-up: *n*: 121 (61 girls and 60 boys)	Functional connectivity during exposure to unhealthy fast food commercials	BMI	Age, sex, and baseline pubertal stage	Baseline BMI: *r*: 0.341, nonparametric permutation *P* < 0.001 ∗ 2-y follow-up BMI (controlling for baseline BMI): *r*: 0.174, nonparametric permutation *P* = 0.031 ∗	[99]
Longitudinal cohort study	United States	13–16 y old (mean age 14.1 ± 1.0 y for the 121 participants with 2-y follow-up data)	At baseline: *n*: 168 (86 girls and 82 boys) At the 2-y follow-up: *n*: 121 (61 girls and 60 boys)	Functional connectivity during exposure to healthier fast food commercials	BMI	Age, sex, and baseline pubertal stage	Baseline BMI: *r*: 0.366, nonparametric permutation *P* < 0.001 ∗ 2-y follow-up BMI (controlling for baseline BMI): *r*: 0.103, nonparametric permutation *P* = 0.152	[99]
Cross-sectional study	United Kingdom	7–11 y old	2260 children (1258 boys and 1002 girls)	Commercial TV advertisement exposure	BMI	—	BMI: *β*: 0.005, SE: 0.003, *P* = 0.06	[100]
Cross-sectional study	United Kingdom	7–11 y old	2260 children (1258 boys and 1002 girls)	Noncommercial TV advertisement exposure	BMI	—	BMI: *β*: 0.008, SE: 0.004, *P* = 0.16	[100]
Modeling study	United Kingdom	0–17 y old	13,729,000 children	All foods and beverages high in fat, sugar, and salt (HFSS) advertising between 05:30 and 21:00	Reduction in number of children aged 5–17 y with obesity/overweight (IOTF cut points)	—	Reduction in number of children aged 5–17 y with obesity: 40,000 (95% UI: 12,000–81,000); 4.6% (95% UI: 1.4%–9.5%) Reduction in number of children aged 5–17 y with overweight: 120,000 (95% UI: 34,000–240,000); 3.6% (95% UI: 1.1%–7.4%)	[101]
Modeling study	United Kingdom	0–17 y old	13,729,000 children	All foods and beverages HFSS advertising between 05:30 and 21:00 displaced to between 21:00 and 05:30	Reduction in number of children aged 5–17 y with obesity/overweight (IOTF cut points)	—	Reduction in number of children aged 5–17 y with obesity: 12,000 (95% UI: 3100–28,000); 1.4% (95% UI: 0.4%–3.3%) Reduction in number of children aged 5–17 y with overweight: 35,000 (95% UI: 9000–81000); 1.1% (95% UI: 0.3%–2.5%)	[101]

Abbreviations: CDC, Centers for Disease Control and Prevention; CI, confidence interval; CLF, Center for a Livable Future; DiD, difference-in-differences; FFMI, fat-free mass index; FMI, fat mass index; MVPA, moderate or vigorous physical activities; NAICS, North American Industry Classification System codes; NQ-P, preschool children's Nutrition Quotient; NYC, New York City; OR, odds ratio; PA, physical activity; pBMI, BMI percentile; PR, prevalence ratio; RefUSA - 2 NAICS, data extracted using primary and secondary NAICS codes from the ReferenceUSA database that were assigned to businesses; RR, relative risk; RRR, relative risk ratio; UI, uncertainty interval; WHZ, weight-for-height *z*-score; % BF, percentage of body fat.

∗ *P* < 0.05.

**FIGURE 2:**
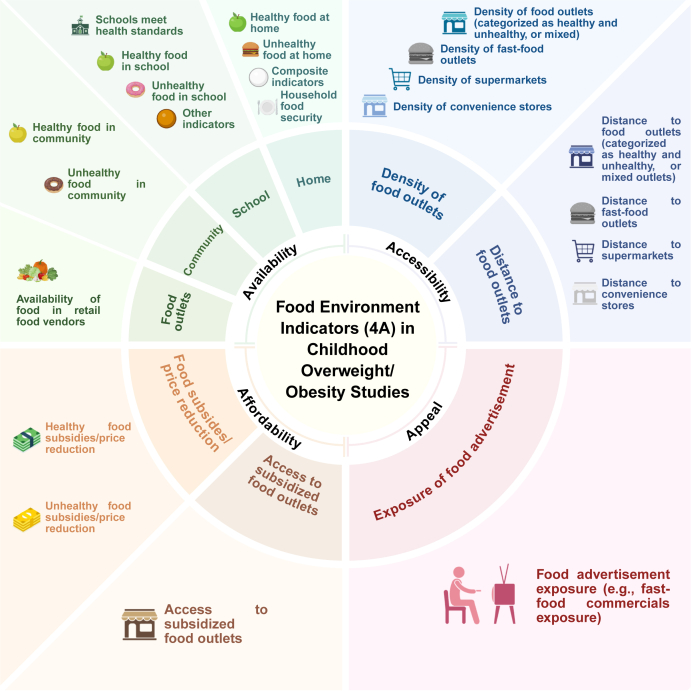
Food environment indicators used in childhood overweight/obesity studies (divided into 4A framework: food availability, accessibility, affordability, and appeal). In availability, indicators are classified into 4 categories based on settings: home, school, community, and food outlets. In accessibility, indicators are divided into 2 categories: density of food outlets and distance to food outlets, and 4 mainly primary indicators are listed in each category. In affordability, food subsides price reduction and access to subsidized food outlets are summarized. Exposure of food advertisement is the only domain in appeal (figure designed on https://www.biorender.com/).

This review organized the identified indicators according to the 4A framework, comprising food availability, accessibility, affordability, and appeal. The findings suggest that the existing evidence base is predominantly concentrated on food availability and accessibility with childhood overweight/obesity, whereas indicators related to affordability and appeal remain underexplored in the current literature. The representative indications reviewed are shown in Figure 2.

This review concentrated on the associations between food environment indicators and childhood overweight/obesity. Overweight/obesity was defined by BMI percentiles (pBMI; e.g., ≥85th and <95th percentile for overweight, ≥95th percentile for obesity) [28,31,39,[42], [43], [44],^51^,^57^,^66^,^82^], BMI-for-age *z*-scores exceeding specific standard deviations (e.g., >+1 SD for overweight and >+2 SD for obesity) [32,35,40,41,46,48,50,59,61,65,89], the International Obesity Task Force sex- and age-specific thresholds [27,56,73,79,80,88], or absolute BMI thresholds (e.g., ≥25 kg/m^2^) [28]. Additionally, overweight/obesity prevalence was commonly used as an outcome indicator in ecologic studies, longitudinal studies, and cross-sectional study [49,71,75,81,93,97]. Furthermore, continuous variables included BMI *z*-score [53,54,58,60,61,64,70,83,85,90,102], BMI [30,38,47,69,74,78,84,91,99,100], pBMI [33,52], SD Scores for child BMI [34,68], and body fat percentage [53,61,63,78,102], which were used to evaluate childhood overweight/obesity. Some studies also focused on longitudinal changes in continuous measures, analyzing outcomes such as changes in BMI *z*-score and BMI slope trajectories over time [85,87,92]. In addition to general obesity indicators, several studies explored associations between food environment indicators and abdominal obesity measures, including waist circumference [61,102], weight-for-height *z*-score, and abdominal fat percentage [53].

### Associations between food availability indicators and childhood overweight/obesity

Food availability indicators extracted from the included studies are summarized in Table 1. Summary of the associations between food environment indicators and childhood overweight/obesity is shown in Table 2. On the basis of the different settings and contents of food availability assessments across studies, we categorized these indicators into the following domains: availability of food at home, household food security, availability of food in schools, availability of food in the community, and availability of food in retail food vendors. For the domain "availability of food at home," we further distinguished whether the studies focused on healthy foods, unhealthy foods, or composite indicators incorporating both. Similarly, indicators related to food availability in schools and communities were classified based on whether they targeted healthy or unhealthy foods. Additionally, for school food availability indicators where studies did not specify the healthfulness of foods, these were grouped under "other indicators."

**TABLE 2:** Summary of the associations between food environment indicators and childhood overweight/obesity

Food environment dimensions	Increase the risk of childhood overweight/obesity	Decrease the risk of childhood overweight/obesity	Paradoxical
1A: Food availability	• Household food security • Availability of food in retail food vendors	• Healthy food at home • Schools meet health standards • Healthy food in school • Other indicators in school • Healthy food in community	• Unhealthy food at home • Composite indicators • Unhealthy food in community
2A: Food accessibility	• Density of unhealthy food outlets • Density of mixed food outlets • Density of convenience stores • Distance of healthy food outlets	• Density of healthy food outlets • Distance of unhealthy food outlets • Distance of mixed food outlets • Distance to fast food outlets • Distance to convenience store • Distance to corner store • Distance to restaurants • Distance to snack and beverage stores • Distance to nutrition assistance program locations	• Density of fast food outlets • Density of supermarkets • Density of farmer's markets/fruit and vegetable stores • Density of restaurants • Density of grocery stores • Density of nutrition assistance program locations • Distance to supermarkets
3A: Food affordability	• Unhealthy food subsidies/price reduction	• Healthy food subsidies/price reduction • Access to subsidized food outlets	**/**
4A: Food appeal	• Food advertisement exposure	**/**	**/**

#### Food availability at home

Under the indicator of healthy food availability at home, 1 cross-sectional study involving 12,041 children across 6 European countries found that rarely or never having fruits and vegetables available at home was significantly associated with childhood overweight/obesity [odds ratio (OR): 1.56; 95% confidence interval (CI): 1.07, 2.28] compared with always or often having fruits and vegetables available at home [27]. Another prospective observational study conducted in Poland among 506 children aged 5–11 y assessed both parental and children's perceptions of healthy food availability at home, and the result indicated that parents from dyads with excessive body mass reported lower availability of healthy food at homes, in contrast to those from dyads with normal body mass [29].

The availability of unhealthy foods at home primarily focused on items such as sweets, (salty) snacks, and sugar-sweetened beverages (SSBs) [27,30]. In a United Kingdom study, LeCroy et al. [30] investigated the association between the variety and quantity of SSBs available in the homes of 18 mo olds and childhood overweight/obesity. The results showed that medium household quantity of SSBs (>654 to ≤1747 mL) was significantly associated with lower BMI in children at 36 mo (*β*: –0.31; 95% CI: –0.58, –0.04) compared with low quantity (≤654 mL), and no significant associations were observed between children's overweight/obesity and high quantity or high variety of snack foods in home food availability [30]. In a retrospective longitudinal observational study carried out in the United States, a broader range of food items was included under the category of unhealthy food choices, but it did not identify statistically significant interactions between time and weight status regarding the availability of unhealthy food at home [31].

Some studies utilized composite indicators to capture both healthy and unhealthy food availability within the home environment. These included the Unhealthy Food Index [33], Home Food Environment Composite [34,[37], [38], [39]], Obesogenic Home Food Environment Score [35], and the individual components of the NQ-P (preschool children's Nutrition Quotient) score-Environment [36]. A cross-sectional study conducted among 1674 children in Indonesia found that a higher Obesogenic Home Food Environment Score had a significant association with overweight/obesity (OR: 1.11; 95% CI: 1.00, 1.23), as well as with increased BMI *z*-scores at the 75th percentile (*β*: 0.109; *P* < 0.05) and 90th percentile (*β*: 0.110; *P* < 0.05) [35]. Another cross-sectional study carried out in Korea among children aged 3–5 y showed that the highest score in the individual components of NQ-P score-Environment was associated with lower risk of overweight or obese status (OR: 0.36; 95% CI: 0.17, 0.71) [36]. Additionally, 1 cross-sectional study [37] and 1 case-control study [39] reported significant associations between Home Food Environment Composite scores and overweight/obesity, whereas other 2 studies did not find significant outcomes [34,38]. What is more, 1 cross-sectional study conducted in the United States among children and adolescents aged 6–17 y also revealed no significant association between Unhealthy Food Index and pBMI [33].

Three studies investigated the association between household food insecurity and childhood overweight/obesity, also employing various measurement tools, including the Household Food Insecurity Access Scale (HFIAS) [40,41], and the Children's Food Security Scale survey module [41]. Two studies found that higher levels of household food insecurity were associated with a heightened risk of childhood overweight/obesity [40,42]. What is more, a cross-sectional study carried out in Ethiopia involving children aged 5–18 y did not find a significant association between household food insecurity and overweight/obesity, as assessed by the HFIAS and the Children's Food Security Scale survey module [41].

#### Food availability in school

Schools were defined and classified according to specific institutional or health-related standards, such as distinguishing conventional schools from anthroposophical schools, designating childcare setting as "healthy," or evaluating the actions carried out in schools (e.g., School Heath Program) [[43], [44], [45], [46]]. A cross-sectional study conducted in Israel revealed that compared with conventional schools, anthroposophical schools had lower proportions of severe obesity, obesity, and overweight among students [44]. Anthroposophical schools emphasize a holistic educational and lifestyle philosophy that integrates health-promoting and food-related practices into daily routines when compared with conventional schools [44]. These schools emphasize fresh and minimally processed foods, often provide vegetarian meals, and foster consistency between school and home food environments through food-related education and parental engagement [44]. Besides, 2 additional studies examined the association between the percentage of healthy preschools [43] and the implementation of School Health Program (including healthy eating promotion activities, nutritional state assessment in the school, and childhood obesity prevention activities) [45] and childhood overweight/obesity, no statistically significant result was found except having nutritional state assessment in the school and childhood overweight in Brazil (OR: 0.66; 95% CI: 0.45, 0.96) [45]. The cross-sectional study conducted in the United Kingdom defined "healthy childcare settings" according to the criteria established by Public Health Wales, as those committed to providing healthy foods such as fresh fruits and vegetables and ensuring dedicated time for physical activities [43]. A cross-sectional study carried out in Brazil among 29,024 adolescents aged 12–17 y found that coverage by laws restricting the sale of certain foods and beverages in school cafeterias was associated with a lower likelihood of adolescent obesity (OR: 0.89; 95% CI: 0.88, 0.91) [46].

Among studies conducted in school settings examining the association between food availability indicators and childhood overweight/obesity, the types of exposures were categorized as healthy food, unhealthy food, and others. Four studies focused on healthy food availability in schools [28,37,47,48], with most of them concentrating on the provision of fruits and/or vegetables. For example, a cross-sectional study involving 3148 elementary, middle, and high school students in Korea found that agreement with the statement "the school provided fruit twice a week" was associated with a reduced risk of overweight in elementary school girls (agree: OR: 0.54; 95% CI: 0.31, 0.95) [28]. Betts et al. [47] assessed both fruits and vegetables availability in school and access to all food outlets in the United States, and reported no significant association with children's BMI, irrespective of the inclusion of food outlet accessibility in the models [47]. Another study of 2530 adolescents aged 12–17 y in Brazil reported that a higher number of operational drinking fountains in schools was associated with a reduced prevalence of obesity [48].

In school settings, the assessment of unhealthy food availability indicators was similar to that in household environments, primarily focusing on the provision of SSBs, (salty) snacks, and sweets. Betts et al. [47] investigated whether the association between the availability of school snacks and soda and children's BMI was moderated by all food outlets access in the United States, but no significant result was found. Two studies examined the association between the availability of unhealthy food in schools and overweight/obesity, and identified that neither reported a significant association [37,47].

In the category of other school-based indicators, 3 studies explored the association between various school-provided meal-related variables and overweight/obesity in children [32,37,45]. These variables included: the quantity of competitive foods and beverages (CF&Bs) provided to students during the school day, excluding federally reimbursable and nutritionally regulated school meal programs, including items sold in vending machines, school stores, canteen, snack bar or a la carte items from the cafeteria [37]; whether students purchased food from the school bars, which were operated by small independent vendors and sold CF&Bs [32]; whether students obtained food from the School Breakfast Program (SBP) [32]; and the number of meals offered at school [45]. One study constructed a joint variable that simultaneously considered the healthfulness of the school food environment and the density of the surrounding community food environment (categorized as low or high density) in the United States, and the result showed that unhealthy school food environment combined with high-density community food environment was associated with higher obesity rate at baseline, but no significant result was seen in the difference in obesity rate trend [49]. Another study found that obtaining food from the SBP was notably associated with a reduced likelihood of obesity among primary school students (OR: 0.76; 95% CI: 0.63, 0.91) compared with those who did not [32]. Similarly, offering 3 meals/d at school, compared with 1 meal, was associated with a diminished risk of overweight from a study conducted in Brazil (OR: 0.62; 95% CI: 0.47, 0.81) [45].

#### Food availability in community

In the context of community settings, we similarly continued to categorize food availability into healthy and unhealthy food types. However, unlike studies conducted in home or school environments, most community-based research did not focus on the availability of specific food items. Instead, composite indicators were commonly used to assess food availability, incorporating multiple food types, survey questions, or scales that characterize the healthfulness or unhealthfulness of the community food environment. These indicators often reflected multiple dimensions of food access [39,49,50]. Additionally, several countries have implemented nutrition assistance programs, including the Special Supplemental Nutrition Program for Women, Infants and Children (WIC) in the United States, to improve food access among vulnerable populations and support healthier eating behaviors in children [51]. A retrospective cohort study involving 148,634 children in the United States evaluated the association between the modified Special Supplemental Nutrition Program for WIC food package and childhood obesity, and the result found that compared with the old WIC package, the new package was associated with a reduced risk of obesity at age 4 in both boys and girls whatever at median healthy and unhealthy food environment densities or at the healthiest food outlet density [51].

#### Food availability in food outlets

Three studies investigated food availability within the context of retail environments [50,53,54]. A study conducted in Brazil among 665 children aged 6–59 mo employed the AUDITNOVA instrument to assess the healthiness of the consumer food environment. The findings indicated that a low healthiness score was significantly associated with overweight (OR: 1.69; 95% CI: 1.05, 2.73), and higher availability of ultraprocessed foods in the consumer food environment was also substantially associated with an increased likelihood of overweight (OR: 2.64; 95% CI: 1.61, 4.33) [50]. Another cross-sectional study conducted in Mexico among 218 children aged 8–10 y examined food access within convenience stores located within a 250-m buffer zone. This study specifically assessed shelf space allocated to different types of food, and the results showed that both shelf space for processed foods (m) (*β*: 0.002; 95% CI: 0.000, 0.003) and for non- or minimally processed foods (m) (*β*: 0.072; 95% CI: 0.009, 0.135) were significantly associated with increased BMI *z*-scores [53]. Johnson et al. [54] assessed the healthfulness of foods offered in retail stores in the United States utilizing the Healthy Food Availability Index score, but no significant result with children's BMI was found.

### Associations between food accessibility indicators and childhood overweight/obesity

Research examining the association between food accessibility and childhood overweight/obesity primarily focused on 2 dimensions of exposure: the density and proximity of various food outlets, with children's schools or homes as the reference location [49,56]. Density typically refers to the presence or number of food outlets within a specified buffer zone around a school or residence, whereas distance usually refers to the (nearest) distance to a specific type of food outlet [57,52]. Studies varied in their choice of buffer sizes, with commonly used distances including 200 m, 400 m, 500 m, 800 m, 1 km, 3 km, 0.25 mile, 0.5 mile, and 1 mile [49,56,58,66,77,83,88]. Some studies also applied less conventional buffers such as 0.025 mile, 0.05 mile, 0.1 mile, 3 mile, and 5 mile [85,94]. In terms of food outlet types, many studies utilized composite indicators that categorized multiple outlet types as healthy, unhealthy, or without health classification [47,55,57]. In addition to composite classifications, several studies focused on individual outlet types, most frequently including fast food outlets, supermarkets, convenience stores, and grocery stores [43,56,85,86]. A smaller number of studies examined other outlet types separately, such as farmers' markets/fruit and vegetable stores, corner stores, restaurants, nonalcoholic beverage stores, snack outlets, bakeries and cafeterias, pizzerias, small food retail stores, specialty food stores, and nutrition assistance program locations [65,83,84].

#### Density of food outlets

Three studies focused on the density of healthy food outlets [49,55,56]. For example, 1 cross-sectional study conducted in Brazil among 366 children aged 8–9 y found that, compared with their absence, the presence of healthy food stores (defined as stores where fresh or minimally processed foods accounted for >50% of total purchases) was significantly associated with reduced odds of childhood obesity when located within a 200-m buffer around schools (OR: 0.37; 95% CI: 0.17, 0.82), within a 200-m buffer around households (OR: 0.30; 95% CI: 0.11, 0.79), and within a 400-m buffer around households (OR: 0.19; 95% CI: 0.06, 0.59) [55].

Ten studies assessed the density of unhealthy food outlets [52,[55], [56], [57], [58], [59], [60], [61], [62], [63]], of which 7 reported statistically significant associations. Of 11 studies, 7 assessed the association of unhealthy food outlets with childhood overweight/obesity using indicators such as presence, number, or the density of outlets [[55], [56], [57], [58], [59], [60], [61]]. Beyond traditional measures, such as food outlet density, several studies have employed alternative conceptualizations, such as identifying "food deserts" [52] or utilizing composite measures such as the Obesogenic Environment Score [62,63], to capture the complexity of food environments. For example, a cross-sectional study involving 1296 children aged 5–9 y in the United States examined residence in food deserts (yes/no) and found that among food-insecure households, living in a food desert was positively associated with child pBMI (*β*: 5.723; 95% CI: 0.560, 10.887), whereas the association became nonsignificant among food-secure households [52]. Moreover, an ecologic study conducted in the United Kingdom across 6791 middle layer super output areas found that a higher Obesogenic Environment Score was consistently associated with increased risks of childhood overweight/obesity [62].

Nine studies included multiple types of food outlets without classifying them as healthy or unhealthy [47,48,55,[64], [65], [66], [67], [68], [69]]. Of 9 studies, 4 reported statistically significant associations with overweight/obesity. For example, for 1 cross-sectional study carried out in Mexico involving 2239 adolescents aged 12–19 y, this study found that the density of all stores was associated with overweight/obesity (*β*: 0.007; 95% CI: 0.002, 0.012) [65]. Of 9 studies, 5 reported no significant results. For example, Amram et al. [67] employed the Modified Retail Food Environment Index (%) in the United States to assess the overall retail food environment quality, but no significant results were found. What is more, Gardone et al. [55] classified "mixed food stores" as those where culinary preparations or processed foods predominated, or where there was no clear predominance of fresh/minimally processed or ultraprocessed foods, conducted in Brazil, and found no significant association with obesity outcomes. Additionally, de Assis et al. [48] examined the quantity of ready-to-eat food shops (including diners, snack shops, bars, restaurants, supermarkets, hypermarkets, and grocery stores) within an 800 m buffer around schools in Brazil as the exposure variable, and the findings indicated that the density of such food outlets was consistently associated with an increased risk of obesity.

A total of 19 studies examined the association between the density of fast food outlets and childhood overweight/obesity [43,49,54,56,66,[70], [71], [72], [73], [74], [75], [76], [77], [78], [79], [80], [81], [82]], among these 14 studies reported statistically significant results. For example, a longitudinal study carried out in the United States among 361,942 high school students aged 9–12 y explored the proximity of fast food restaurants within <0.25 miles and 0.25–0.50 miles of students' homes and its association with adolescent obesity, and the findings indicated that both <0.25 miles (*β*: –0.013, predicted likelihood: 0.152) and 0.25–0.50 miles (*β*: –0.025, predicted likelihood: 0.153) were associated with an heightened risk of obesity [77]. Three studies explored the change in fast food exposure as an independent variable [70,78,79], whereas Dolton and Tafesse [74] considered fast food outlet operating hours also as an indicator of exposure. Among these, Molenberg et al. [78] found that an increase in fast food exposure over time was significantly associated with higher fat mass index among children with lower maternal education levels in the Netherlands (*β*: 0.04; 95% CI: 0.00, 0.08; *P* < 0.05), highlighting potential socioeconomic disparities in vulnerability to obesogenic environments.

A total of 10 studies concentrated on supermarkets as the food outlet of interest [57,58,65,71,72,77,[82], [83], [84], [85]], with 6 studies reporting statistically significant findings. For instance, a cohort study conducted in the United States involving 28,359 children found that living in low-income, low-food access neighborhoods during pregnancy, characterized as low-income areas where the closest supermarket is >0.5 miles away in urban areas or >10 miles in rural areas, was significantly associated with increased BMI *z*-scores and elevated risks of obesity and severe obesity at ages 5, 10, and 15 [85]. Rummo et al. [83] investigated the exposure to a Food Retail Expansion to Support Health (FRESH) supermarket within 0.50 miles of children's residences in the United States, and the result found that exposure to FRESH supermarkets, whether newly opened or renovated, was associated with lower BMI *z*-scores.

Eleven studies examined the density of convenience stores [53,56,65,71,72,75,82,84,[86], [87], [88]], of which 6 studies found significant associations. For example, a cross-sectional study in China involving 2201 children revealed that for every 10 additional convenience stores located near schools, the odds of childhood obesity increased by 13% (OR: 1.13; 95% CI: 1.03, 1.24). Moreover, children attending schools near 24 or more convenience stores had notably higher probabilities of obesity than those with fewer than 24 stores nearby (OR: 1.49; 95% CI: 1.09, 2.03) [86].

Other studies also examined farmer's markets/fruit and vegetable stores [66,71,82,84], restaurants [56,66,71,72,77,89], grocery stores [56,58,65,87,89], nonalcoholic beverage store [65], bakeries and cafeterias [66], pizzerias [66], snack outlets [89], small food retail stores [84], specialty food stores [84], nutrition assistance program locations [54,83] as food outlet types related to childhood overweight/obesity. The evidence regarding the association between the density of farmer's markets/fruit and vegetable stores, grocery stores, and restaurants and childhood overweight/obesity was mixed (Table 1).

#### Distance to food outlets

Regarding distance to healthy food outlets, 2 studies assessed healthy food outlets access [56,90]. For example, Jiang et al. [90] found that healthy food access (ample compared with low) was associated with lower BMI *z*-scores among children, regardless of residential mobility status, including both nonmovers and movers. In contrast, 4 studies investigated the distance to unhealthy food outlets [56,91,52,92], among which 1 study reported a statistically significant association [91]. Specifically, Andres et al. [91] evaluated limited food access in the United States, defined as having at least one-third of the population, or ≥200 individuals, living outside the service area of a grocery store (>1 mile for urban block groups and >10 miles for rural block groups), and the result found that limited food access significantly increased the risk of elevated BMI (*β*: 3.27; 95% CI: 0.31, 6.22) and BMI as a percentage of the 95th percentile (*β*: 12.4; 95% CI: 0.24, 24.56) [91]. Only 1 study assessed food outlet distance without categorizing the outlets by healthfulness [64].

Eight studies examined the association between distance to fast food outlets and childhood overweight/obesity [56,70,74,80,81,[93], [94], [95]]. Among these, 4 studies reported statistically significant associations. A large longitudinal study conducted in the United States involving 1,188,658 students found that, compared with living within 0–0.025 miles of the nearest fast food outlet, living within 0.025–0.05 miles or 0.05–0.1 miles was significantly associated with reduced risk of overweight and obesity [94]. Moreover, Smagge et al. [81] further disaggregated fast food outlet types into fast food restaurants, grillrooms & kebab shops, and takeaway restaurants; the result shows that a greater distance to grillrooms and kebab shops was associated with reduced prevalence of overweight, whereas shorter distances to fast food restaurants and takeaway restaurants were paradoxically associated with lower overweight rates in the Netherlands.

Three studies investigated the association between distance to supermarkets and childhood overweight/obesity [93,96,97], with 2 studies reporting statistically significant findings. A study involving 6772 United Kingdom children aged 4–5 y assessed the log-transformed road distance from the postcode centroid to the nearest supermarket, and found that a greater distance was significantly associated with higher obesity rates across both England overall and urban areas specifically [97]. In another study conducted in the United Kingdom, Titis evaluated various distance measures, road distance, Euclidean distance, walking distance, cycling distance, and driving distance, and instead found that all distance metrics except Euclidean distance were positively associated with the proportion of overweight children [96].

A total of 4 studies investigated convenience stores as the food outlet type [53,56,88,93], among which 3 reported statistically significant findings. For instance, a cross-sectional study conducted in Mexico among 218 children aged 8–10 y examined the convenience store proximity (in meters) on body fat percentage (*β*: –0.009; 95% CI: –0.017, –0.001), abdominal fat percentage (*β*: –0.012; 95% CI: –0.023, –0.001), and BMI *z*-score (*β*: –0.002; 95% CI: –0.004, –0.001), and found that increased distance to the nearest convenience store was significantly associated with lower values in all 3 outcomes [53]. Similarly, studies using mean distance to fast food outlets [93] and the presence of convenience stores within 1000 m [88] as exposure variables also revealed significant associations with childhood overweight/obesity. However, 1 study measuring the shortest distance from the participant's residential neighborhood to the nearest convenience store within an 800-m buffer in Norway did not identify a statistically significant association [56].

In addition to the 3 commonly studied types of food outlets, several other outlet types have been examined in relation to distance-based measures, including grocery stores [93,56], corner stores [94], specialty stores [93], restaurants [56,93], snack and beverage stores [93,98], farmers' markets [93], food assistance providers [93], and nutrition assistance program locations [93]. In a longitudinal study in the United States involving 1,188,658 students, Elbel et al. [94] found that a greater distance to the nearest corner store (0.05–0.1 miles buffer compared with 0–0.025 miles buffer from home) was significantly associated with lower obesity prevalence (percent change: –2.1%; *P* < 0.05) and BMI *z*-score (percent change: –2.4%; *P* < 0.01).

### Associations between food affordability indicators and childhood overweight/obesity

The affordability dimension of the food environment primarily considers the price of food, including subsidies and promotional sales, as well as national-level nutrition programs that aim to make healthy food more financially accessible, through supermarket-based interventions targeting local residents. One study examined the association between healthy food subsidies or price reductions and overweight/obesity. A longitudinal study conducted across 106 schools in the United States used linear regression models with interaction terms to assess obesity trends over time. This study found that schools with a higher percentage of students eligible for free or reduced-price meals was associated with a faster increase in the prevalence of overweight and obesity (*β*: 0.095; *P* < 0.05), after controlling for the National School Lunch Program healthy scale, the competitive food healthy scale, school level (elementary compared with middle and high schools), and the proportion of enrolled students from different racial/ethnic groups [49].

Only 1 study investigated the association between subsidies or price reductions for foods, specifically healthy foods including fried snacks, candy, and sweetened beverages, in school surroundings and overweight outcomes, and the findings indicated that greater availability of fried snacks (OR: 1.38; 95% CI: 1.05, 1.81) and candy (OR: 1.58; 95% CI: 1.11, 2.27) was significantly associated with a higher risk of overweight, but no significant result was observed between sweetened beverage sales in the school environment and overweight [45].

Only 1 study examined the association between the exposure to a FRESH supermarket, either newly built or renovated, within 0.50 miles and childhood overweight/obesity among 54,728 residentially stable public-school students in the United States. The result found that exposure to any type of FRESH supermarket was significantly associated with reductions in BMI *z*-score, including all FRESH supermarkets [difference-in-differences (DiD): –0.04; 95% CI: –0.06, –0.02], newly built supermarkets (DiD: –0.07; 95% CI: –0.11, –0.03), and renovated supermarkets (DiD: –0.03; 95% CI: –0.06, –0.01). In terms of obesity likelihood, significant associations were observed only for exposure to all FRESH supermarkets and to newly built ones, not renovated ones [83].

### Associations between food appeal indicators and childhood overweight/obesity

Three studies explored the association between food appeal and childhood overweight/obesity, specifically focusing on children's exposure to food advertising [[99], [100], [101]]. A modeling study conducted in the United Kingdom, involving 13,729,000 children aged 0–17 y, assessed the exposures to advertisements for high-fat, sugar, and salt (HFSS) foods and beverages during different time slots. The study estimated that restricting HFSS advertising between 05:30 and 21:00 in the United Kingdom could prevent ∼40,000 [95% uncertainty interval (UI): 12,000–81,000] cases of obesity and 120,000 (95% UI: 34,000–240,000) cases of overweight among children aged 5–17 y. Shifting HFSS advertising to between 21:00 and 05:30 was predicted to lower obesity by 12,000 (95% UI: 3100–28,000) and overweight cases by 35,000 (95% UI: 9000–81,000) [101]. Additionally, in a cross-sectional study involving 2260 children aged 7–11 y, no significant association was found between exposure to commercial or noncommercial TV advertisements and BMI [100]. However, a longitudinal cohort study conducted in the United States among adolescents aged 13–16 y found that functional brain connectivity in response to unhealthy fast food commercials was significantly associated with BMI measured at a 2-y follow-up [99].

### Food environment indicators in intervention studies

A total of 6 intervention studies were identified that incorporated components of the food environment in addressing childhood overweight/obesity [[103], [104], [105], [106], [107], [108]] (Table 3). These studies varied in design, target population age groups, and geographic context. Although these studies included components addressing the food environment, most of the identified studies not exclusively targeted modifications in the food environment as the sole intervention strategy when evaluating the impact on childhood overweight/obesity.

**TABLE 3:** Effects of food environment indicators on childhood overweight/obesity from intervention studies

Study design	Country	Age	No. of participants	Food environment intervention strategy	Overweight/obesity indicators	Results	Ref.
Randomized clinical trial	United States	8.0 and 12.9 y of age, 10.4 y (SD: 1.3)	137 children (49 boys and 88 girls)	Change in Home Food Index-High Fat (1 SD) (high in fat content: >45% energy from fat; 14 items); Change in Home Food Index-Low Fat (1 SD) (low in fat content: <18% energy from fat; 12 items)	Child weight loss rate	High fat (estimate: 1.101, 95% CI: 0.168, 2.033, *P* = 0.021)∗ Low fat (estimate: –3.733, 95% CI: –5.674, –1.791, *P* = 0.001)∗	[104]
Randomized controlled pilot trial	United States	12–16 y old	82 children (30 boys and 52 girls)	The behavioral weight loss (BWL) treatment included guidance on improving the home food environment and dietary quality; Guidance on modifying the home food environment included: removing energy-dense, nutrient-poor items; replacing these with nutrient-rich foods; making nutrient-rich foods easily accessible and visible The parent weight loss (PWL) intervention focused on parents' own weight loss and encouraged them to use stimulus control strategies, including modifying the home food environment to support their weight loss goals	BMI	Food-secure households: BMI (*β*: 0.1, SE: 0.03, *P* < 0.01)∗ Food-insecure households: BMI (*β*: –0.05, SE: 0.05, *P* = 0.31)	[105]
Quasi-experimental analysis	United Kingdom	All schoolchildren in reception (ages 4–5 y) and year 6 (ages 10–11 y)	425,715 children over the sample years	The implementation of the fast food outlet restriction policy by Gateshead Council in 2015: introduction of planning guidelines effectively banning any new fast food outlets; implementation of all 3 types of planning guidance	Overweight and obesity (OWOB) prevalence	Treat × post (Gateshead policy intervention in the most deprived quintile-Q1) OWOB (prevalence in year 6: 1.168, SE: 1.715) Treat × post (Gateshead policy intervention in the second most deprived quintile-Q2) OWOB (prevalence in year 6: –4.789, SE: 1.289, *P* < 0.01)∗ Treat × post (Gateshead policy intervention in the third most deprived quintile-Q3) OWOB (prevalence in year 6: –4.106, SE: 1.527, *P* < 0.01) ∗	[106]
Randomized controlled trial	United States	7–10 y old	114 children (42 boys and 60 girls)	NU-HOME Intervention Increasing the healthfulness of food available at home: involved promoting a greater presence of fruits and vegetables and a reduction in the availability of sugar-sweetened beverages and high-fat and high-sugar foods in the home	BMI *z*-score; Body fat percentage; Percent over 50% BMI; Incidence of increasing weight status	NU-HOME Intervention (compared with control) BMI *z*-score (adjusted difference: –0.06, 95% CI: –0.19, 0.07, *P* = 0.342) Body fat percentage (adjusted difference: –1.37, 95% CI: –3.17, 0.43, *P* = 0.135) Percent over 50% BMI (adjusted difference: –1.61, 95% CI: –4.29, 1.07, *P* = 0.235) Incidence of increasing weight status (OR: 0.49, 95% CI: 0.11, 1.91, *P* = 0.311)	[108]
Multicenter, clustered randomized controlled trial	China	6–13 y old	7110 children (3476 boys and 3634 girls)	Intervention for catering staff catering staff working in school canteens received 4 40-min sessions of lectures regarding nutrition and health. They were trained on how to prepare nutritional lunches, and a goal of reducing 2 to 5 g of oil intake per day per child was established. Furthermore, the menu of the school lunch cafeteria was evaluated, and suggestions on how to adjust food items were provided by health promoters Parental intervention Parents attended 2 40-minute sessions of lifestyle and health lectures, where they were trained to build home environments for a healthy lifestyle and encouraged to prepare healthy meals at home for their children. This intervention aimed to modify the food environment within the home Information display in schools In schools with diet interventions, Dietary Pagodas for Chinese people were displayed on the walls of all classrooms, illustrating how to eat a varied and well-balanced diet. Additionally, hard copies of dietary guidelines for Chinese were provided to each classroom within these schools	Change in clustered metabolic risk score (CMRS), calculated by summing the z-scores of 5 components: % BF, systolic blood pressure, fasting glucose, ratio of cholesterol to HDL, triglyceride	Comprehensive intervention (combined diet and physical activity) change in CMRS (mean difference: –0.49, 95% CI: –0.85, –0.14, *P* = 0.007)∗ Diet-only intervention change in CMRS (mean difference: –0.20, 95% CI: –1.22, 0.82, *P* = 0.69)	[107]
Randomized clinical trial	United States	6–12 y old	452 children with overweight or obesity (213 boys and 239 girls)	Assignment to family-based treatment vs. usual care: Changing eating behaviors within the family unit through education, self-monitoring, goal setting, and problem-solving related to food choices (traffic light eating)	Clinically meaningful BMI *z*-score reduction ≥0.25 for participating child	Family-based treatment: 27.0%, usual care: 9.3%, *P* < 0.001∗	[103]

Abbreviation: CI, confidence interval; NU-HOME, New Ulm at HOME; OR, odds ratio; % BF, percentage of body fat.

∗ *P* < 0.05.

A range of intervention studies targeting the food environment have been conducted across different settings, including the home, schools, and policy level. Home-based interventions primarily focused on modifying the availability of healthy compared with unhealthy foods. Strategies included parental education (e.g., Parents as Coaches and Parent Weight Loss programs), behavioral treatments (e.g., traffic light eating, stimulus control), and environmental restructuring (e.g., removal of energy-dense, nutrient-poor foods and increasing visibility of nutrient-rich options) [[103], [104], [105],^108^,^109^]. School-based interventions emphasized improving the nutritional quality of meals through catering staff training and nutrition education, as well as enhancing the school food environment through visual materials and classroom-based dietary guidance [107]. At the policy level, policy measures such as the fast food outlet restriction implemented by Gateshead Council in the United Kingdom aimed to limit exposure to unhealthy food outlets [106].

## Discussion

This review provides a comprehensive synthesis of recent evidence on food environment indicators in relation to childhood overweight/obesity. Drawing on a systematic review of the literature, we categorized food environment indicators according to the 4A framework, including food availability, accessibility, affordability, and appeal. A broad and diverse body of evidence underscores a growing global interest in assessing and improving the healthfulness of children's surrounding food environments.

The studies identified in the literature encompass populations ranging from birth to 19 y, allowing for a comprehensive understanding of how food environments influence children across all developmental stages. The majority of included studies were observational in nature, consisting primarily of cross-sectional and longitudinal designs, with a smaller number of ecologic and other study types. Some national-level nutrition programs, such as the Supplemental Nutrition Assistance Program and the Special Supplemental Nutrition Program for WIC, were included and discussed under observational studies in this review [93,51]. A limited number of intervention studies were identified; however, only 1 targeted food environment components exclusively at the policy level [106]. The remainder employed multicomponent interventions that combined food environment strategies with individual-level approaches such as dietary and nutrition education [103,105,[107], [108], [109]]. Policy-level interventions remain underevaluated; however, emerging evidence, such as that from Xiang et al. [106] suggests their potential effectiveness in reducing childhood obesity rates. Overall, this highlights a notable gap in the intervention literature, particularly the scarcity of trials that isolate the effect of food environment modifications on childhood overweight/obesity.

The distribution of food environment indicators derived from the literature varies across the 4A framework. Indicators related to availability and accessibility are the most frequently studied, whereas those addressing affordability and appeal remain underrepresented. Upon disaggregation by subdomains, only a few specific indicators, such as fast food outlet density under accessibility, have been widely examined; in contrast, most other indicators were assessed in a limited number of studies and yielded inconsistent findings, highlighting the need for further investigation. A substantial proportion of studies assessing availability and accessibility employed adapted composite indices, which pose significant challenges for synthesizing findings and conducting cross-study comparisons.

Within the food availability domain, studies on healthy foods have predominantly focused on fruits and vegetables, whereas studies on unhealthy foods have primarily investigated SSBs, salty snacks, sweets, and fried foods. These food categories have been consistently identified in prior research as strongly positively associated with childhood overweight/obesity [[110], [111], [112], [113]]. We found that several factors were associated with increased childhood overweight/obesity, including the availability of food in retail food vendors within the availability domain. In contrast, healthy food availability at home, household food security, healthy food provision in schools, other school-related indicators, and healthy food availability in the community were identified as protective factors. The results for unhealthy food availability at home, composite indicators at home, and unhealthy food availability in the community were paradoxical. The paradoxical findings regarding unhealthy food availability may be attributed to underreporting owing to social desirability bias, and limitations in the measurement tools used across studies [114].

In terms of accessibility, a greater number of studies have examined unhealthy food retail outlets, either through comprehensive assessments across multiple outlet types or through a focused analysis of single categories such as convenience stores and fast food outlets. By contrast, research on healthy food outlets has largely concentrated on supermarkets, with relatively few studies addressing either multiple healthy outlet types or single categories such as farmers' markets. Across the included studies, a higher density of unhealthy food outlets, mixed food outlets, convenience stores, and nutrition assistance program locations was positively associated with childhood overweight/obesity, whereas a higher density of healthy food outlets showed an inverse association. Evidence regarding the density of fast food outlets, supermarkets, farmers' markets/fruit and vegetable stores, restaurants, and grocery stores was paradoxical. The associations between distance to different food outlets and childhood obesity were similar, but greater distance to fast food outlets consistently showed a negative association, suggesting that closer proximity to fast food restaurants may be associated with higher obesity risk among children. The inconsistent results for certain categories such as supermarkets, farmers' markets, restaurants, and grocery stores may be explained by the heterogeneity in the types of foods sold, as these venues often offer both healthy and unhealthy options [115]. The paradoxical findings for fast food outlets density could be attributed to several factors. First, socioeconomic disparities across regions may play a role, as areas with a higher density of fast food outlets often differ in income levels, educational attainment, and degree of urbanization [[116], [117], [118]]. Reverse causation cannot be ruled out, fast food outlets may be more likely to locate in areas with higher consumers demand or pre-existing higher obesity prevalence. In addition, contextual variations in food retail composition, for example, differences in the proportion of fast food restaurants relative to other types of food outlets, may contribute to inconsistent results across studies [119]. Furthermore, variability in the measurement of density, including differences in buffer sizes, may influence the observed associations. Similarly, the positive association between the density of nutrition assistance program locations and childhood overweight/obesity may reflect underlying socioeconomic deprivation, as such programs were typically concentrated in lower-income neighborhoods where obesity risk tends to be elevated [120].

In the food affordability domain, unhealthy food subsidies or price reduction were associated with a higher risk of childhood overweight and obesity, whereas healthy food subsidies or price reductions and access to subsidized food outlets appeared to have an inverse association. Existing research has indicated that higher prices for fruits and vegetables were associated with higher BMI in children, whereas higher prices for soft drinks were linked to a reduced likelihood of being overweight [121,122]. Regarding appeal, the studies demonstrated generally consistent associations with childhood overweight/obesity. Children and teenagers were exposed to a mean of 10 advertisements for unhealthy foods and beverages per day via television in Spain [123]. Appeal influences purchasing and dietary behaviors, and its results may differ by sex [24,99,124]. Moreover, the dimension of appeal in children's food environments extends beyond television advertising to include front-of-package labeling, along with exposure to food advertising around clubs and school environments [24,125]. These areas remain underexplored and warrant further research. For affordability and appeal, the volume of relevant studies remains limited. In our review, affordability was typically assessed through food prices or subsidies, whereas appeal was narrowly defined in terms of exposure to television-based food advertisements. The challenge of directly correlating subsidies with children's health outcomes accounts for the scarcity of research regarding affordability. Health or consumption behavior measurements may be more pertinent. In the context of appeal dimension, a viable method for obtaining exposure profile is to resort to innovative data sources, such as companies specializing in market research and consumer behavior analytics [101].

Takeaway food outlets, a growing mode of food provision and exposure within the food environment, have become increasingly popular, particularly among younger generations. A study conducted among secondary school students in the United Kingdom (aged 11–14 y) found that over half of the students purchased fast food or takeaway meals at least twice per week [126]. In a survey among Chinese university students, 47.31% reported consuming takeaway food at least once per week, with 13.70% consuming it 4 or more times weekly [127]. Despite this trend, few studies have explored the association between takeaway food consumption and child health outcomes. Our review did not identify any studies directly examining the relationship between takeaway food and childhood overweight/obesity. However, evidence from adult populations suggests a potential link. For example, 1 research used the Fenland study, which involved 4791 participants [mean age: 51.0 y (SD: 7.2)] and found that takeaway outlets were linked with greater takeaway consumption and body fat [128]. Takeaway food is typically characterized by a high content of unhealthy items, such as SSBs [129]. Individuals who frequently order food online tend to have higher intakes of sweets, salty snacks, and fast food [130]. Emerging evidence also indicates that takeaway consumption may be influencing dietary patterns and behaviors among children. For instance, one study found that frequent takeaway consumption was associated with an increased risk of emotional overeating among children [131].

In conclusion, this review synthesized recent literature on the relationships between food environment indicators and childhood overweight/obesity. These indicators were systematically categorized using the 4A framework: food availability, accessibility, affordability, and appeal. By integrating research-based evidence, this review identifies key food environment indicators, which is useful to prevent and manage childhood overweight/obesity at home, schools, communities, and society. In addition, this systematic review could also provide a foundational reference for building a comprehensive, globally adaptable framework for the development of food environment monitoring systems tailored to children, better supporting public health policy and intervention efforts targeting overweight/obesity, also facilitating global sustainable development.

Healthy diets are crucial for achieving global nutrition goals and the sustainable development goals. The UNICEF Nutrition Strategy for 2020–2030 focuses on key areas of action, including improving children's food environments through public sector policy actions and private sector practices (UNICEF 2023). To eliminate all forms of malnutrition, FAO, UNICEF, and WHO launched the Healthy Diets Monitoring Initiative in 2022, calling for targeted actions to be guided through monitoring efforts [132]. Although several monitoring systems in the fields of nutrition and health have emerged in recent years, we did not find a system specifically designed to monitor children's food environments. The development of a dedicated monitoring system for children's food environments is essential, because the food environment of children is unique and context-specific, and the food environment monitoring system designed for them should take this uniqueness into account. In terms of living environments, it is essential to consider the community, home, and school/child care center where children access food. Indicators of food availability, such as the availability of fruits and vegetables, are challenging to monitor and collect at scale on a continuous basis when using households or schools as primary data collection sites. Alternatively, using data derived from the overall food supply within a specific geographic region may offer a broader, macrolevel perspective on the food environment. In addition to this, children interact with food differently based on their age. For younger children, such as preschoolers, their direct exposure to food sales is limited; instead, they primarily access food through school or daycare meal programs and the home food environment [133]. In contrast, older children, such as school-age children, have some purchasing power and are more directly exposed to unhealthy food options in food retail outlets. Within the context of food outlet environments, monitoring systems should also account for the emerging role of takeaway and online food delivery services, and their potential impact on children's dietary behaviors and health outcomes. Regardless of age, all children are susceptible to food marketing, which can influence their dietary behaviors. Thus, we recommend including the dimension of food appeal in the monitoring framework for children's food environments. Studies have shown that exposure to food marketing affects children's food purchasing behaviors, which, in turn, influences their dietary choices and contributes to outcomes like overweight/obesity [11,134,135]. The marketing of unhealthy foods also encourages children to frequently ask their caregivers for marketed products [136]. Children's exposure to food environments tends to focus more on downstream factors, such as food availability and marketing, with less emphasis on upstream factors like food production and processing. Therefore, future designs should prioritize focusing on these downstream factors, specifically encompassing the scenes where children have direct exposure and interaction. Finally, it would be beneficial for food environment indicators to be explicitly connected to concrete actions in food environment monitoring system for children, providing unambiguous evidence of improvement for policymakers and practitioners.

## Author contributions

The authors' responsibilities were as follows – YL, HL, DL: conceptualized the study; YL, ZY, HL: developed the methodology; YL: performed the investigation, wrote the original draft, did the visualization; CB, DL, JX: acquired funding, did the project administration; and all authors: performed critical review and editing, read and approved the manuscript.

## Data availability

The data supporting the findings of this study are available from the corresponding author on request.

## Funding

This systematic review received funding from UNICEF to the project Child Health and Development (Award no. 202502012-03).

## Conflict of interest

The authors report no conflicts of interest.
